# Substituted 1,2,3-triazoles: a new class of nitrification inhibitors

**DOI:** 10.1038/s41598-021-94306-1

**Published:** 2021-07-22

**Authors:** Bethany I. Taggert, Charlie Walker, Deli Chen, Uta Wille

**Affiliations:** 1 School of Chemistry, Bio21 Institute, The University of Melbourne, 30 Flemington Road, Parkville, VIC 3010 Australia; 2 Incitec Pivot Ltd, VIC, PO Box 54, North Geelong, 3215 Australia; 3 School of Agriculture and Food, The University of Melbourne, Parkville, VIC 3010 Australia

**Keywords:** Organic chemistry, Small molecules

## Abstract

Nitrogen (N) fertilisers amended with nitrification inhibitors can increase nitrogen use efficiencies in agricultural systems but the effectiveness of existing commercial inhibitor products, including 3,4-dimethylpyrazole phosphate (DMPP), is strongly influenced by climatic and edaphic factors. With increasing pressure to reduce the environmental impact of large-scale agriculture it is important to develop new nitrogen-stabilising products that can give reliable and consistent results, particularly for warmer climatic conditions. We synthesised a library of 17 compounds featuring a substituted 1,2,3-triazole motif and performed laboratory incubations in two south-eastern Australian soils. In the neutral (pH 7.3) soil, the compounds N002, N013, N016 and N017, which possess short non-polar alkyl or alkynyl substituents at the triazole ring, retained NH_4_^+^-N concentrations at 35 °C soil temperature to a better extent (*P* < 0.001) than DMPP. In the alkaline soil (pH 8.8) N013 performed better with regards to NH_4_^+^-N retention (*P* = 0.004) than DMPP at 35 °C soil temperature. Overall, our data suggest that substituted 1,2,3-triazoles, which can be synthesized with good yields and excellent atom economy through 1,3-dipolar cycloaddition from readily available starting materials, are promising nitrification inhibitors performing similar to, or better than DMPP, particularly at elevated soil temperatures.

## Introduction

The provision of food security for a constantly increasing population has become a major task for society. It is expected that by 2050 annual crop production needs to increase by almost 40% to ensure adequate food availability^1^. This challenge is intensified by climate shifts, which put stress onto locally adapted crops and larger agricultural systems. High application of nitrogen fertilisers is common in agricultural systems to maximise fertility and achieve optimal yields^2,3^. However, plants rarely assimilate more than 50% of applied fertiliser nitrogen. While in Australia the nitrogen use efficiencies (NUEs) fall anywhere between 6 and 59% depending on crop type^4^, globally NUEs have stayed at around 50% since the 1980’s^4,5^. Nitrate (NO_3_^-^) leaching and denitrification are two important pathways responsible for the loss of nitrogen from the plant/soil system, which lead to damaging eutrophication of surface waters and groundwater pollution and emission of nitrous oxide (N_2_O), a potent greenhouse gas. One of the current approaches to reduce these two losses and increase NUE is through the use of fertilisers amended with nitrification inhibitors (NIs), which inhibit nitrifying microbes in the soil responsible for the conversion of ammonium (NH_4_^+^) → NO_3_^-^ to increase the residence time of NH_4_^+^.

Many of the compounds with known nitrification inhibitory properties are heterocycles containing two or more adjacent N, O or S atoms, suggesting that NIs target the first step of the ammonia oxidation process that is catalysed by ammonia monooxygenase (AMO)^6^. Unfortunately, as a membrane-bound enzyme AMO loses its structural organisation and function upon isolation, preventing detailed studies of its mode of action. However, it is known that copper (Cu), and possibly also non-heme iron (Fe), are involved in its enzymatic functionality, indicating that NIs might inhibit AMO through (reversible) complexation of the Cu centre^7^.

**Figure 1:**
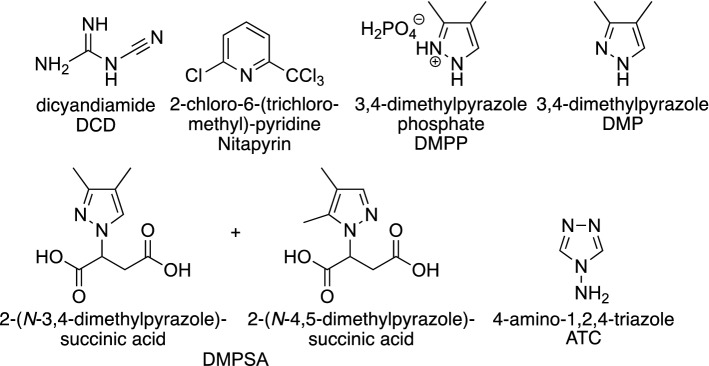
Selected N-containing nitrification inhibitors.

The most widely researched commercial NIs are based on one of three compounds dicyandiamide (DCD, AlzChem AG), 2-chloro-6-(trichloromethyl)-pyridine (Nitrapyrin or N-Serve®, Dow Chemical Co.) and 3,4-dimethylpyrazole phosphate (DMPP or ENTEC®, BASF AG) (Fig. 1).

The active ingredient in DMPP is 3,4-dimethylpyrazole (DMP), which is applied as the water-soluble phosphate salt. DMPP is the most promising NI to date, which has undergone extensive toxicological testing, is effective at low concentrations and has a low mobility in soils due to its positive charge^8^. Newer approaches to increase the lifetime of DMP-based inhibitors in soils involve reducing the volatility of the pyrazole core, for example by derivatisation of DMP with succinic acid to create the isomeric mixture of 2-(*N*-3,4-dimethylpyrazole)succinic acid and 2-(*N*-4,5-dimethylpyrazole)succinic acid (DMPSA)^9^.

Unfortunately, field studies with DMPP in different soils have shown an unreliable inhibitory activity with regards to reducing leaching and N_2_O emissions, ranging from no effect to inhibition as high as 90%^10–12^. Furthermore, the inhibitory activity of DMPP varies greatly with soil type^13^, moisture content^14^, soil pH^15^ and soil temperature, where a significant efficiency decrease was observed over a relatively small temperature change^16,17^.

Current approaches to improve inhibitor efficiency rely largely on modifying fertiliser formulations using DMPP, which cannot address the fundamental problems associated with this compound outlined above. Of the non-commercial NIs, the 1,2,4-triazole framework, particularly 4-amino-1,2,4-triazole (ATC, Fig. 1), appears promising^18^ with data indicating a better inhibitory performance than DMPP at higher soil temperatures and at relatively low application rates^17^. Unfortunately, synthesis of 1,2,4-triazoles often involves tedious, low-yielding multi-step processes, whereas recently developed one-pot syntheses are limited with regards to substitution pattern^19^.

As the exact structure of the active site in AMO is not known (see above), inhibitor design requires systematic structure–activity relationship (SAR) studies to guide further development. While investigations from the 1980s^18,20^ identified the inhibitory activity of N-containing aromatic compounds, to our knowledge, newer SAR studies involving more diverse structural frameworks that provide different chemical and physical properties are not available.

An important additional aspect governing NI development is that these compounds should be readily synthetically available to be of interest for large-scale production required in the agricultural industry. Supported by recent research that showed that DMPP can form chelated complexes with Cu in soils^21^, we explored the scope of the 1,2,3-triazole motif as nitrification inhibitor as we hypothesised that three adjacent nitrogen atoms in a planar, five-membered aromatic ring should increase the probability for successful complexation of the Cu centre in the active site in AMO.

**Figure 2:**
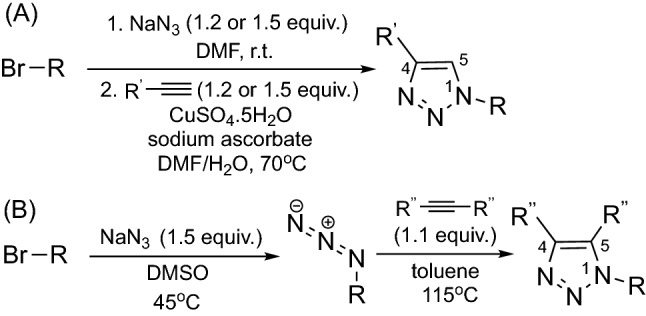
Synthetic pathways to the 1,2,3-triazoles studied in this work: (**A**) Cu(I) catalysed azide alkyne click reaction to give 1,4-disubstituted 1,2,3-triazoles; (**B**) thermal Huisgen 1,3-dipolar cycloaddition of an azide and an internal alkyne to provide 1,4,5-trisubstituted 1,2,3-triazoles.

1,2,3-Triazoles exhibit broad biological activity, which has been used in a wide range of applications, for example in drug discovery^22^. To our knowledge 1,2,3-triazoles substituted at the 1-position have not been tested as NIs so far, with the exception of benzotriazoles (structure not shown)^18,23^. Substituted 1,2,3-triazoles are synthetically accessible with good yields and high atom economy from a wide variety of readily available starting materials using copper-catalysed click chemistry^24,25^ or through the thermal Huisgen 1,3-dipolar cycloaddition^26^, as shown in Fig. 2.

Through variation of the substituents in the alkynes and azides, 1,2,3-triazoles with different substituents at the 1, 4 and/or 5 positions can be synthesized, which enables systematic SAR studies to assess the impact of properties such as polarity, hydrophilicity and lipophilicity on inhibitory activity in different soils. Such information could allow fine-tuning of the chemical characteristics to produce efficient inhibitors for different soil types.

In this work we have prepared a library of 17 di- and tri-substituted 1,2,3-triazoles and tested their performance as NIs in laboratory incubation studies with two different Australian soils. Several of these compounds outperform the commercial inhibitor DMPP at elevated soil temperatures, a key-finding that could provide new avenues to improve NUE in agriculture.

## Materials and methods

### Synthesis of nitrification inhibitors

#### Caution

Small organic azides are employed in this work and may be explosive. Whilst no problems were encountered by the authors, caution must be used when handling. Do not evaporate to dryness. Standard blast shields were used.

#### General information

Reaction progress was monitored by thin-layer chromatography (TLC) using silica gel 60 aluminium-backed plates coated with fluorescent indicator F254 (Merck). Plates were visualised using UV irradiation (254 nm) alone or in conjunction with ninhydrin-, potassium permanganate- or iodine-based stains. Purification by silica gel chromatography was performed using Davisil Chromatographic Silica Media LC60A 40–63 micron, with solvent systems as specified. All ^1^H and ^13^C NMR spectra were recorded on a 400 MHz Varian INOVA spectrometer (at 400 or 101 MHz, respectively) using solvent resonances as the internal standard (^1^H NMR: CDCl_3_ at 7.26 ppm, DMSO-d_6_ at 2.50 ppm; ^13^C NMR: CDCl_3_ at 77.0 ppm, DMSO-d_6_ at 39.5 ppm). Chemical shifts are reported in parts per million (ppm, δ), with the splitting patterns indicated as follows: s, singlet; d, doublet; t, triplet; q, quartet; p, pentet; h, hextet; m, multiplet; dd, doublet of doublets. The coupling constants, *J*, are reported in Hertz (Hz). Electrospray ionization high resolution mass spectrometry (HRMS) was performed on a Thermo Scientific Exactive Plus Orbitrap mass spectrometer (Thermo, Bremen, Germany) operated in positive mode.

Full synthetic procedures and characterisation data for all compounds are given in the Supplementary Information (SI). The structures of the substituted 1,2,3-triazoles synthesized and tested in this work are compiled in Table 1.

**Table 1:** Substituted 1,2,3-triazoles N001-N017 synthesized and tested as nitrification inhibitors (NI).


NI	R^1^	R^2^	R^3^	Properties of R^1^–R^3^
N001	*n*-C_5_H_11_	*n*-C_4_H_9_	H	Non-polar
N002	*n*-C_4_H_9_	*n*-C_4_H_9_	H	Non-polar
N003	*n*-C_4_H_9_	CH_2_C(=O)OC_2_H_5_	H	Non-polar; polar aprotic
N004	C(CH_3_)_2_OH	*n*-C_4_H_9_	H	Non-polar; polar protic
N005	*n*-C_4_H_9_	(CH_2_)_3_C(=O)OC_2_H_5_	H	Non-polar; polar aprotic
N006	*n*-C_4_H_9_	(CH_2_)_3_NH_2_	H	Non-polar; polar protic
N007	CH_2_OH	(CH_2_)_3_NPhth^[a]^	CH_2_OH	Polar protic; polar aprotic
N008	CH_2_OC(=O)CH_3_	(CH_2_)_3_NPhth^[a]^	CH_2_OC(=O)CH_3_	Polar aprotic
N009	CH_2_OH	(CH_2_)_3_C(=O)OC_2_H_5_	CH_2_OH	Polar protic; polar aprotic
N010	CH_2_OC(=O)CH_3_	(CH_2_)_3_C(=O)OC_2_H_5_	CH_2_OC(=O)CH_3_	Polar aprotic
N011	CH_2_OC(=O)CH_3_	CH_2_C(=O)OC_2_H_5_	CH_2_OC(=O)CH_3_	Polar aprotic
N012	CH_2_OH	CH_2_C(=O)OC_2_H_5_	CH_2_OH	Polar protic; polar aprotic
N013	*n*-C_3_H_7_	*n*-C_4_H_9_	H	Non-polar
N014	*n*-C_4_H_9_	(CH_2_)_2_OCH_3_	H	Polar protic; polar aprotic
N015	*n*-C_4_H_9_	(CH_2_)_2_OH	H	Polar protic; polar protic
N016	*n*-C_3_H_7_	*n*-C_3_H_7_	H	Non-polar
N017	*n*-C_3_H_7_	(CH_2_)_2_C≡CH	H	Non-polar

[a] Phth = phthaloyl.

#### General procedure A

Copper(I)-catalysed azide-alkyne cycloaddition (CuAAC) to synthesise 1,4-disubstituted triazoles.

Sodium azide (1.2 or 1.5 equiv.) was suspended in dimethylformamide (DMF, 0.85 M) under argon atmosphere, and to this the appropriate alkyl bromide (1 equiv.) was added. The solution was stirred at room temperature for 6–17 h. The reaction was quenched by the addition of  H_2_O (to make DMF/H_2_O 1:1 v/v), before the successive additions of copper(II) sulphate pentahydrate (CuSO_4_·5H_2_O, 0.06 equiv.), sodium ascorbate (0.3 equiv.) and the appropriate alkyne (1.2 or 1.5 equiv.). The reaction was heated at 70 °C overnight with vigorous stirring, cooled to room temperature, diluted with H_2_O (at least 3 × DMF volume) and extracted with ethyl acetate. The combined extracts were washed with 5% aqueous lithium chloride solution, concentrated and purified by silica chromatography.

#### General procedure B

Thermal Huisgen 1,3-dipolar cycloaddition to synthesise 1,4,5-trisubstituted triazoles.

Sodium azide (1.5 equiv.) and the appropriate alkyl bromide (1 equiv.) were charged into a flask flushed with argon, suspended in dimethyl sulfoxide (DMSO, 1.28 M) and warmed to 45 °C with vigorous stirring. After 20 h the reaction mixture was cooled to room temperature, quenched with H_2_O (to make DMSO/H_2_O 4:5 v/v) and extracted with diethyl ether. The combined ethereal extracts were concentrated under N_2_ flow to an oil, which was used directly in the subsequent step. CAUTION: Organic azides may be explosive, do not evaporate to dryness. Smaller azides were handled using solvent substitution, where toluene was added before diethyl ether was evaporated under N_2_ flow.

The crude azide was suspended in toluene (0.21 M) and the appropriate internal alkyne (1.1 equiv.) was added. The reaction was then heated at 115 °C with vigorous stirring. Once TLC indicated complete consumption of starting material (24–48 h) the reaction was cooled. Toluene was removed *in vacuo* to leave crude triazole as a waxy brown solid, which was purified by either recrystallisation or column chromatography.

### Soil microcosm experiments

#### Soil physicochemical properties and chemicals

Soil used in this study was collected from two different locations in Victoria, Australia: (1) a wheat cropping soil from Horsham (36°45’ S, 142°07’ E) and (2) a rotational cropping soil from Dahlen (36°37’ S, 142°09’ E). Soil pH was measured at a soil to water ratio of 1:5 (w/v) as pH 8.8 and pH 7.3, respectively. An overview about the properties of the two soils used in this study is given in Table S2 in the SI. The water content of the soils was calculated before commencing each experiment, from samples oven-dried to constant weight. The soil’s water-filled pore space (WFPS) was in the range 52–61% (details are provided in Tables S4-S5 in the SI), which is within the recommended 50–70% range for microbial activity due to oxygen and nutrient availability^27^.

3,4-Dimethylpyrazole phosphate (DMPP), prepared as a solution of 3,4-dimethylpyrazole in phosphoric acid, was obtained from Incitec Pivot Fertilisers.

#### Incubation experiments

Soils used in these studies were air-dried, ground and sieved (2 mm) prior to use. Soil microcosm incubations were carried out in 250 mL polypropylene specimen containers (Sarstedt, Germany), containing 18.24 g oven dry-weight equivalent of soil. Microcosms were treated with half the volume of water required to meet the desired water-filled pore space (WFPS%) and pre-incubated at 25 °C for seven days to revive soil microbial activity (see below). Following pre-incubation, the remaining volume to reach the WFPS% was applied as one of the treatment solutions: (1) ammonium sulphate ((NH_4_)_2_SO_4_,), (2) (NH_4_)_2_SO_4_ + DMPP, or (3) (NH_4_)_2_SO_4_ + one of the inhibitors N001- N017, with three replicates of each treatment per time interval (n = 3). Treatment solutions were prepared such that each microcosm received (NH_4_)_2_SO_4_ at a rate of 100 mg N per kg soil and inhibitors N001-N017 as well as DMPP at 10 mol% of the applied N (H-DMPP). Details are provided in Table S3 in the SI. It should be noted that in some initial screening experiments a lower DMPP application rate was used (L-DMPP: 1.5 mol% of the applied N; M-DMPP: 3.6 mol% of the applied N).

The microcosms of the soils were incubated in the dark for 28 days at 25 °C and at 35 °C, similar to other studies^17^. Throughout the incubation period soil microcosms were kept aerated by removing the lid for 10 min every 2–3 days to allow gas exchange, and moisture levels were replenished via addition of milli-Q water as required (e.g., every few days) based on weight loss. At the end of the desired incubation period (i.e., after 0, 3, 7, 14, 21 and 28 days, respectively), soil microcosms were removed and destructively sampled by treating with 2 M aqueous potassium chloride solution (KCl, 100 mL) and shaking for 1 h. Soil-KCl solutions were filtered (Whatman No. 42), and the filtrates were stored at − 20 °C until the conclusion of the experiment, when all KCl extracts were analysed for the concentration of soil mineral nitrogen from ammonium (NH_4_^+^-N) and from combined NO_3_^-^ and NO_2_^−^ N-N (NO_x_^-^-N) after appropriate dilutions using Segmented Flow Analysis (San++, Skalar, Breda, The Netherlands). Results are reported as the mean of three replicates, errors reported are standard errors of the mean. Errors associated with raw data were carried through calculations using standard error propagation protocols.

#### *Calculation of nitrification rates and NO*_*x*_^*–*^*-N production rates*

Nitrification was assessed based on both rate of NH_4_^+^-N loss and rate of NO_x_^-^-N accumulation. For each treatment NH_4_^+^-N loss was calculated as a percentage, according to Eq. (1):

1 $$ {\text{NH}}_{4}^{ + } - {\text{N}}\;{\text{loss}}\,{\text{(\%)}} = \frac{{[{\text{NH}}_{4}^{ + } - {\text{N}}]_{{{\text{t}} = 0}} - [{\text{NH}}_{4}^{ + } - {\text{N}}]_{{\text{t}}} }}{{[{\text{NH}}_{4}^{ + } - {\text{N}}]_{{{\text{t}} = 0}} }} \times 100 $$

where [NH_4_^+^-N]_t=0_ is the NH_4_^+^-N concentration (in mg N kg^-1^ soil) of the soil on day 0 and [NH_4_^+^-N]_t_ is the NH_4_^+^-N concentration (in mg N kg^-1^ soil) of the soil at a given time point, t.

NO_x_^–^-N accumulation rates (mg NO_x_^–^-N/kg soil/day) over the 28-day incubation experiments were calculated for each treatment, according to Eq. (2):

2 $${\text{NO}}_{{\text{x}}}^{ - } - {\text{N}}\;{\text{accumulation}} = \frac{{[{\text{NO}}_{{\text{x}}}^{ - } - {\text{N}}]_{{{\text{t}} = 28}} - [{\text{NO}}_{{\text{x}}}^{ - } - {\text{N}}]_{{{\text{t}} = 0}} }}{28}$$

[NO_x_^−^-N]_t=0_ and [NO_x_^–^-N]_t=28_ are the combined concentrations of NO_2_^−^ and NO_3_^-^ in the soil (in mg N kg^-1^ soil) on day 0 and day 28, respectively. It was not possible to assess whether the soil pre-incubation prior to application of the inhibitor treatments had any effect on the N transformations occurring in the soils. For example, concurrent N conversion processes other than nitrification could be triggered, such as immobilisation or (re)mineralisation of NH_4_^+^ or NO_x_^−^ or volatilisation of NH_3_, which could result in different rates for NH_4_^+^-N consumption than for accumulation of NO_x_^–^-N^17^. However, it has previously been shown that treatment with nitrification inhibitors itself has little effect on mineralisation and immobilisation^28^.

Nitrification inhibition (%) was determined based on loss of NH_4_^+^-N from the percentage of NH_4_^+^-N loss of the fertilised control (treatment with (NH_4_)_2_SO_4_ only) at a given time point, t, and the percentage of NH_4_^+^-N loss in the treated sample ((NH_4_)_2_SO_4_ and NI) at the same time point, according to Eq. (3):

3 $$ {\text{nitrification}}\;{\text{inhibition}}\,(\% )\,based\,on\,NH_{4}^{ + } - N = \frac{{[{\text{NH}}_{4}^{ + } - {\text{N}}\,{\text{loss}}\,{\text{(\%)}}]_{{{\text{t}},{\text{control}}}} - [{\text{NH}}_{4}^{ + } - {\text{N}}\,{\text{loss}}\,{\text{(\%)}}]_{{{\text{t}},{\text{treated}}}} }}{{\left| {[{\text{NH}}_{4}^{ + } - {\text{N}}\,{\text{loss}}\,{\text{(\%)}}]_{{{\text{t}},{\text{control}}}} } \right|}} \times 100$$

We also calculated the nitrification inhibition (%) based on the accumulation rate of NO_x_^–^-N from the NO_x_^–^-N accumulation rates of the fertilised control (treatment with (NH_4_)_2_SO_4_ only) and the treated sample ((NH_4_)_2_SO_4_ and NI) after 28 days, according to Eq. (4):

4 $${\text{nitrification}}\,{\text{inhibition}}\,(\% )\,based\,on\,NO_{x}^{ - } - N = \frac{{[{\text{NO}}_{{\text{x}}}^{ - } - {\text{N}}\,{\text{accumulation}}]_{{{\text{control}}}} - [{\text{NO}}_{{\text{x}}}^{ - } - {\text{N}}\,{\text{accumulation}}]_{{{\text{treated}}}} }}{{\left| {[{\text{NO}}_{{\text{x}}}^{ - } - {\text{N}}\,{\text{accumulation}}]_{{{\text{control}}}} } \right|}} \times 100$$

It should be noted that negative inhibition values were obtained for some treatments, which could be due to a greater NH_4_^+^-N loss, such as immobilisation, at that time-point compared to the (NH_4_)_2_SO_4_-control treatment.

### Statistical analysis

All data presented are means of three replicates. Statistical analyses were performed on raw NH_4_^+^-N and NO_x_^–^-N data in R (version 3.5.2)^29^, using the statistical package *emmeans*^30^. Data were assessed for statistical significance (*P* < 0.05) via two-way analysis of variation (ANOVA)^31^ assessing the impact of the two factors “Day” and “Treatment”, and pair-wise comparisons between treatments at each time point were evaluated using a TukeyHSD post-hoc adjustment. Statistical results for inhibitor treatments compared to both the fertilised control (NH_4_)_2_SO_4_ treatment and DMPP treatment are included in Tables S4 and S5 in the SI, along with an example R script.

## Results and discussion

Initial screening of inhibitory activity in dependence of the chemical structure was performed through soil incubation tests of 1,2,3-triazoles N001–N012. These tests were conducted at 25 °C in both soils (at 10 mol% of the applied N) alongside control (NH_4_)_2_SO_4_ treatments and DMPP treatments (at 1.6 (L-DMPP) or 3.6 mol% (M-DMPP) of the applied N) to provide guidelines for the design and synthesis of further inhibitor compounds through an iterative process. As shown in Table 1, N001–N012 are di- and tri-substituted triazoles possessing a random combination of non-polar (R = *n*-C_4_H_9_, *n*-C_5_H_11_), polar aprotic (R = (CH_2_)_1,3_C(= O)OC_2_H_5_, CH_2_OC(= O)CH_3_, (CH_2_)_3_NPhth) and polar protic substituents (R = C(CH_3_)_2_OH, CH_2_OH, (CH_2_)_3_NH_2_), which would be expected to interact differently with soil components and microorganisms. The most promising compounds were subsequently tested against DMPP treatment at the same application rate (10 mol% of the applied N, H-DMPP), along with compounds N013–N017 from the second iteration. N013–N017 are disubstituted triazoles with either two non-polar substituents (N013, N016, N017), one non-polar and one polar aprotic substituent (N014) and one non-polar and one polar protic substituent (N015). The measured NH_4_^+^-N and NO_x_^–^-N concentrations (in mg kg^-1^ soil) for all soil treatments are compiled in Tables S4 and S5 in the SI. In the control (NH_4_)_2_SO_4_ treatments the applied fertiliser NH_4_^+^-N was completely consumed within the 28-day incubation period in most studies.

### Horsham soil (pH 8.8)

The measured NH_4_^+^-N and NO_x_^–^-N concentrations from the initial soil incubation tests of N001–N012 over the 28-day testing period in comparison with L-DMPP and M-DMPP, respectively, are provided in Table S4 in the SI. The NH_4_^+^-N loss calculated according to Eq. (1) is compiled in Table S6 (SI). In general, for all treatments a gradual decrease of NH_4_^+^-N or increase of NO_x_^–^-N concentrations, respectively, over time was found. However, as we were most interested in identifying inhibitors with the longest-lasting effects, our discussion will be largely focussed on assessing the performance of the various treatments at the 28-day timepoint.

Compounds N002, N005, N006 were most effective at retaining NH_4_^+^-N in this soil but did not outperform L- or M-DMPP treatments. The amount of NH_4_^+^-N loss after 28 days was 33% in the presence of inhibitor N002, whereas with N005 and N006 it was 50% and 46%, respectively. In all of these treatments a reduced NO_x_^–^-N kg^-1^ soil concentration was found after 28 days, compared with the (NH_4_)_2_SO_4_-fertilised treatment. The extent of NH_4_^+^-N loss was significantly higher for the other compounds, with N004, N007, N010 and N012 exhibiting practically quantitative loss of NH_4_^+^-N after 28 days.

**Figure 3:**
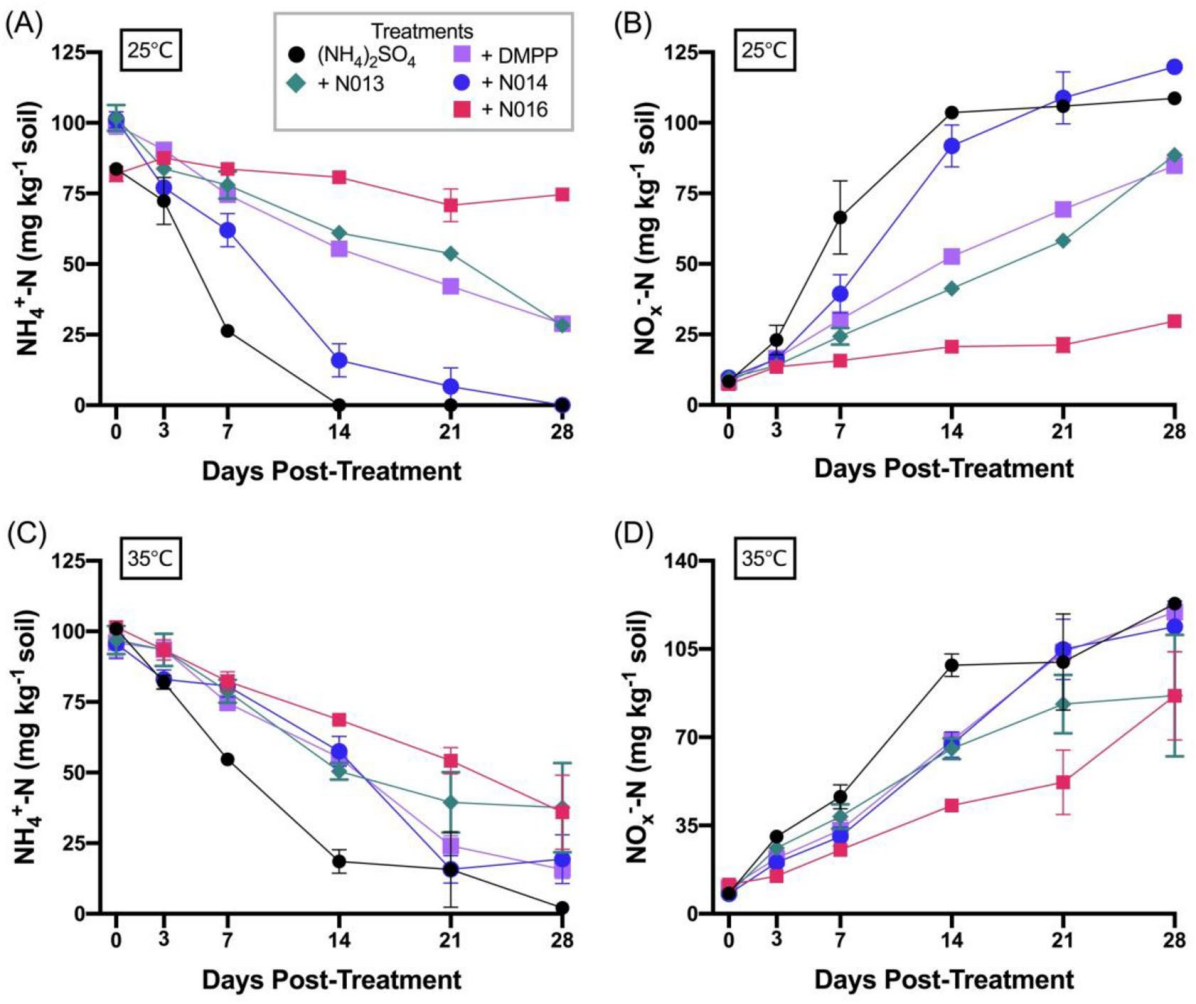
Measured NH_4_^+^-N (**A**, **C**) and NO_x_^–^-N (**B**, **D**) concentrations of Horsham soil incubated at 25 °C (**A**, **B**) and 35 °C (**C**, **D**) over 28 days following treatment with: (NH_4_)_2_SO_4_ [Black circle], (NH_4_)_2_SO_4_ + DMPP [Pink square], (NH_4_)_2_SO_4_ + N013 [ Green diamond], (NH_4_)_2_SO_4_ + N014 [Blue circle], (NH_4_)_2_SO_4_ + N016 [Violet square]. Inhibitors were applied at 10 mol% of fertiliser N. Data show the mean values (n = 3), errors are standard errors of the mean.

N002, the best performing inhibitor from the initial screening experiments was re-tested together with N013–N017, alongside DMPP at the same application rate. The measured NH_4_^+^-N and NO_x_^–^-N concentrations over the 4-week interval are shown in Fig. 3A,B exemplary for N013, N014 and N016, DMPP and the uninhibited control at 25 °C (see Table S4 in the SI for complete data).

Table 2 compiles the extent of NH_4_^+^-N loss for these treatments at selected time points (see Table S6 in SI for all data). Of all these inhibitors, compounds N013, N016, N017 outperformed the uninhibited control treatment at retaining NH_4_^+^ at 25 °C (*P* < 0.001) and inhibiting NO_x_^−^ formation (*P* = 0.013 (N013), 0.001 (N016), < 0.001 (N017)) after 28 days. Overall, however, DMPP performed better at both retaining NH_4_^+^ and inhibiting NO_x_^−^ formation (*P* < 0.001) than any of these compounds, which is reflected by only 9% NH_4_^+^-N loss after 28 days, compared to about 70% for N013, N016 and N017 (Table 2). It should be noted that in these soil testing experiments inhibitor N002 performed only reasonably well until day 7, before dropping in efficiency beyond this time point. The possible reason for the variable performance of this compound will be discussed below. Both compounds N014 and N015 had a low inhibitory activity in this soil, as revealed by the complete loss of NH_4_^+^-N and a high NO_x_^–^-N content after 28 days.

**Table 2:** Ammonium loss (%) during a 28-day incubation and NO_x_^–^-N production rate in Horsham soil (pH 8.8). All samples were treated with the fertiliser (NH_4_)_2_SO_4_ at a rate of 100 mg N kg^-1^.[^a^,^b^,^c^].

Treatment	NH_4_^+^-N loss/%			NO_x_^–^-N production rate^[d]^
	Day 7	Day 14	Day 28	
**(I) Incubations at 25 °C**				
(NH_4_)_2_SO_4_-control	68.5 ± 2.4	100 ± 2.7	100 ± 2.7	3.6 ± 0.1
DMPP	− 2.1 ± 3.2	1.4 ± 2.7	8.9 ± 3.2	0.8 ± 0.1
N002	17.9 ± 6.9	44.5 ± 7.2	90.4 ± 9.7	3.4 ± 0.1
N013	23.4 ± 5.4	40.0 ± 4.3	72.2 ± 4.8	2.8 ± 0.1
N014	38.7 ± 5.3	84.3 ± 5.6	100 ± 3.2	3.9 ± 0.1
N015	47.4 ± 2.9	88.9 ± 4.0	100 ± 3.6	3.8 ± 0.1
N016	24.9 ± 3.0	44.2 ± 3.3	70.9 ± 3.9	2.7 ± 0.1
N017	30.6 ± 5.5	35.1 ± 5.1	65.8 ± 5.2	2.5 ± 0.1
**(II) Incubations at 35 °C**				
(NH_4_)_2_SO_4_-control	45.9 ± 1.7	81.6 ± 3.7	98.0 ± 2.0	4.1 ± 0.1
DMPP	18.9 ± 2.8	32.3 ± 4.1	64.5 ± 12.7	2.7 ± 0.5
N002	8.8 ± 5.5	40.3 ± 5.4	71.6 ± 22.9	3.1 ± 0.8
N013	18.7 ± 5.5	48.0 ± 5.3	61.2 ± 14.2	2.8 ± 0.7
N014	15.7 ± 5.7	39.8 ± 6.6	79.7 ± 9.4	3.8 ± 0.3
N015	37.4 ± 5.4	77.0 ± 6.2	100 ± 6.3	4.3 ± 0.1
N016	22.3 ± 4.3	42.3 ± 4.6	83.7 ± 6.1	3.9 ± 0.1
N017	18.9 ± 2.8	32.3 ± 4.1	76.2 ± 2.1	3.7 ± 0.1

[^a^] Application rates are 10 mol% of applied fertiliser N. [^b^] Ammonium loss was calculated from NH_4_^+^-N concentrations detected in samples at each timepoint. [^c^] Mean values (n = 3); errors are standard errors of the mean. [^d^] After 28 days; in mg NO_x_^–^-N/kg soil/day.

Soil incubation studies were also performed at 35 °C to explore the inhibitory activity of N002 and N013–N017 in comparison with the commercial inhibitor at an elevated temperature. The measured concentrations of NH_4_^+^-N and NO_x_^–^-N for N013, N014 and N016 at selected time points are shown in Fig. 3C,D (for complete data see Table S4) and the amount of NH_4_^+^-N loss is included in Table 2 (the complete data set is included in Table S6). Clearly, at 35 °C the inhibitory activity of DMPP in this soil was significantly poorer compared with 25 °C. Of the compounds tested, N013 and DMPP performed better than the uninhibited control treatment both at retaining NH_4_^+^ (*P* = 0.004 (N013) and 0.008 (DMPP)) and retarding NO_x_^−^ production (*P* = 0.03 for both treatments), with N013 slowing down consumption of ammonium with comparable efficiency than DMPP (61% (N013) vs 65% (DMPP) NH_4_^+^-N loss on day 28, respectively).

Table 2 includes the calculated rates of NO_x_^–^-N production in the soil over the 28-day incubation period at 25 °C and 35 °C (see Table S7 for all data). Overall, incubation at 25 °C led to lower NO_x_^–^-N accumulation in all treatments compared with those at 35 °C, except for N002 and N014, where NO_x_^–^-N accumulation was lower at the elevated temperature. Treatment with DMPP at 25 °C resulted in the lowest accumulation rate (0.80 mg NO_x_^–^-N/kg soil/day), whereas the highest accumulation rate occurred in treatments with N014 at 25 °C and with N016 at 35 °C (3.9 mg NO_x_^–^-N/kg soil/day) for both treatments. The largest change in the accumulation rate going from 25 °C to 35 °C occurred in soil treated with DMPP, which increased by 1.9 mg NO_x_^–^-N/kg soil/day. The rate of NO_x_^–^-N accumulation in soil treated with N013 was the same at both temperatures (2.8 mg NO_x_^–^-N/kg soil/day).

**Figure 4:**
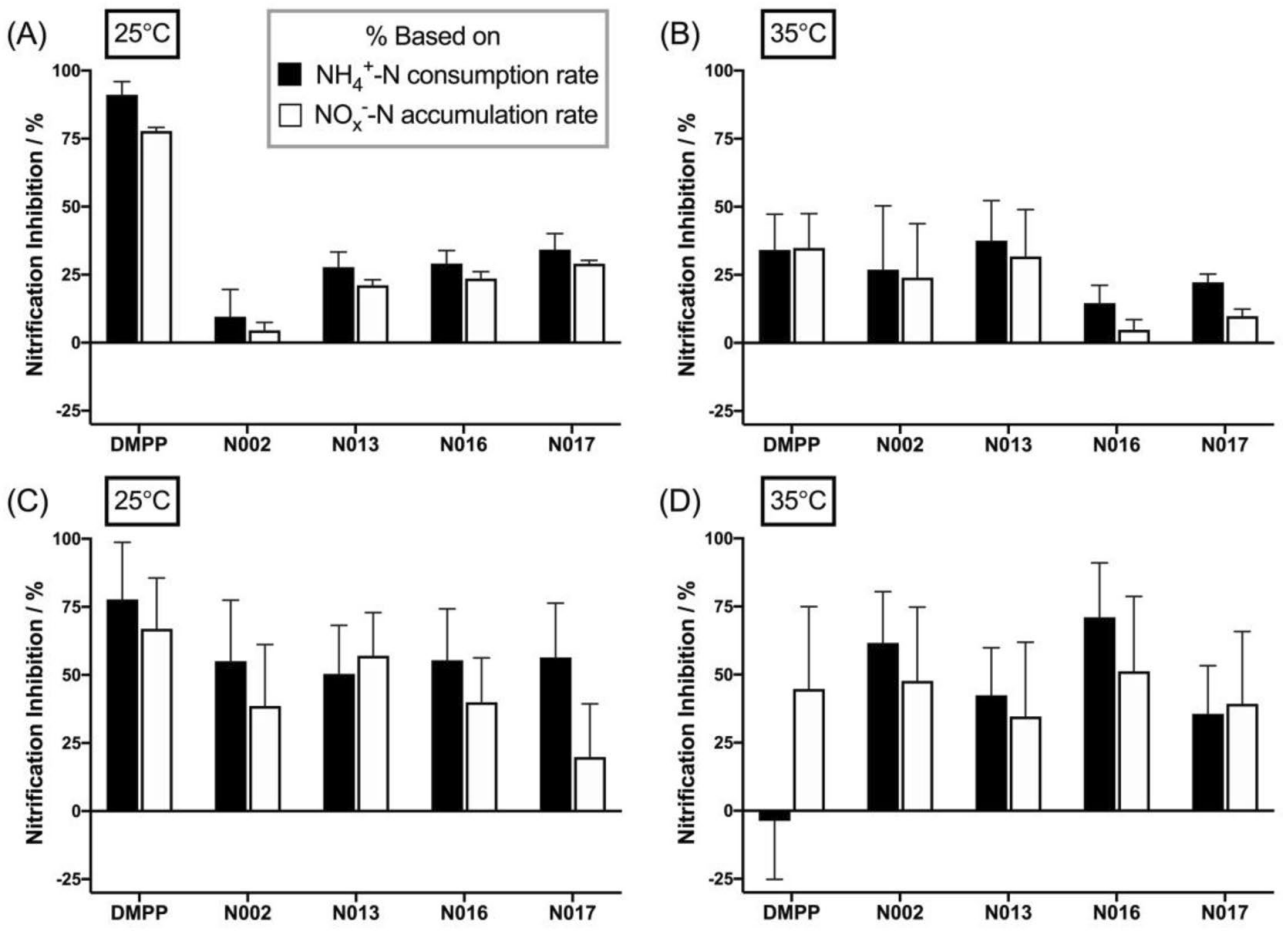
Nitrification inhibition (%) after 28-day incubation for selected inhibitor compounds; Horsham soil (pH 8.8) at (**A**) 25 °C and (**B**) 35 °C, Dahlen soil (pH 7.3) at (**C**) 25 °C and (**D**) 35 °C. Inhibition calculated from NH_4_^+^-N consumption rate (black filled bars) and from NO_x_^–^-N accumulation rate (white filled bars). All samples were treated with the fertiliser (NH_4_)_2_SO_4_ at a rate of 100 mg N kg^-1^. Inhibitor application rates are 10 mol% of applied fertiliser N for all treatments. Data show the mean values (n = 3), errors are standard errors of the mean. Full data is provided in Tables S10 and S11 in the SI.

The percentage of nitrification inhibition is shown in Fig. 4A,B for selected treatments on day 28 of sampling at 25 °C and at 35 °C.

The inhibition percentages were determined from either the extent of NH_4_^+^-N consumption according to Eq. (3), compared to the (NH_4_)_2_SO_4_-control treatment, or from the NO_x_^–^-N accumulation rates compared to the (NH_4_)_2_SO_4_-control treatment, according to Eq. (4). The full data set for all time points and treatments is shown in Table S10 in the SI.

Nitrification inhibition by N002, N013, N016 and N017 ranged from 10 to 35% at 25 °C (Fig. 4A). These compounds have linear C_3_–C_4_ alkyl substituents (N002, N013, N016) or a C_4_ alkynyl side chain (N017), respectively, suggesting that a low polarity could be beneficial for inhibitory activity in this alkaline soil. However, it should be noted that a variable performance of N002 in the Horsham soil was found with the amount of nitrified NH_4_^+^-N after 28 days between repeat soil incubations ranging from 33 to 90% (see Table 2 and Table S6, respectively). Compound N002 possesses two *n*-butyl substituents, whereas N013, N016 and N017 are substituted with *n*-propyl chains, which suggests that the lower efficiency of N002 (and potentially also its variable performance) might be due to its slightly higher lipophilicity. Lipophilicity is a crucial molecular property affecting inhibitory activity and is a key consideration in almost all drug (enzyme-inhibitor) discovery processes^32–34^. While further studies are clearly required, this hypothesis is supported by the finding that N001, which contains an *n*-butyl- and an *n*-pentyl substituent and should therefore be even more lipophilic than N002, does not exhibit noteworthy inhibitory activity after 28 days (Table S10).

In general, the inhibition percentages calculated based on NH_4_^+^-N loss were slightly higher than those based on NO_x_^–^-N accumulation in this soil (an average of 6.7% and 6.2% higher at 25 °C and 35 °C respectively, 6.6% higher overall, Fig. 4A,B). While not further explored here, this finding may indicate that either a small amount of NH_4_^+^ was formed in the soil through mineralisation or a small amount of NO_x_^−^ was produced via pathways other than autotrophic nitrification.

Inspection of the nitrification inhibition at 35 °C shown in Fig. 4B clearly confirmed the decreased effectiveness of DMPP in this soil at the higher temperature already seven days post-treatment, similar to previous observations in the literature^17^, whereas the activity of N002, N014 and N017 did not drop at the same rate in the first week after treatment (the data are included in Table S10). However, after 28 days only the N013 treatment exhibited similar or slightly better nitrification inhibition than DMPP.

### Dahlen soil (pH 7.3)

**Figure 5:**
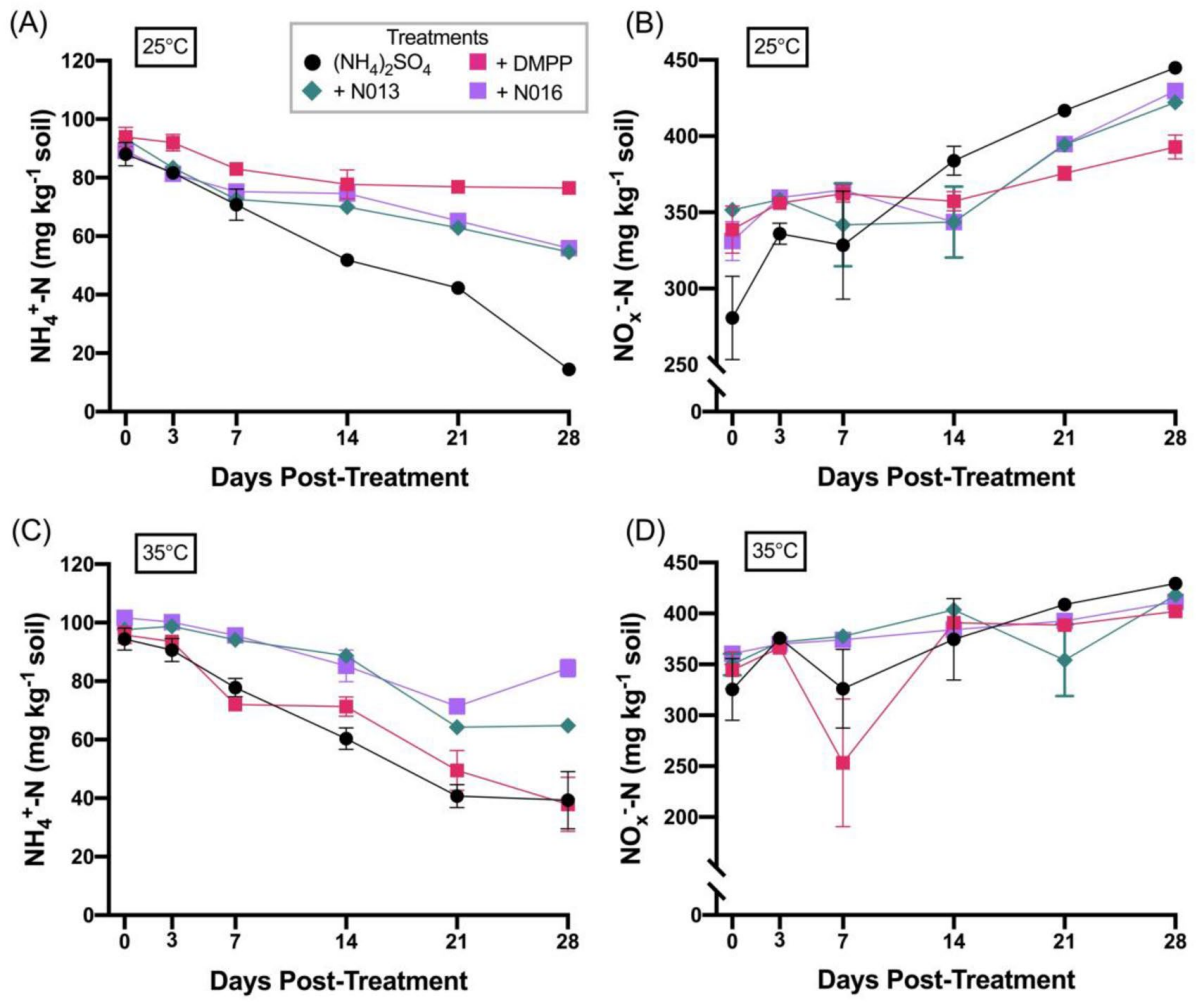
Measured NH_4_^+^-N (**A**, **C**) and NO_x_^–^-N (**B**, **D**) concentrations of Dahlen soil incubated at 25 °C (**A**, **B**) and 35 °C (**C**, **D**) over 28 days following treatment with: (NH_4_)_2_SO_4_ [Black circle], (NH_4_)_2_SO_4_ + DMPP [Pink square], (NH_4_)_2_SO_4_ + N013 [Green diamond], (NH_4_)_2_SO_4_ + N016 [Violet square]. Inhibitors were applied at 10 mol% of fertiliser N. Data show the mean values (n = 3), errors are standard errors of the mean.

The incubation studies in the Dahlen soil were performed with the same application rate of 100 mg N kg^-1^ soil as used in the Horsham soil. The measured NH_4_^+^-N and NO_x_^–^-N concentrations over the 4-week interval are shown in Fig. 5A,B exemplary for N013, N016, DMPP and the untreated control at 25 °C (the complete data are provided in Table S5). Overall, the most effective inhibitors at retaining NH_4_^+^-N were, apart from DMPP, compounds N002, N006, N013, N014, N016 and N017, which is reflected by a similar trend for their ability to inhibit NO_x_^–^-N formation.

Specifically, after the 28-day testing period, all of N002, N013, N016 and N017 performed better at retaining NH_4_^+^-N (*P* < 0.001) than the uninhibited control treatment and DMPP. It should be noted that the considerably large error for the NO_x_^–^-N measurements shown in Fig. 5B,D is likely due to the fact that the amount of NO_3_^-^ in this soil was considerably higher than in the Horsham soil prior to commencing testing (NO_3_^–^-N: 270 mg kg^-1^ (Dahlen) vs 7.2 mg kg^-1^ (Horsham); see Table S2).

The data for NH_4_^+^-N loss for selected triazoles, DMPP and the control experiment without inhibitor at 25 °C after 7, 14 and 28 days are compiled in Table 3. The complete data for all triazoles tested in this soil as well as for DMPP (at different rates) can be found in Table S8. Comparison of the control experiments performed with (NH_4_)_2_SO_4_ alone revealed that nitrification was generally slower in this soil than in the more alkaline Horsham soil at 25 °C. Microbiological analyses should provide further insight into the different behaviour of these two soils, but such studies were outside the scope of the present investigation.

**Table 3:** Ammonium loss (%) during a 28-day incubation and NO_x_^–^-N production rate in Dahlen soil (pH 7.3). All samples were treated with the fertiliser (NH_4_)_2_SO_4_ at a rate of 100 mg N kg^-1^.[^a^,^b^,^c^].

Treatment	NH_4_^+^-N loss/%			NO_x_^–^-N production rate^[d]^
	Day 7	Day 14	Day 28	
**(I) Incubations at 25 °C**				
(NH_4_)_2_SO_4_-control	19.6 ± 15.2	41.1 ± 10.0	83.6 ± 13.3	5.9 ± 0.8
DMPP	11.7 ± 6.7	17.3 ± 12.7	18.6 ± 4.9	1.9 ± 0.5
N002	21.2 ± 12.2	20.3 ± 21.6	37.6 ± 11.0	3.6 ± 1.0
N013	22.2 ± 50.2	24.9 ± 59.3	41.5 ± 1.2	2.5 ± 0.1
N016	15.6 ± 5.0	16.4 ± 28.7	37.3 ± 4.4	3.5 ± 0.4
N017	10.4 ± 3.6	12.1 ± 3.2	36.4 ± 6.8	4.7 ± 0.8
**(II) Incubations at 35 °C**				
(NH_4_)_2_SO_4_-control	17.5 ± 4.3	36.1 ± 4.7	58.3 ± 9.3	3.7 ± 0.9
DMPP	24.8 ± 2.7	25.6 ± 3.8	60.5 ± 8.4	2.1 ± 0.5
N002	-1.3 ± 1.9^[e]^	2.0 ± 2.4	22.4 ± 1.5	1.9 ± 0.1
N013	3.5 ± 1.3	9.2 ± 1.1	33.6 ± 1.3	2.4 ± 0.3
N016	6.0 ± 1.5	16.1 ± 4.5	16.9 ± 2.6	1.8 ± 0.1
N017	8.8 ± 1.9	17.7 ± 2.6	37.6 ± 3.1	2.3 ± 0.2

[^a^] Application rates are 10 mol% of applied fertiliser N. [^b^] Ammonium loss was calculated from NH_4_^+^-N concentrations detected in samples at each timepoint. [^c^] Mean values (n = 3); errors are standard errors of the mean. [^d^] After 28 days; in mg NO_x_^–^-N/kg soil/day. [^e^] Negative value could indicate that treatment may trigger priming effects in the soil (see text).

At 25 °C the amount of NH_4_^+^-N loss in the presence of N002, N013, N016 and N017 was comparable or even slightly less (N016 and N017) than with DMPP until day 14. After this time point, however, the efficiency decreased to reach around 37–42% NH_4_^+^-N loss at day 28 for these triazole inhibitors, compared with about 19% for DMPP.

Incubation studies were also performed for this soil at 35 °C with DMPP and N002, N013, N016 and N017. The measured concentrations of NH_4_^+^-N and NO_x_^–^-N for N013, N016, DMPP and control are shown in Fig. 5C,D (see Table S5 for all treatments), and the amount of NH_4_^+^-N loss is given in Table 3 for selected time points (see Table S8 for complete data). Similar to the results in the Horsham soil, DMPP performance in this soil was considerably poorer at the higher temperature. In fact, NH_4_^+^-N loss after 28 days was as high with DMPP as without inhibitor (ca. 60%). In contrast to this, all of N002, N013, N016 and N017 retained NH_4_^+^-N better (*P* < 0.001) than both DMPP and the control treatment after 28 days, with NH_4_^+^-N nitrification ranging from 17% (N016) to 38% (N017).

Table 3 also includes the calculated rates of NO_x_^–^-N accumulation in the soil over the 28-day incubation period for N002, N013, N016 and N017 in comparison with DMPP and the unfertilised control (see Table S9 for complete data). Thus, for all treatments incubation at 25 °C resulted in higher NO_x_^–^-N accumulation rates compared with those performed at 35 °C, except for DMPP. Treatment with N016 at 35 °C resulted in the lowest accumulation rate (1.8 mg NO_x_^–^-N/kg soil/day), whereas the highest accumulation rate in an inhibitor-treated soil occurred for N017 at 25 °C (4.7 mg NO_x_^–^-N/kg soil/day). Interestingly, the accumulation rate dropped to 2.4 mg NO_x_^–^-N/kg soil/day for N017 at 35 °C, which is the largest reduction in the accumulation rate for all inhibitors tested in this series. On the other hand, the rate of NO_x_^–^-N accumulation in soil treated with N013 was least affected by the temperature change (2.5 vs 2.4 mg NO_x_^–^-N/kg soil/day, at 25 °C and 35 °C, respectively), mirroring the seemingly temperature-independent behaviour that was observed in the Horsham soil for this inhibitor.

Figure 4C,D show the nitrification inhibition (as percentage) for selected treatments at 25 °C and at 35 °C, respectively, on day 28 of sampling in this soil. The complete data are compiled in Table S11 in the SI.

The best performing inhibitors in the Dahlen soil at 25 °C were N002, N013, N016 and N017, similar to the Horsham soil, but with lower efficiency than DMPP. Remarkably, at 35 °C nitrification inhibition by all of N002, N013, N016 and N017 outperformed that of DMPP across the entire testing period. These findings demonstrate that 1,2,3-triazoles with short alkyl side chains can be considered as an improvement over DMPP in this soil type at elevated temperatures. Somewhat surprisingly, the alkynyl side chain in N017, which was designed to act as potential additional reaction site for enzyme-catalysed oxidation^35^, did not provide an obvious additional benefit over the non-functionalised short alkyl substituents (the equivalent saturated alkyl substitution pattern is present in N013). On the other hand, nitrification inhibition by N005, N008–N011 and N015 was quite variable in the Dahlen soil, with some compounds performing poorly across the entire sampling period and others having a rapidly decreasing nitrification inhibition dropping to 0% after 28 days of incubation. These data show that triazoles with polar ester, amine, ether and alcohol substituents were also not efficient inhibitors in this soil.

Similar to the Horsham soil, the inhibition percentages calculated based on NH_4_^+^-N consumption were slightly higher in the Dahlen soil at 25 °C but not at 35 °C (average 9.3% higher at 25 °C and 2.1% lower at 35 °C, overall an average of 7.2% higher, Fig. 4C,D), suggesting that a small portion of the detected NH_4_^+^-N or NO_x_^–^-N may have arisen from other processes than autotrophic nitrification (see above).

## Conclusion

One major challenge facing the development of new nitrification inhibitors is the requirement for consistent effectiveness over an extended time period and at different soil and environmental conditions. This work is an important first step on the way to the development of more efficient nitrification inhibitor compounds. We hypothesised that three adjacent nitrogen atoms in a planar arrangement should increase the probability of successful interaction with the active site in AMO compared with DMPP. A library of 17 di- and tri-substituted 1,2,3-triazoles was therefore designed and synthesized and their performance as NIs explored through SAR studies in two Australian soils with different pH. It was found that inhibitors with short non-polar alkyl side chains, i.e., N002, N013, N016 and N017, performed similar or even better than DMPP in neutral and alkaline soils. Increasing the lipophilicity of the triazole by extending the side chains by one or two carbon atoms (as in the series N013 < N002 < N001) reduced the inhibitory activity considerably. Inclusion of more polar substituents, for example ester, ether, amine or hydroxyl groups had a detrimental effect on the inhibitory activity in these soils. Overall, the nitrification inhibition values suggest that N013 is the best inhibitor in this series with consistent performance across both soils and at two different temperatures.

A major advantage of 1,2,3-triazoles over the existing nitrification inhibitors lies in the fact that these compounds are synthetically accessible in few reaction steps with a large variety of substituents from readily available precursors through Cu-catalysed or thermal azide/alkyne click chemistry. This synthesis is highly atom-economic with nearly all atoms present in the precursor molecules ending up in the product. Ultimately, rather than having one inhibitor for all soil conditions, the 1,2,3-triazole approach could enable tuning of the inhibitor properties, for example polarity, hydrophilicity, lipophilicity, etc., to soil specifications, such as different pH as well as different soil temperatures. Scaling up of the synthetic procedure to ensure safe and efficient handling of potentially explosive azides for industrial applications could be achieved through continuous-flow technology, which enables in situ generation of the azide, safe reagent mixing, lower catalyst loading as well as inline separation and purification^36,37^. Furthermore, methods involving the reaction of hydrazones or diazo compounds with primary amines in the absence of azides have recently been developed, which provide opportunities to explore the inhibitory performance of substituted 1,2,3-triazoles with substitution pattern that cannot be readily accessed through alkyne/azide click chemistry^38–41^.

Further evaluation of this inhibitor class is ongoing, and results from those investigations, in conjunction with complementary microbiological, degradation and ecotoxicology studies in soils of different pH, as well as ^15^ N tracing and gas-monitoring techniques to explore N transformations in the soil as influenced by the inhibitor structure, will be published in due course.

## Supplementary Information

Supplementary Information.
