# Supplementary material for: Substituted 1,2,3-triazoles: a new class of nitrification inhibitors
Source: Sci Rep. 2021 Jul 22;11:14980. doi: 10.1038/s41598-021-94306-1 (PMC8298478; doi:10.1038/s41598-021-94306-1)
Supplement: Supplementary file 1 — Supplementary Information. [file 41598_2021_94306_MOESM1_ESM.pdf]

Supplementary Information

Substituted 1,2,3-Triazoles: A New Class of Nitrification Inhibitors

Bethany I. Taggert[a], Charlie Walker[b], Deli Chen[c], Uta Wille[a,*]

[a]School of Chemistry, Bio21 Institute, The University of Melbourne, 30 Flemington Road, Parkville VIC 3010, Australia.
[b]Incitec Pivot Ltd. PO Box 54, North Geelong, VIC 3215, Australia.
[c]School of Agriculture and Food, The University of Melbourne, Parkville VIC 3010, Australia.
Email: uwille@unimelb.edu.au

Table of Contents

1. ***Synthesis of Nitrification Inhibitors*** **S2**

2. ***Spectra of Inhibitors N001 – N017*** **S11**

3. ***Soil Incubation Studies*** **S28**

4. ***Example R Script*** **S45**

5. ***References*** **S45**

1. Synthesis of Nitrification Inhibitors

Table S1. Overview of structures, synthetic approach and yields for the inhibitors N001-N017 studied in this work.

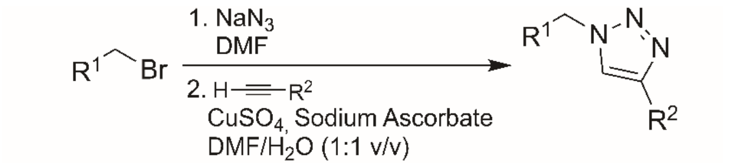

NI	R^1	R^2	Yield (%)
N001	$(CH_2)_2CH_3$	$(CH_2)_4CH_3$	64
N002	$(CH_2)_2CH_3$	$(CH_2)_3CH_3$	75
N003	$C(O)OCH_2CH_3$	$(CH_2)_3CH_3$	56
N004	$(CH_2)_2CH_3$	$C(CH_3)_2OH$	75
N005	$(CH_2)_2C(O)OCH_2CH_3$	$(CH_2)_3CH_3$	56
N006[a]	$(CH_2)_3NH_2$	$(CH_2)_3CH_3$	65
N013	$(CH_2)_2CH_3$	$(CH_2)_2CH_3$	89
N014[a]	$(CH_2)_2OCH_3$	$(CH_2)_3CH_3$	65
N015	$(CH_2)_2OH$	$(CH_2)_3CH_3$	36
N016	CH_2CH_3	$(CH_2)_2CH_3$	26
N017	$CH_2C{\equiv}CH$	$(CH_2)_2CH_3$	18

[a]Synthesis involved more steps than illustrated.

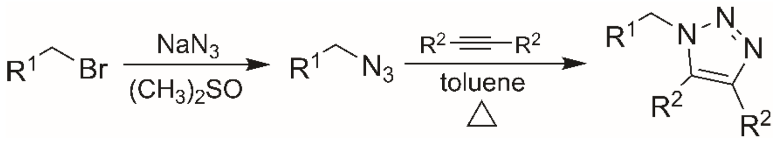

NI	R^1	R^2	Yield (%)
N007	$(CH_2)_3$NPhth	CH_2OH	54
N008	$(CH_2)_3$NPhth	$CH_2OC(O)CH_3$	71
N009	$(CH_2)_2C(O)OCH_2CH_3$	CH_2OH	33
N010	$(CH_2)_2C(O)OCH_2CH_3$	$CH_2OC(O)CH_3$	74
N011	$C(O)OCH_2CH_3$	$CH_2OC(O)CH_3$	63
N012	$C(O)OCH_2CH_3$	CH_2OH	33

1.1 General

Reaction progress was monitored by thin-layer chromatography (TLC) using silica gel 60 aluminium-backed plates coated with fluorescent indicator F254 (Merck). Plates were visualised using UV irradiation (254 nm) alone or in conjunction with ninhydrin-, potassium permanganate- or iodine-based stains. Purification by silica gel chromatography was performed using Davisil Chromatographic Silica Media LC60A 40-63 micron, with solvent systems as specified. All ^{1}H and ^{13}C NMR spectra were recorded on a 400 MHz Varian INOVA spectrometer (at 400 or 101 MHz, respectively) using solvent resonances as the internal standard (^{1}H NMR: $CDCl_3$ at 7.26 ppm, DMSO-d_6 at 2.50 ppm; ^{13}C NMR: $CDCl_3$ at 77.0 ppm, DMSO-d_6 at 39.5 ppm). Chemical shifts are reported in parts per million (ppm, δ), with the splitting patterns indicated as follows: s, singlet; d, doublet; t, triplet; q, quartet; p, pentet; h, hextet; m, multiplet; dd, doublet of doublets. The coupling constants, *J*, are reported in Hertz (Hz). Electrospray ionization high resolution mass spectrometry (HRMS) was performed on a Thermo Scientific Exactive Plus Orbitrap mass spectrometer (Thermo, Bremen, German) operated in positive mode.

1.2 General Procedure A. Synthesis of 1,4-disubstituted triazoles through copper(I)-catalysed azide-alkyne cycloaddition (CuAAC).

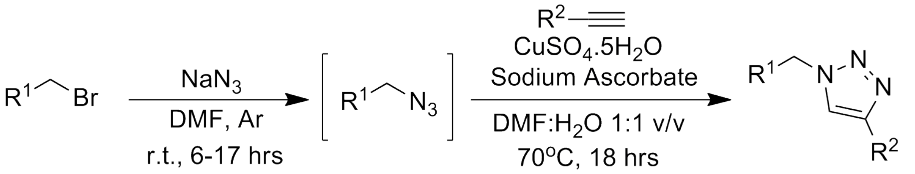

Figure S1. Reaction scheme for General Procedure A.

Sodium azide (1.2 or 1.5 equiv.) was suspended in DMF (0.85 M) in a flask under argon atmosphere, and to this the appropriate alkyl bromide (1 equiv.) was added. The solution was stirred at room temperature for 6-17 hours. The reaction was quenched by the addition of H_2O (DMF/H_2O, 1:1 v/v), before the successive additions of $CuSO_4.5H_2O$ (0.06 equiv.), sodium ascorbate (0.3 equiv.) and the appropriate alkyne (1.2 or 1.5 equiv.). The reaction was heated at 70°C overnight with vigorous stirring. The reaction was cooled to room temperature before dilution with H_2O (at least 3 x DMF volume) and extraction with ethyl acetate. The extracts were combined, washed with 5% aq. LiCl solution and concentrated before purification by silica chromatography.

1.3 General Procedure B. Synthesis of 1,4,5-trisubstituted triazoles through thermal Huisgen 1,3-dipolar cycloaddition.

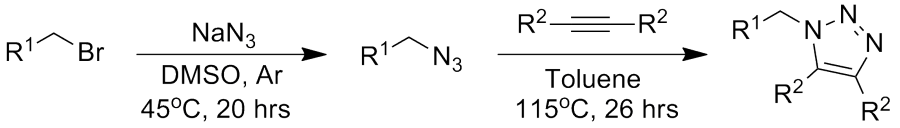

Figure S2. Reaction scheme for General Procedure B.

Sodium azide (1.5 equiv.) and appropriate alkyl bromide (1 equiv.) were charged into a flask flushed with argon. They were suspended in DMSO (1.28 M) and warmed to 45°C with vigorous stirring. The reaction was cooled to room temperature after 20 hours and quenched with H_2O (DMF/H_2O, 4:5 v/v), before extraction with ether. The ethereal extracts were concentrated under N_2 flow to an oil, which was used directly in the subsequent step. *CAUTION: Organic azides may be explosive, do not evaporate to dryness. Smaller azides were handled using solvent substitution, where toluene was added before ether was evaporated under N_2 flow.

The crude azide was suspended in toluene (0.21 M) before addition of the appropriate internal alkyne (1.1 equiv.). The reaction was then heated at 115°C with vigorous stirring. Once completed by TLC (24 to 48 hrs), the reaction was cooled. Toluene was removed *in vacuo* to leave crude triazole as a waxy brown solid. Purification of the crude product was achieved through recrystallisation or column chromatography.

1.4 Synthesis of 1-butyl-4-pentyl-1H-1,2,3-triazole (N001)

Synthesised from General Procedure A; N001 (2.14 g, 11.0 mmol, 64%) was obtained starting from sodium azide (25.6 mmol), 1-bromobutane (17.1 mmol), $CuSO_4.5H_2O$ (1.0 mmol), sodium ascorbate (5.1 mmol) and 1-heptyne (25.5 mmol). The crude mixture was purified by silica chromatography (Pet. Ether/EtOAc, 4:1; R_f = 0.27) to give N001 as colourless liquid (64%). ^{1}H NMR (400 MHz, $CDCl_3$): δ (ppm) 7.22 (s, 1H), 4.26 (t, J = 7.3 Hz, 2H), 2.69 – 2.60 (m, 2H), 1.82 (p, *J* = 7.4 Hz, 2H), 1.61 (p, *J* = 7.4 Hz, 2H), 1.37 – 1.21 (m, 6H), 0.89 (t, *J* = 7.4 Hz, 3H), 0.88 – 0.80 (m, 3H). ^{13}C NMR (101 MHz, $CDCl_3$): δ (ppm) 148.30, 120.32, 49.78, 32.27, 31.40, 29.14, 25.61, 22.35, 19.66, 13.93, 13.40. HRMS (ESI +) *m/z*: $[C_{11}H_{21}N_3 + H]^+$ calculated 196.18082, found 196.18098.

1.5 Synthesis of 1,4-butyl-1H-1,2,3-triazole (N002)

Synthesised from General Procedure A**;** N002 (2.33 g, 12.9 mmol, 75%) was obtained starting from sodium azide (25.6 mmol), 1-bromobutane (17.1 mmol), $CuSO_4.5H_2O$ (1.0 mmol), sodium ascorbate (5.1 mmol) and 1-hexyne (25.5 mmol). The crude mixture was purified by silica chromatography (Pet. Ether/EtOAc, 4:1; R_f = 0.19) to give N002 as colourless liquid (75%). ^{1}H NMR (400 MHz, $CDCl_3$): δ (ppm) 7.24 (s, 1H), 4.28 (t, *J* = 7.2 Hz, 2H), 2.68 (t, *J* = 7.7 Hz, 2H), 1.84 (p, *J* = 7.3 Hz, 2H), 1.62 (p, *J* = 7.5 Hz, 2H), 1.42 – 1.25 (m, 4H), 0.96 – 0.86 (m,

6H). ^{13}C NMR (101 MHz, $CDCl_3$): δ (ppm) 148.26, 120.35, 49.85, 32.28, 31.56, 25.31, 22.28, 19.69, 13.78, 13.43. HRMS (ESI +) *m/z*: $[C_{10}H_{19}N_3 + H]^+$ calculated 182.16517, found 182.16539.

1.6. Synthesis of 4-butyl-1H-1,2,3-triazole-1-acetic acid ethyl ester (N003)

Synthesised from General Procedure A; N003 (1.99 g, 9.44 mmol, 56%) was obtained starting from sodium azide (25.5 mmol), ethyl bromoacetate (17.0 mmol), $CuSO_4.5H_2O$ (1.0 mmol), sodium ascorbate (6.0 mmol) and 1-hexyne (25.5 mmol). The crude mixture was purified by silica chromatography (Pet. Ether/EtOAc, 4:1; R_f = 0.15) to give N003 as white solid (56%). ^{1}H NMR (400 MHz, $CDCl_3$): δ (ppm) 7.42 (s, 1H), 5.12 (s, 2H), 4.25 (q, *J* = 7.1 Hz, 2H), 2.74 (t, *J* = 7.7 Hz, 2H), 1.67 (p, *J* = 7.6 Hz, 2H), 1.38 (h, *J* = 7.3 Hz, 2H), 1.29 (t, *J* = 7.1 Hz, 3H), 0.92 (t, *J* = 7.4 Hz, 3H). ^{13}C NMR (101 MHz, $CDCl_3$): δ (ppm) 166.40, 148.75, 122.02, 62.31, 50.82, 31.38, 25.23, 22.23, 14.03, 13.78. HRMS (ESI +) *m/z*: $[C_{10}H_{17}N_3O_2 + H]^+$ calculated 212.13935, found 212.13977.

1.7 Synthesis of 1-butyl-4-(α,α-dimethyl methanol)-1H-1,2,3-triazole (N004)

Synthesised from General Procedure A; N004 (3.12 g, 17.0 mmol, 100%) was obtained starting from sodium azide (25.4 mmol), 1-bromobutane (17.0 mmol), $CuSO_4.5H_2O$ (1.0 mmol), sodium ascorbate (5.8 mmol) and 2-methyl-3-butyne-2-ol (25.5 mmol). The crude mixture was purified by silica chromatography (Pet. Ether/EtOAc, 1:1; R_f = 0.22) to give N006 as yellow liquid (quant.). ^{1}H NMR (400 MHz, $CDCl_3$): δ (ppm) 7.43 (s, 1H), 4.31 (t, *J* = 7.3 Hz, 2H), 2.77 (s, 1H), 1.87 (p, *J* = 7.4 Hz, 2H), 1.62 (s, 6H), 1.35 (h, *J* = 7.4 Hz, 2H), 0.94 (t, *J* = 7.4 Hz, 3H). ^{13}C NMR (101 MHz, $CDCl_3$): δ (ppm) 155.49, 118.90, 68.46, 50.04, 32.24, 30.46, 19.72, 13.43. HRMS (ESI +) *m/z*: $[C_9H_{17}N_3O + H]^+$ calculated 184.14444, found 184.14458.

1.8 Synthesis of 4-butyl-1H-1,2,3-triazole-1-butanoic acid ethyl ester (N005)

Synthesised from General Procedure A; N005 (1.08 g, 4.5 mmol, 56%) was obtained starting from sodium azide (8.0 mmol), ethyl 4-bromobutyrate (8.4 mmol), $CuSO_4.5H_2O$ (0.7 mmol), sodium ascorbate (4 mmol) and 1-hexyne (8.0 mmol). The crude mixture was purified by silica chromatography (Pet. Ether/EtOAc, 3:1; R_f = 0.24) to give N005 as pale yellow oil (56%). ^{1}H NMR (400 MHz, $CDCl_3$): δ (ppm) 7.24 (s, 1H), 4.34 (t, *J* = 6.9 Hz, 2H), 4.08 (q, *J* = 7.1 Hz, 2H), 2.65 (t, *J* = 7.7 Hz, 2H), 2.28 (t, *J* = 7.1 Hz, 2H), 2.15 (p, *J* = 6.9 Hz, 2H), 1.59 (p, *J* = 7.6 Hz, 2H), 1.33 (h, *J* = 7.4 Hz, 2H), 1.20 (t, *J* = 7.1 Hz, 3H), 0.87 (t, *J* = 7.4 Hz, 3H). ^{13}C NMR (101 MHz, $CDCl_3$): δ (ppm) 172.32, 148.43, 120.62, 60.60, 48.95, 31.50, 30.70, 25.46, 25.28, 22.24, 14.12, 13.75. HRMS (ESI +) *m/z*: $[C_{12}H_{21}O_2N_3 + H]^+$ calculated 240.17065, found 240.17061.

1.9 Synthesis of 4-butyl-1H-1,2,3-triazole-1-propanamine

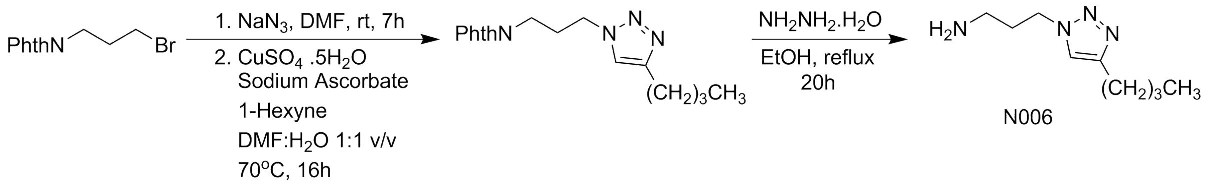

Figure S3. Reaction scheme for the synthesis of N006.

a) <u>2-[3-(4-Butyl-1H-1,2,3-triazol-1-yl)propyl]-1H-isoindole-1,3(2H)-dione:</u> Synthesised from modified reported procedures (Pyta, K.; et al., *European Journal of Medicinal Chemistry* 2014, *84*, 651; and Wang, Y.-F.; et al., *Organic Letters* 2013, *15* (11), 2842). *N*-(3-Bromopropyl)phthalimide (10.1 mmol) and sodium azide (15.2 mmol) were dissolved in DMF (26 mL) under argon and stirred at room temperature for 7 hrs. The reaction was diluted with H_2O (26 mL), followed by addition of $CuSO_4.5H_2O$ (1.0 mmol), sodium ascorbate (5.3 mmol) and 1-hexyne (25.5 mmol) in succession. The reaction was heated at 70°C with vigorous stirring.

The reaction was cooled to room temperature after 16 hours, diluted with H_2O (80 mL) and extracted with ethyl acetate (3 x 80 mL). The combined extracts were concentrated and purified by silica chromatography (Pet. Ether/EtOAc, 2:3; R_f = 0.33). If crude failed to solidify due to remaining DMF, the sample was treated with 5% aq. LiCl solution to cause precipitation of 2-[3-(4-butyl-1H-1,2,3-triazol-1-yl)propyl]-1H-isoindole-1,3(2H)-dione as a cream powder (84%). ^{1}H NMR (400 MHz, $CDCl_3$): δ (ppm) 7.89 – 7.80 (m, 2H), 7.78 – 7.68 (m, 2H), 7.44 (s, 1H), 4.36 (t, *J* = 6.9 Hz, 2H), 3.74 (t, *J* = 6.5 Hz, 2H), 2.68 (t, *J* = 7.7 Hz, 2H), 2.31 (p, *J* = 6.8 Hz, 2H), 1.63 (p, *J* = 7.4 Hz, 2H), 1.37 (h, *J* = 7.4 Hz, 2H), 0.92 (t, *J* = 7.4 Hz, 3H). ^{13}C NMR (101 MHz, $CDCl_3$): δ (ppm) 168.29, 148.41, 134.17, 131.87, 123.36, 121.00, 47.58, 35.11, 31.53, 29.44, 25.33, 22.30, 13.81. HRMS (ESI +) *m/z*: $[C_{17}H_{20}N_4O_2 +H]^+$ calculated 313.16590, found 313.16592.

b) <u>N006:</u> 2-[3-(4-Butyl-1H-1,2,3-triazol-1-l)propyl]-1H-isoindole-1,3(2H)-dione (8.5 mmol) from the previous step was dissolved in ethanol (0.06 M) before being treated with hydrazine monohydrate (12.67 mmol). The solution was stirred vigorously and heated to 90°C. After heating overnight, a white precipitate had formed. The reaction was cooled, and the precipitate was removed by filtration and washed thoroughly. The filtrate was concentrated, and the resulting solid was resuspended in CH_2Cl_2 and filtered again. The filtrate was concentrated to a yellow oil which was purified by silica chromatography (CH_2Cl_2/MeOH/30% aq. NH_3, 10:1:0.1; R_f = 0.16) to give N006 as a cream solid (1.01 g, 5.5 mmol, 65%). ^{1}H NMR (400 MHz, DMSO-d_6): δ (ppm) 7.80 (s, 1H), 4.33 (t, *J* = 7.0 Hz, 2H), 2.57 (t, *J* = 7.6 Hz, 2H), 2.47 (t, *J* = 6.6 Hz, 2H), 1.82 (p, *J* = 6.8 Hz, 2H), 1.64 – 1.44 (m, 4H), 1.29 (h, *J* = 7.3 Hz, 2H), 0.87 (t, *J* = 7.4 Hz, 3H). ^{13}C NMR (101 MHz, DMSO-d_6): δ (ppm) 147.17, 122.06, 47.40, 38.88, 34.08,

31.60, 25.14, 22.12, 14.10. HRMS (ESI +) *m/z*: $[C_9H_{18}N_4 + H]^+$ calculated 183.16042, found 183.16057.

1.11 Synthesis of 2-[3-[4,5-di(hydroxymethyl)-1H-1,2,3-triazole]propyl]-isoindoline-1,3-dione (N007)

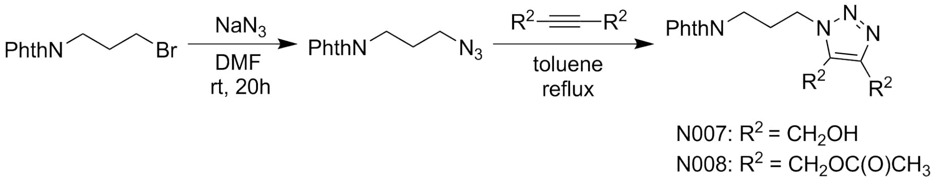

Figure S4. Reaction scheme for the synthesis of triazoles N007 and N008.

a) *N*-(3-azidopropyl)phthalimide: Sodium azide (9.4 mmol) was suspended in DMF (26 mL) under argon, and to this solution *N*-(3-bromopropyl)phthalimide (8.7 mmol) was added. The mixture was stirred at room temperature overnight. The reaction was then slowly diluted with H_2O (100 mL) and extracted with ether. Concentration of the ethereal extracts provided *N*-(3-azidopropyl)phthalimide as a waxy cream solid (1.83 g, 7.94 mmol, 92%). ^1H NMR (400 MHz, $CDCl_3$): δ (ppm) 7.90 – 7.79 (m, 2H), 7.77 – 7.67 (m, 2H), 3.78 (t, *J* = 6.8 Hz, 2H), 3.38 (t, *J* = 6.7 Hz, 2H), 1.96 (p, *J* = 6.8 Hz, 2H). ^{13}C NMR (101 MHz, $CDCl_3$): δ (ppm) 168.25, 134.04, 132.00, 123.31, 49.04, 35.38, 28.03. HRMS (ESI +) *m/z*: $[C_{11}H_{10}O_2N_4 + H]^+$ calculated 231.08765, found 231.08771.

b) N007: *N*-(3-azidopropyl)phthalimide (7.9 mmol) from the previous step was suspended in toluene (0.2 M), followed by addition of 2-butyne-1,4-diol (8.7 mmol). The reaction was stirred vigorously and heated to 115°C for 41 hrs. Toluene was evaporated, and the crude solid was recrystallised from H_2O to give **7** as a white powder (1.34 g, 4.3 mmol, 54%). ^1H NMR (400 MHz, DMSO-d_6): δ (ppm) 7.89 – 7.77 (m, 4H), 5.29 (t, *J* = 5.4 Hz, 1H), 5.01 (t, *J* = 5.6 Hz, 1H), 4.57 (d, *J* = 5.3 Hz, 2H), 4.46 (d, *J* = 5.5 Hz, 2H), 4.37 (t, *J* = 7.3 Hz, 2H), 3.65 (t, *J* = 7.0 Hz, 2H), 2.18 (p, *J* = 7.2 Hz, 2H). ^{13}C NMR (101 MHz, DMSO-d_6): δ (ppm) 168.36, 145.05, 134.76, 134.52, 132.16, 123.44, 54.63, 51.10, 45.99, 35.69, 28.88. HRMS (ESI +) *m/z*: $[C_{15}H_{16}O_4N_4 + H]^+$ calculated 317.12443, found 317.12448.

1.12 Synthesis of 2-[3-[4,5-(methyl ethanoate)-1H-1,2,3-triazole]propyl]-isoindoline-1,3-dione (N008)

Using the procedure described for N007 with 2-butyne-1,4-diol diacetate as alkyne, the crude product was recrystallised from ethanol to give N008 as white crystals (3.69 g, 9.23 mmol, 71%). ^1H NMR (400 MHz, $CDCl_3$): δ (ppm) 7.89 – 7.80 (m, 2H), 7.78 – 7.69 (m, 2H), 5.23 (s, 2H), 5.22 (s, 2H), 4.46 – 4.37 (m, 2H), 3.82 (t, *J* = 6.7 Hz, 2H), 2.36 (p, *J* = 6.9 Hz, 2H), 2.06 (s, 3H), 2.04 (s, 3H). ^{13}C NMR (101 MHz, $CDCl_3$): δ (ppm) 170.67, 170.02, 168.20, 142.09, 134.17,

131.89, 130.65, 123.36, 56.67, 52.64, 46.51, 35.23, 29.09, 20.82, 20.50. HRMS (ESI +) *m/z*: $[C_{19}H_{20}N_4O_6 + H]^+$ calculated 401.14556, found 401.14563.

1.13 Synthesis of ethyl 4,5-bis(hydroxymethyl)-1H-1,2,3-triazole-1-butyrate (N009)

Synthesised from modified General Procedure B, using sodium azide (22 mmol) and ethyl 4-bromobutyrate (19 mmol) and heating in DMF (20 mL). The crude azide was the reacted with 2-butyne-1,4-diol (14 mmol) in toluene at 115°C for 24 hrs. The crude mixture was purified by silica chromatography (CH_2Cl_2/CH_3OH, 10:0.6; R_f = 0.28) to give N009 as pale yellow oil (1.21 g, 4.6 mmol, 33%). ^1H NMR (400 MHz, $CDCl_3$): δ (ppm) 4.71 (s, 2H), 4.65 (s, 2H), 4.41 (t, *J* = 7.1 Hz, 2H), 4.38 (s, 1H), 4.33 (s, 1H), 4.10 (q, *J* = 7.1 Hz, 2H), 2.36 (t, *J* = 7.0 Hz, 2H), 2.21 (p, *J* = 7.1 Hz, 2H), 1.23 (t, *J* = 7.1 Hz, 3H).^{13}C NMR (101 MHz, $CDCl_3$): δ (ppm) 172.70, 144.63, 134.35, 60.82, 55.21, 51.94, 47.65, 30.75, 24.99, 14.14. HRMS (ESI +) *m/z*: $[C_{10}H_{17}N_3O_4 + H]^+$ calculated 244.12918, found 244.12921.

1.14. Synthesis of ethyl 4,5-bis(methyl ethanoate)-1H-1,2,3-triazole-1-butyrate (N010)

Synthesised from General Procedure B; N010 (2.5 g, 7.7 mmol, 74%, colourless oil) was obtained from sodium azide (15.8 mmol), ethyl 4-bromobutyrate (10.5 mmol) and 2-butyne-1,4-diol (11.1 mmol). The crude mixture was purified by silica chromatography (Pet. Ether/EtOAc, 3:2; R_f = 0.1). ^1H NMR (400 MHz, $CDCl_3$): δ (ppm) 5.24 (s, 2H), 5.23 (s, 2H), 4.42 (t, *J* = 7.1 Hz, 2H), 4.11 (q, *J* = 7.1 Hz, 2H), 2.39 (t, *J* = 7.0 Hz, 2H), 2.22 (p, *J* = 7.1 Hz, 2H), 2.06 (s, 3H), 2.05 (s, 3H), 1.24 (t, *J* = 7.1 Hz, 3H). ^{13}C NMR (101 MHz, $CDCl_3$): δ (ppm) 172.24, 170.65, 170.01, 142.06, 130.64, 60.71, 56.71, 52.69, 47.65, 30.73, 25.15, 20.80, 20.56, 14.15. HRMS (ESI +) *m/z*: $[C_{14}H_{21}N_3O_6 + H]^+$ calculated 328.15031, found 328.15021.

1.15 Synthesis of ethyl 4,5-bis(methyl ethanoate)-1H-1,2,3-triazole-1-acetate (N011)

Synthesised from General Procedure B; N010 (1.9 g, 6.3 mmol, 63%, pale yellow oil) was obtained from sodium azide (15.1 mmol), ethyl-2-bromoacetate (10.0 mmol) and 2-butyne-1,4-diol diacetate (10.7 mmol). The crude mixture was purified by silica chromatography (Pet. Ether/EtOAc, 1:1; R_f = 0.43). ^1H NMR (400 MHz, $CDCl_3$): δ (ppm) 5.25 (s, 2H), 5.24 (s, 2H), 5.23 (s, 2H), 4.24 (q, *J* = 7.1 Hz, 2H), 2.06 (s, 3H), 2.03 (s, 3H), 1.29 (t, *J* = 7.1 Hz, 3H). ^{13}C NMR (101 MHz, $CDCl_3$): δ (ppm) 170.68, 170.16, 166.23, 142.21, 131.79, 62.50, 56.60, 52.97, 49.78, 20.81, 20.48, 14.04. HRMS (ESI +) *m/z*: $[C_{12}H_{17}N_3O_6 + H]^+$ calculated 300.11901, found 300.11890.

1.16. Synthesis of ethyl 4,5-bis(hydroxymethyl)-1H-1,2,3-triazole-1-acetate (N012)

Synthesised according to: Wen, Y.-n.; et al., *Nucleosides, Nucleotides and Nucleic Acids* 2016, *35*(3), 147. N012 (0.85 g, 3.9 mmol, 33%, white solid) was obtained from sodium azide (14.3 mmol), ethyl-2-bromoacetate (13.5 mmol) and 2-butyne-1,4-diol (12.1 mmol). The crude mixture was purified by silica chromatography (CH_2Cl_2/CH_3OH, 10:1; R_f = 0.13). ^1H NMR (400

MHz, DMSO-d6): δ (ppm) 5.34 (t, *J* = 5.5 Hz, 1H), 5.31 (s, 2H), 5.08 (t, *J* = 5.7 Hz, 1H), 4.57 (d, *J* = 5.4 Hz, 2H), 4.50 (d, *J* = 5.5 Hz, 2H), 4.15 (q, *J* = 7.1 Hz, 2H), 1.20 (t, *J* = 7.1 Hz, 3H). ^{13}C NMR (101 MHz, DMSO-d6): δ (ppm) 167.58, 144.81, 135.15, 61.82, 54.61, 51.67, 49.70, 14.40. HRMS (ESI +) *m/z*: $[C_8H_{13}N_3O_4 + H]^+$ calculated 216.09788, found 216.09734.

1.17. Synthesis of 1-butyl-4-propyl-1H-1,2,3-triazole (N013)

Synthesised from General Procedure A; N013 (2.55 g, 15.2 mmol, 89%, colourless oil) was obtained from sodium azide (20.3 mmol), 1-bromobutane (17.0 mmol), $CuSO_4.5H_2O$ (1.0 mmol), sodium ascorbate (5.2 mmol) and 1-pentyne (20.0 mmol). The crude mixture was purified by silica chromatography (Pet. Ether/EtOAc, 3:2; R_f = 0.37). ^1H NMR (400 MHz, $CDCl_3$): δ (ppm) 7.23 (s, 1H), 4.28 (t, *J* = 7.2 Hz, 2H), 2.66 (t, *J* = 7.6 Hz, 2H), 1.84 (p, *J* = 7.3 Hz, 2H), 1.66 (h, *J* = 7.4 Hz, 2H), 1.32 (h, *J* = 7.4 Hz, 2H), 0.96 – 0.89 (m, 6H). ^{13}C NMR (101 MHz, $CDCl_3$): δ (ppm) 148.11, 120.38, 49.82, 32.29, 27.66, 22.70, 19.68, 13.74, 13.43. HRMS (ESI +) *m/z*: $[C_9H_{17}N_3 + H]^+$ calculated 168.14952, found 168.14951.

1.18 Synthesis of 1-(2-methoxyethyl)-4-butyl-1H-1,2,3-triazole (N014)

N014 was obtained from purified N015 (6.3 mmol), which was dissolved in anhydrous THF (42 mL) under argon and cooled to 0°C. NaH (6.6 mmol) was added in a single portion. Once gas evolution had ceased, MeI (9.5 mmol) was added in three portions, and the mixture stirred at room temperature for 24 hours. The reaction was diluted with H_2O and THF was removed *in vacuo*. The product was extracted into ethyl acetate and concentrated. The crude mixture was purified by silica chromatography (Pet. Ether/EtOAc, 1:1; R_f = 0.21) to give N014 as colourless oil (0.75 g, 4.1 mmol, 65%). ^1H NMR (400 MHz, $CDCl_3$): δ (ppm) 7.36 (s, 1H), 4.45 (t, *J* = 5.0 Hz, 2H), 3.71 (t, *J* = 5.0 Hz, 2H), 3.32 (s, 3H), 2.68 (t, *J* = 7.8 Hz, 2H), 1.63 (p, *J* = 7.8 Hz, 2H), 1.36 (h, *J* = 7.3 Hz, 2H), 0.90 (t, *J* = 7.4 Hz, 3H). ^{13}C NMR (101 MHz, $CDCl_3$): δ (ppm) 148.26, 121.61, 70.91, 58.93, 50.06, 31.52, 25.31, 22.29, 13.78. HRMS (ESI +) *m/z*: $[C_9H_{17}N_3O + H]^+$ calculated 184.14444, found 184.14445.

1.19 Synthesis of 4-propyl-1H-1,2,3-triazole-1-ethanol (N015)

Synthesised from General Procedure A; N015 (1.06 g, 6.3 mmol, 36%, pale yellow liquid) was obtained from sodium azide (26.2 mmol), 2-bromoethanol (17.5 mmol), $CuSO_4.5H_2O$ (1.0 mmol), sodium ascorbate (5.6 mmol) and 1-hexyne (25.5 mmol). The crude mixture was purified by silica chromatography (Pet. Ether/EtOAc, 2:3; R_f = 0.1). ^1H NMR (400 MHz, $CDCl_3$): δ (ppm) 7.41 (s, 1H), 4.70 (s, 1H), 4.37 (t, *J* = 5.2 Hz, 2H), 3.95 (t, *J* = 5.1 Hz, 2H), 2.62 – 2.53 (m, 2H), 1.54 (p, *J* = 7.4 Hz, 2H), 1.29 (h, *J* = 7.3 Hz, 2H), 0.85 (t, *J* = 7.4 Hz, 3H). ^{13}C NMR (101 MHz, $CDCl_3$): δ (ppm) 147.87, 121.99, 60.71, 52.62, 31.36, 25.11, 22.20, 13.73. HRMS (ESI +) *m/z*: $[C_8H_{15}N_3O + H]^+$ calculated 170.12879, found 170.12887.

1.20. Synthesis of 1,4-dipropyl-1H-1,2,3-triazole (N016)

Synthesised from General Procedure A, N016 (0.68 g, 4.36 mmol, 26%, colourless oil) was obtained from sodium azide (20.0 mmol), 1-bromopropane (17.0 mmol), $CuSO_4.5H_2O$ (1.0 mmol), sodium ascorbate (5.1 mmol) and 1-pentyne (20.1 mmol). The crude mixture was purified by silica chromatography (Pet. Ether/EtOAc, 2:1; R_f = 0.23). ^1H NMR (400 MHz, $CDCl_3$): δ (ppm) 7.27 (s, 1H), 4.28 (t, *J* = 7.1 Hz, 2H), 2.70 (t, *J* = 7.6 Hz, 2H), 1.92 (h, *J* = 7.3 Hz, 2H), 1.70 (h, *J* = 7.4 Hz, 2H), 1.01 – 0.90 (m, 6H). ^{13}C NMR (101 MHz, $CDCl_3$): δ (ppm) 148.20, 120.64, 51.91, 27.76, 23.87, 22.84, 13.89, 11.19. HRMS (ESI +) *m/z*: $[C_8H_{15}N_3 +H]^+$ calculated 154.13387, found 154.13385.

1.21. Synthesis of 1-(3-butyn-1-yl)-4-propyl-1H-1,2,3-triazole (N017)

Synthesised from a modified General Procedure A and a reported procedure (Di Francesco, M. E., *et al*. 2016. Gls1 Inhibitors for Treating Disease. Board of Regents, The University of Texas System. US Patent US20160002248A1). N017 (0.30g, 1.8 mmol, 18%, pale yellow oil) was obtained from sodium azide (15.0 mmol) and 1-bromobutyne (10.0 mmol), which were heated at 60°C in DMF (15 mL) for 3 hours. The reaction was cooled to room temperature and diluted with H_2O (15 mL), followed by addition of $CuSO_4.5H_2O$ (1.2 mmol), sodium ascorbate (2.0 mmol) and 1-pentyne (17.9 mmol). After stirring at room temperature overnight and workup, the crude mixture was purified by silica chromatography (Pet. Ether/Diethyl Ether, 2:1; R_f = 0.17). ^1H NMR (400 MHz, $CDCl_3$): δ (ppm) 7.41 (s, 1H), 4.47 (t, *J* = 6.7 Hz, 2H), 2.77 (td, *J* = 6.7, 2.6 Hz, 2H), 2.70 (t, *J* = 7.6 Hz, 2H), 2.07 (t, *J* = 2.7 Hz, 1H), 1.70 (h, *J* = 7.4 Hz, 2H), 0.96 (t, *J* = 7.4 Hz, 3H) . ^{13}C NMR (101 MHz, $CDCl_3$): δ (ppm) 148.04, 121.20, 79.63, 71.37, 48.64, 27.53, 22.64, 20.64, 13.70. HRMS (ESI +) *m/z*: $[C_9H_{13}N_3 +H]^+$ calculated 164.11822, found 164.11824.

2. Spectra of Inhibitors N001 – N017

2.1 ^{1}H NMR and ^{13}C NMR Spectra of N001

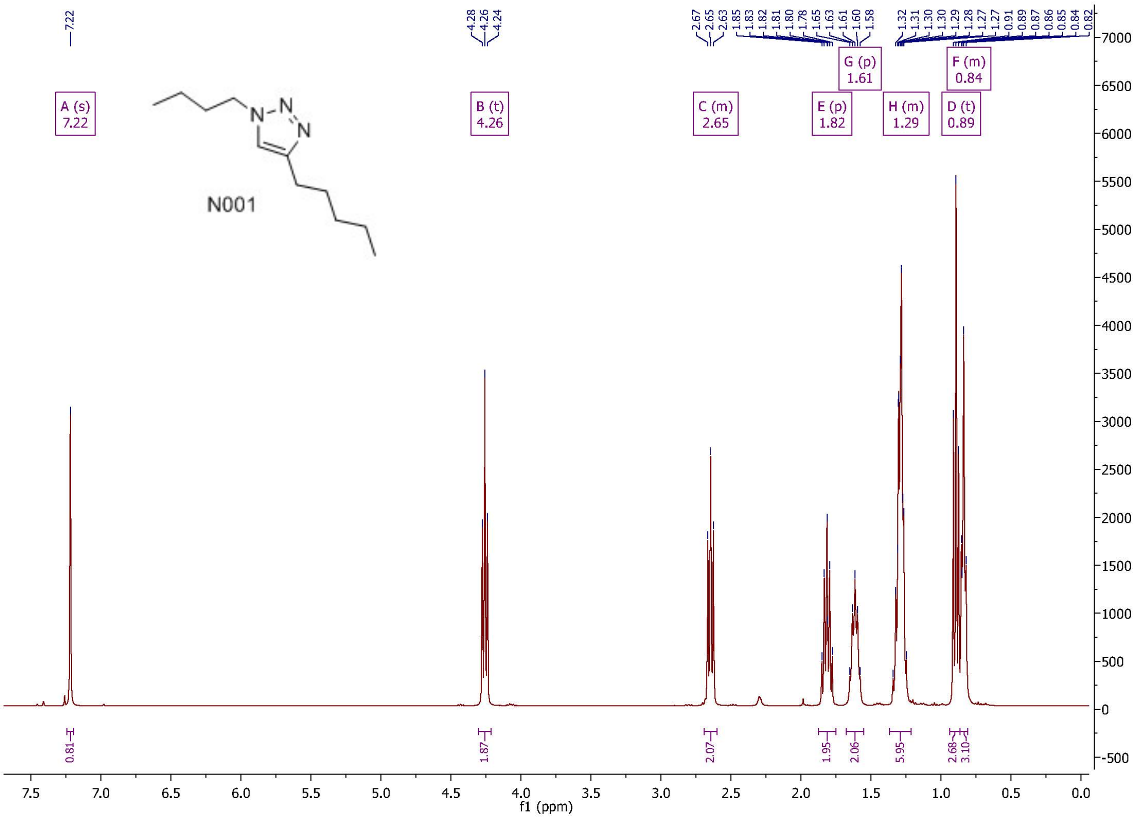

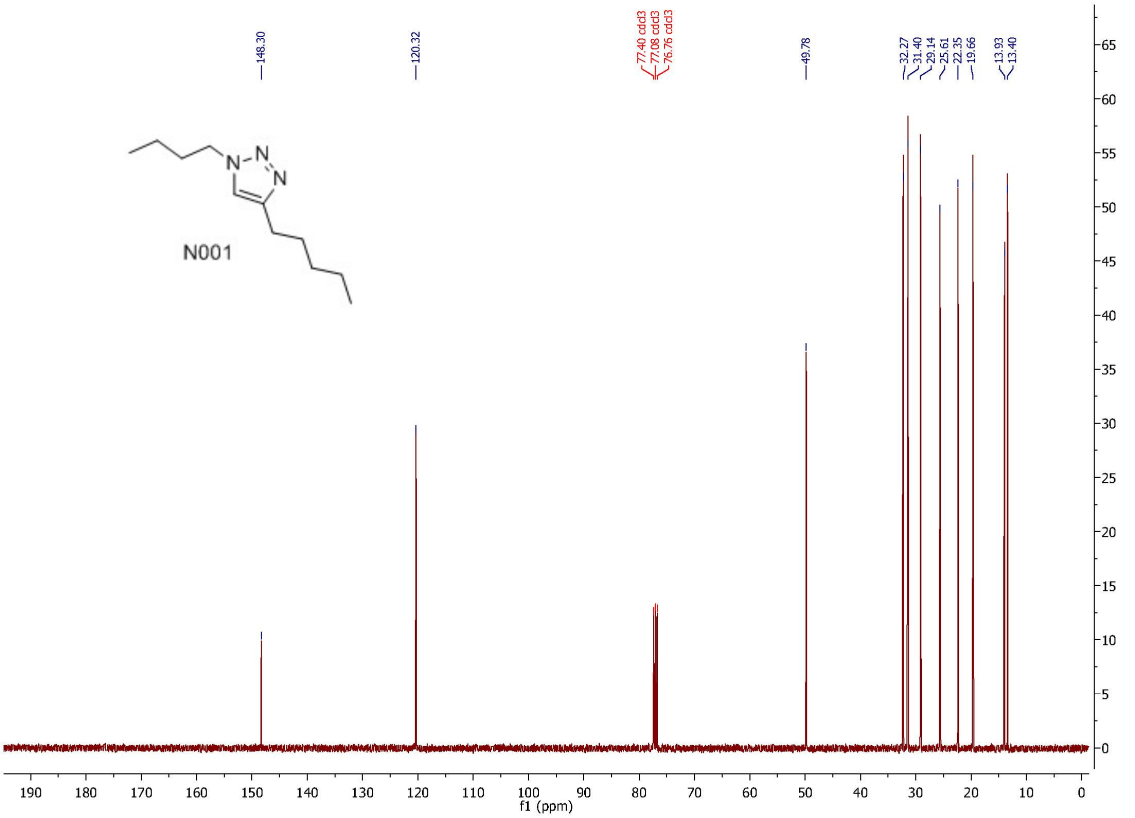

2.2 ^{1}H NMR and ^{13}C NMR Spectra of N002

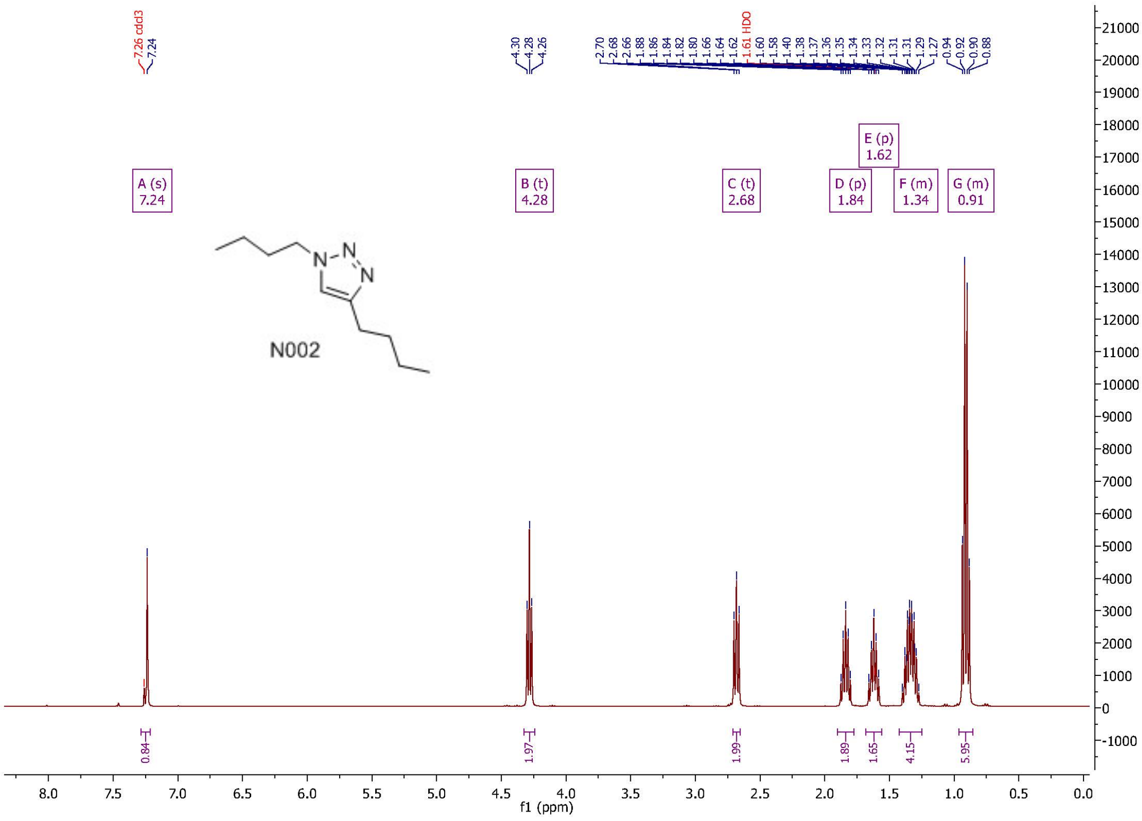

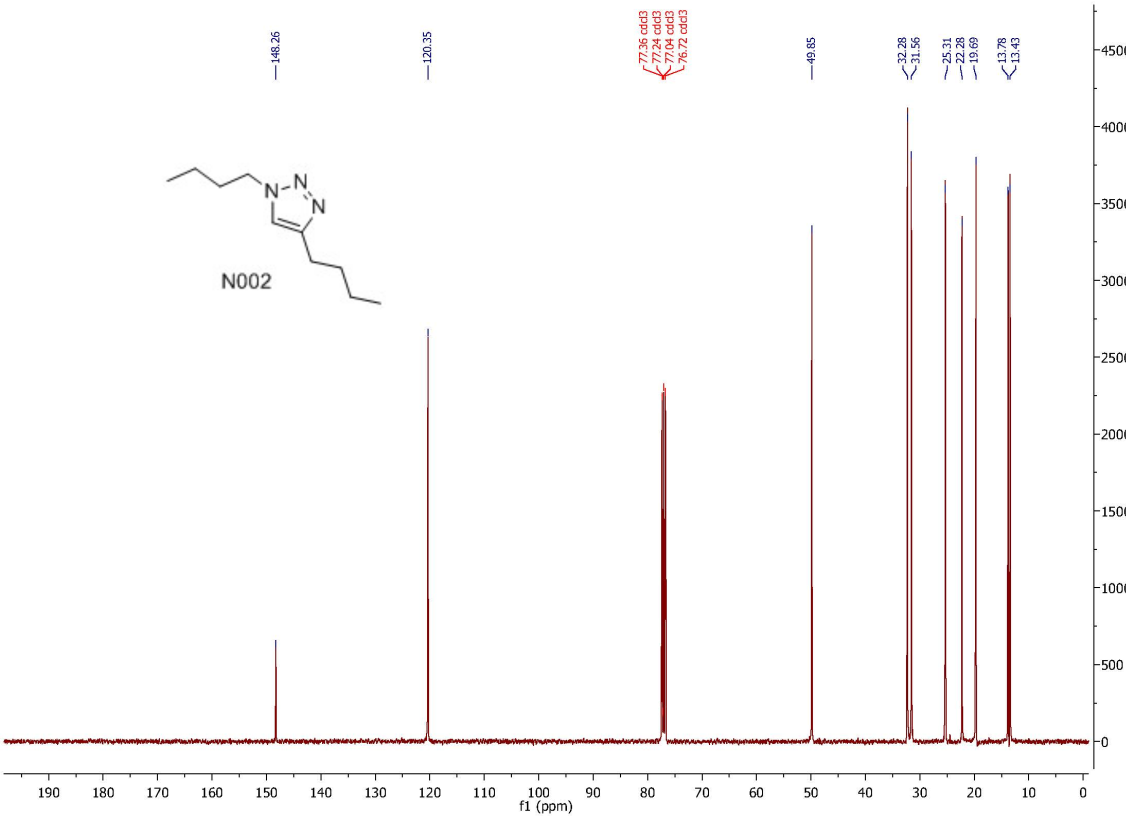

2.3 ^{1}H NMR and ^{13}C NMR Spectra of N003

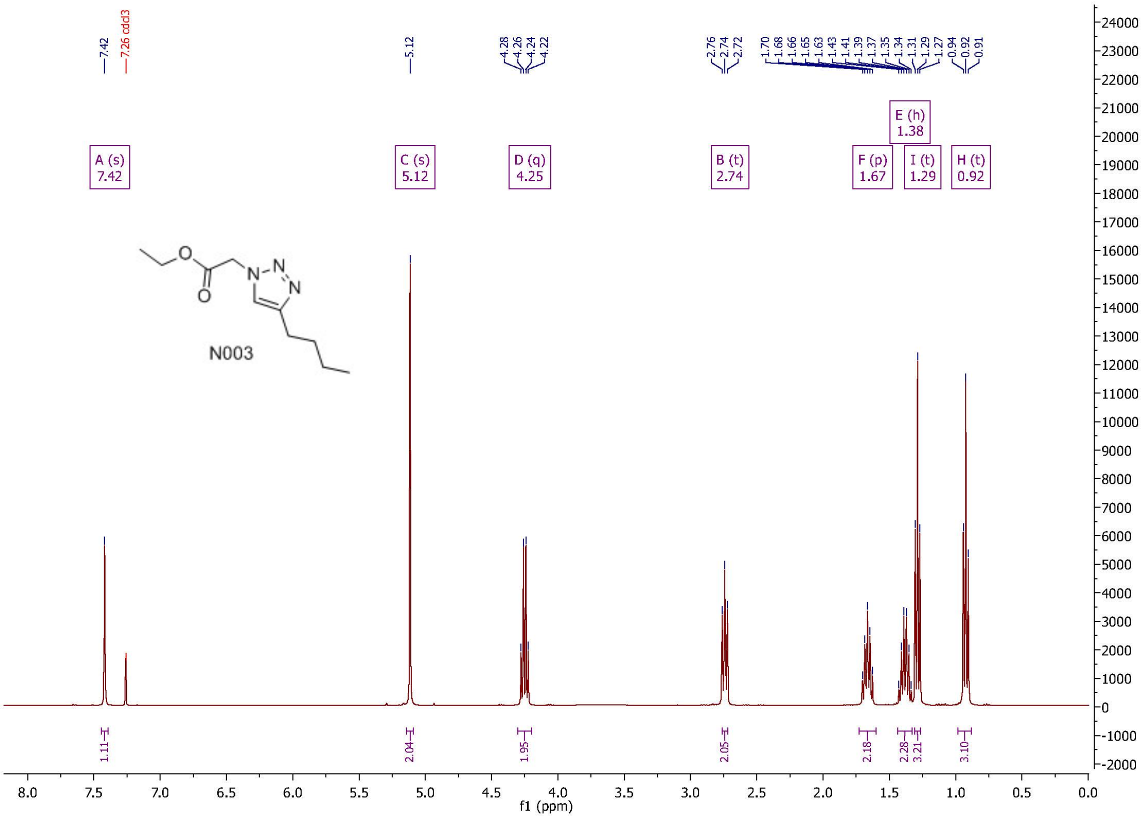

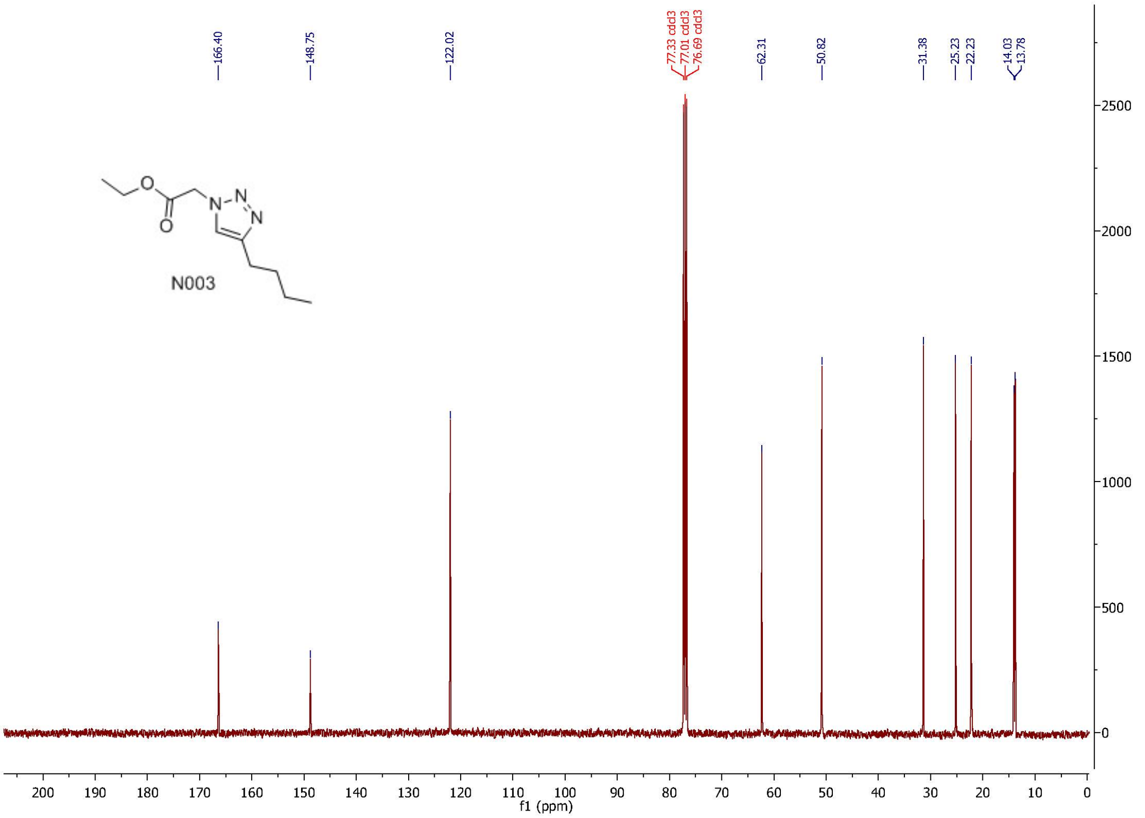

2.4 ^1H NMR and ^{13}C NMR Spectra of N004

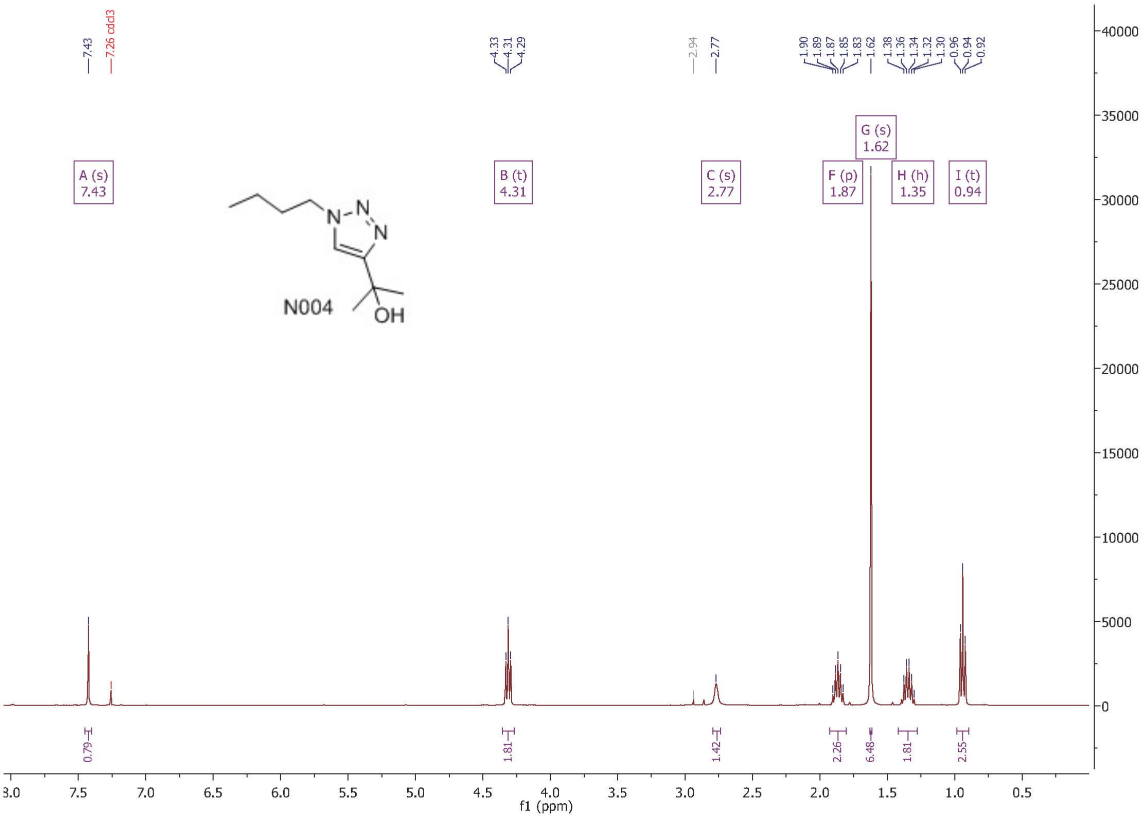

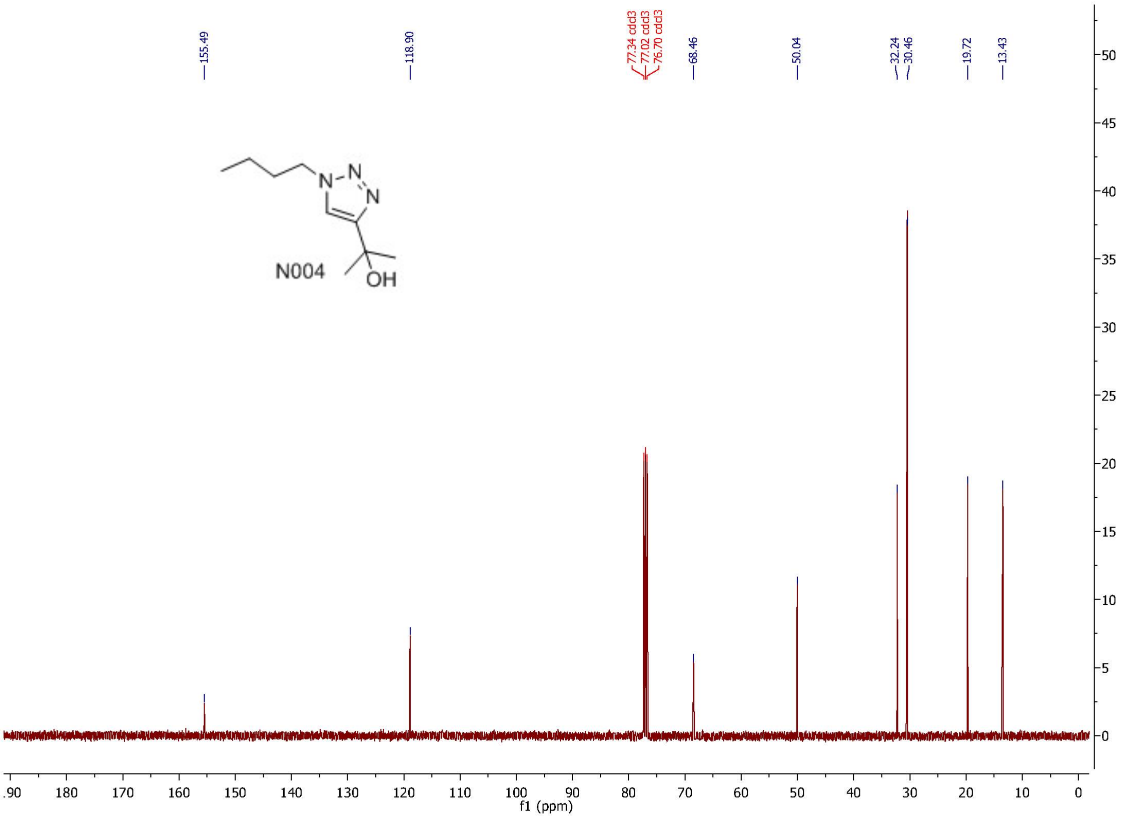

2.5 ^{1}H NMR and ^{13}C NMR Spectra of N005

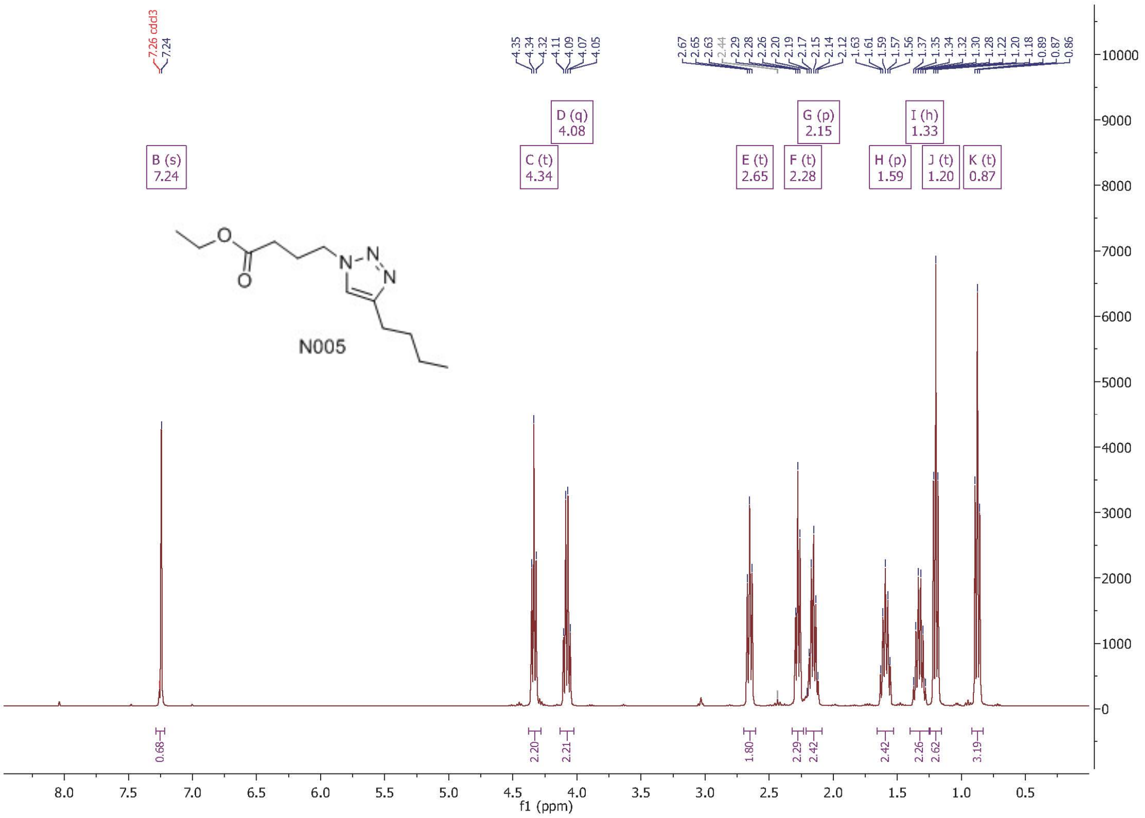

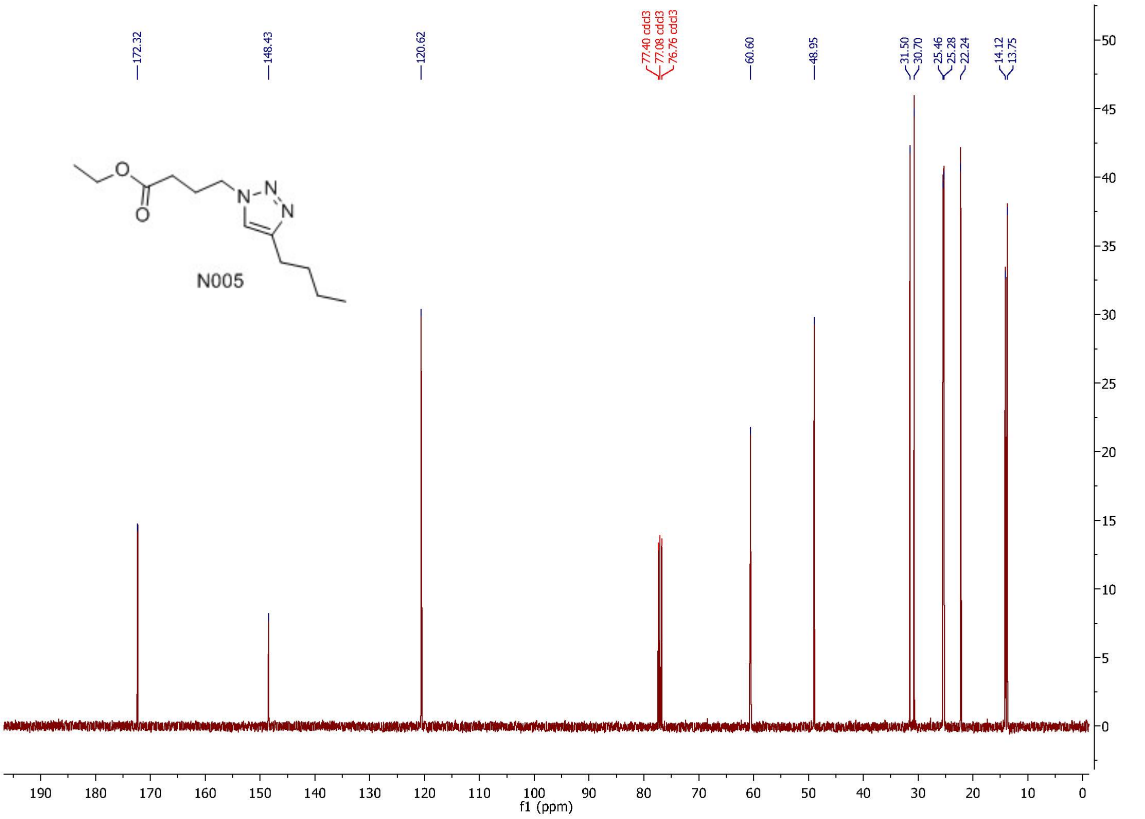

2.6 ^{1}H NMR and ^{13}C NMR Spectra of N006

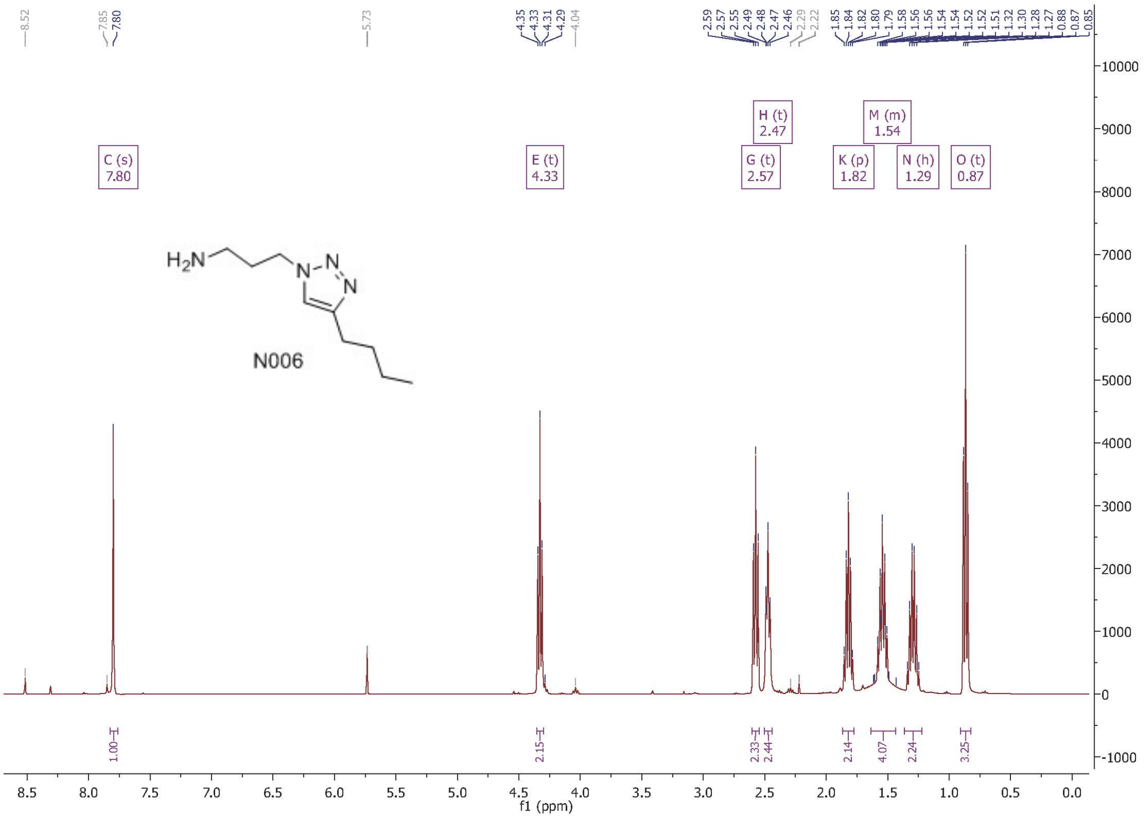

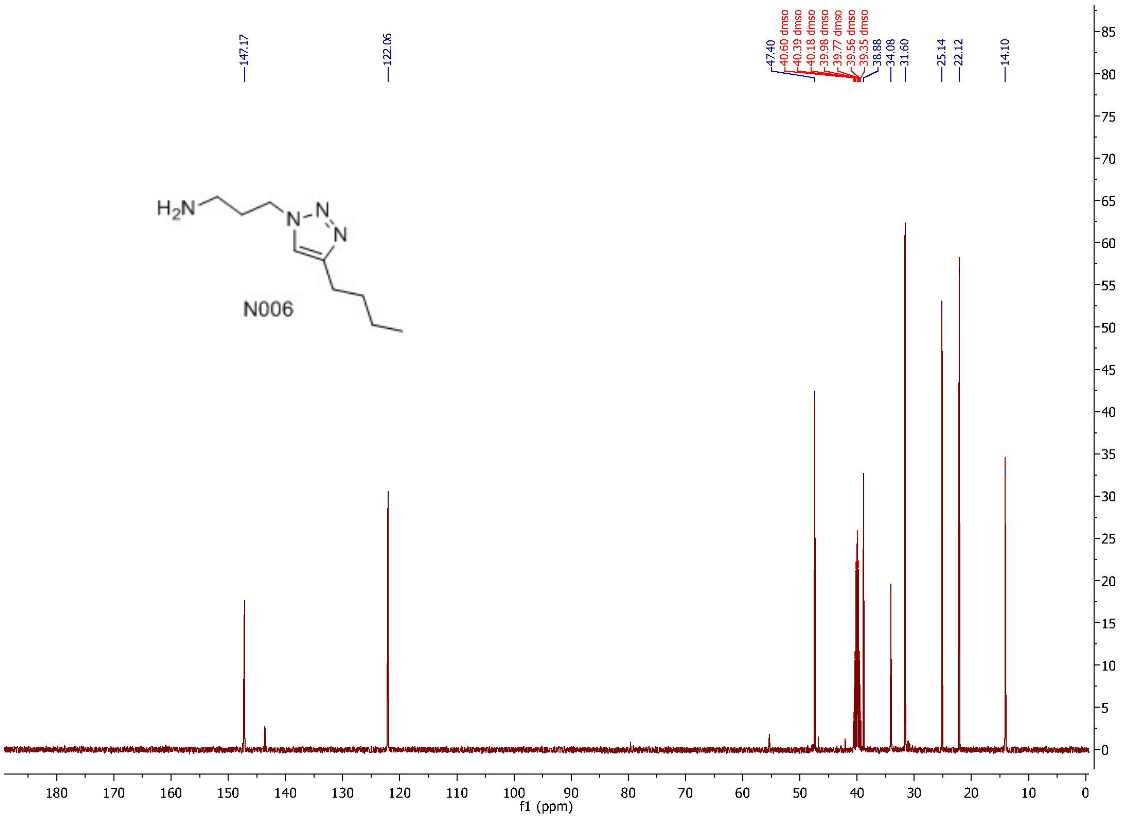

2.7 ^{1}H NMR and ^{13}C NMR Spectra of N007

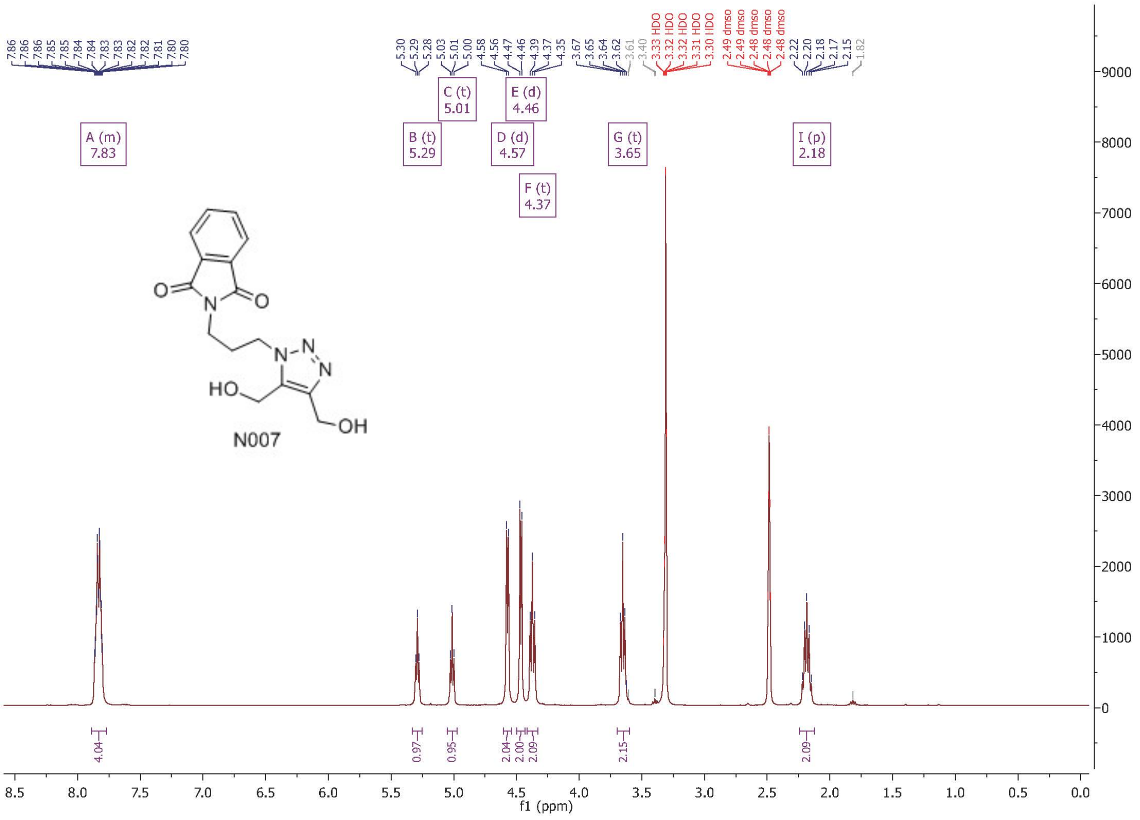

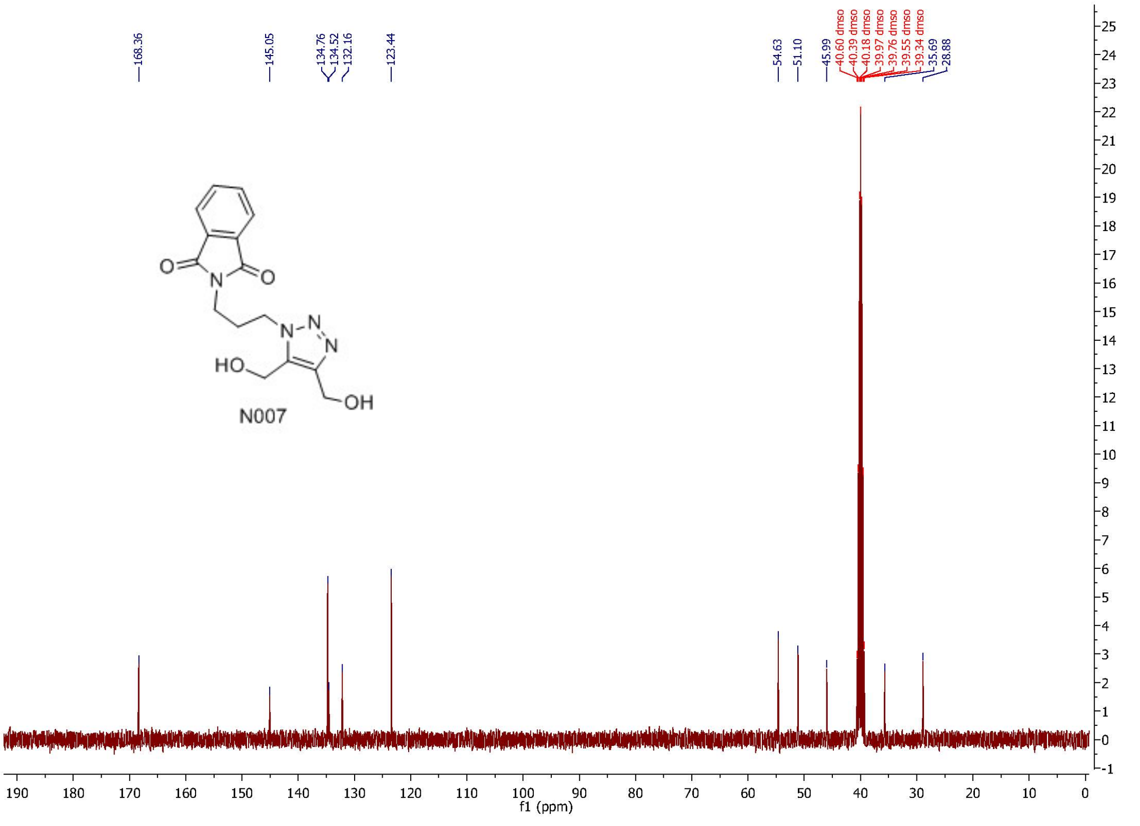

2.8 ^{1}H NMR and ^{13}C NMR Spectra of N008

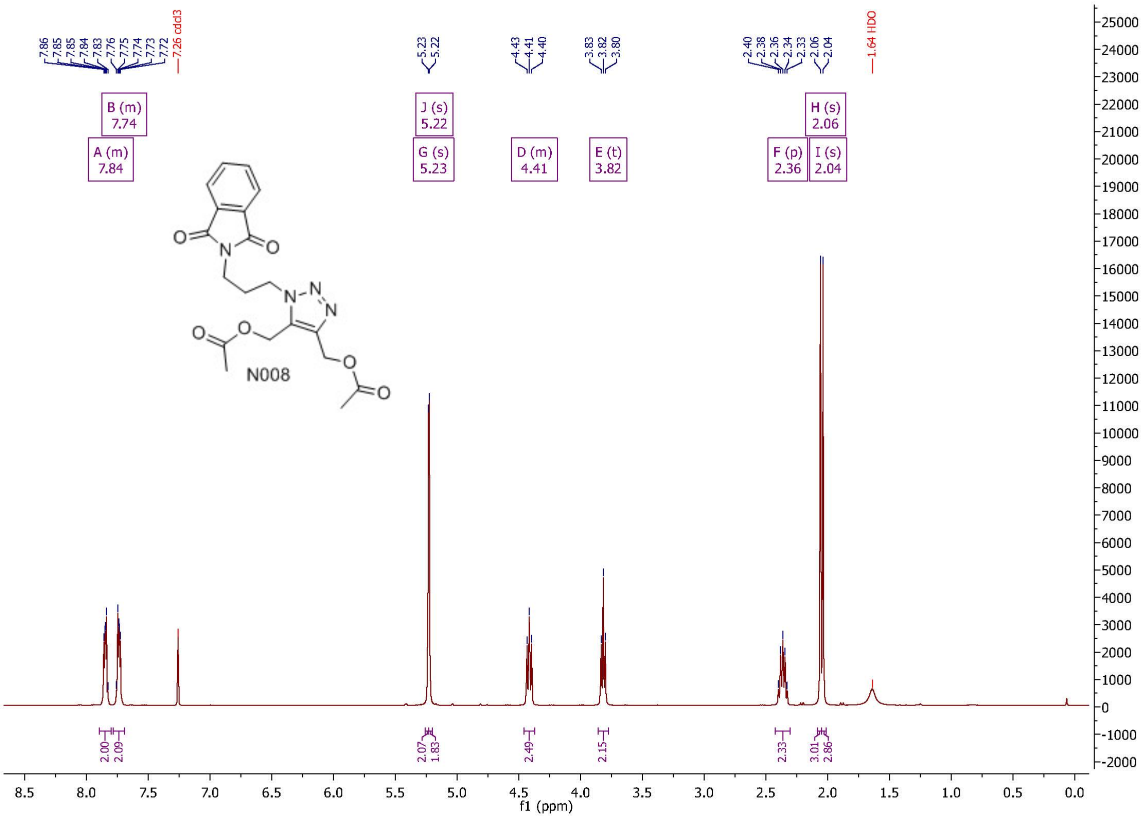

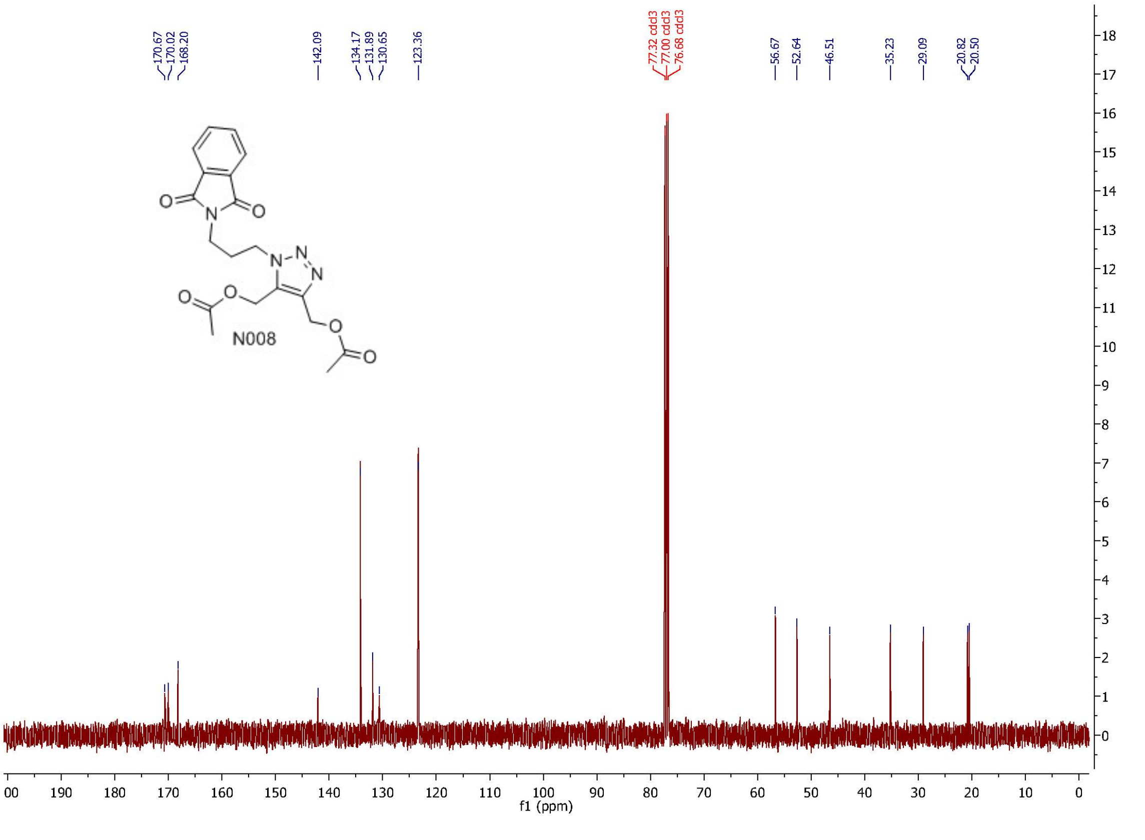

2.9 ^{1}H NMR and ^{13}C NMR Spectra of N009

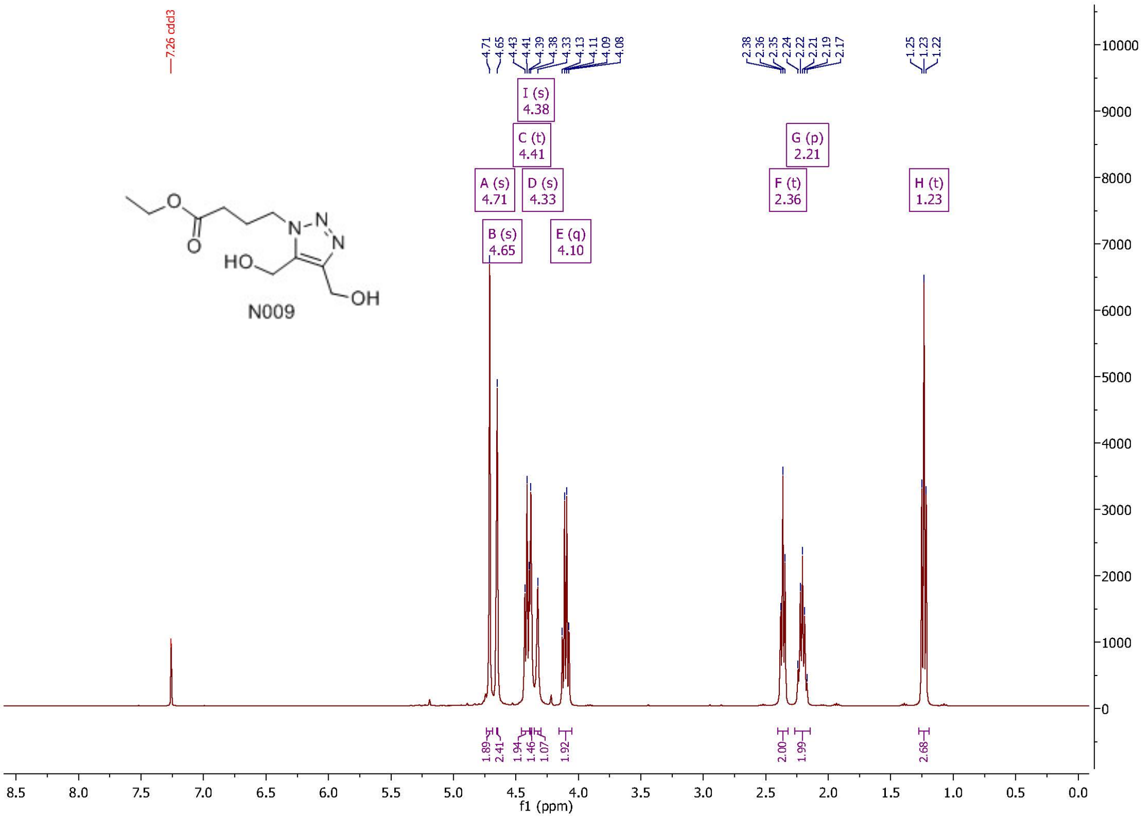

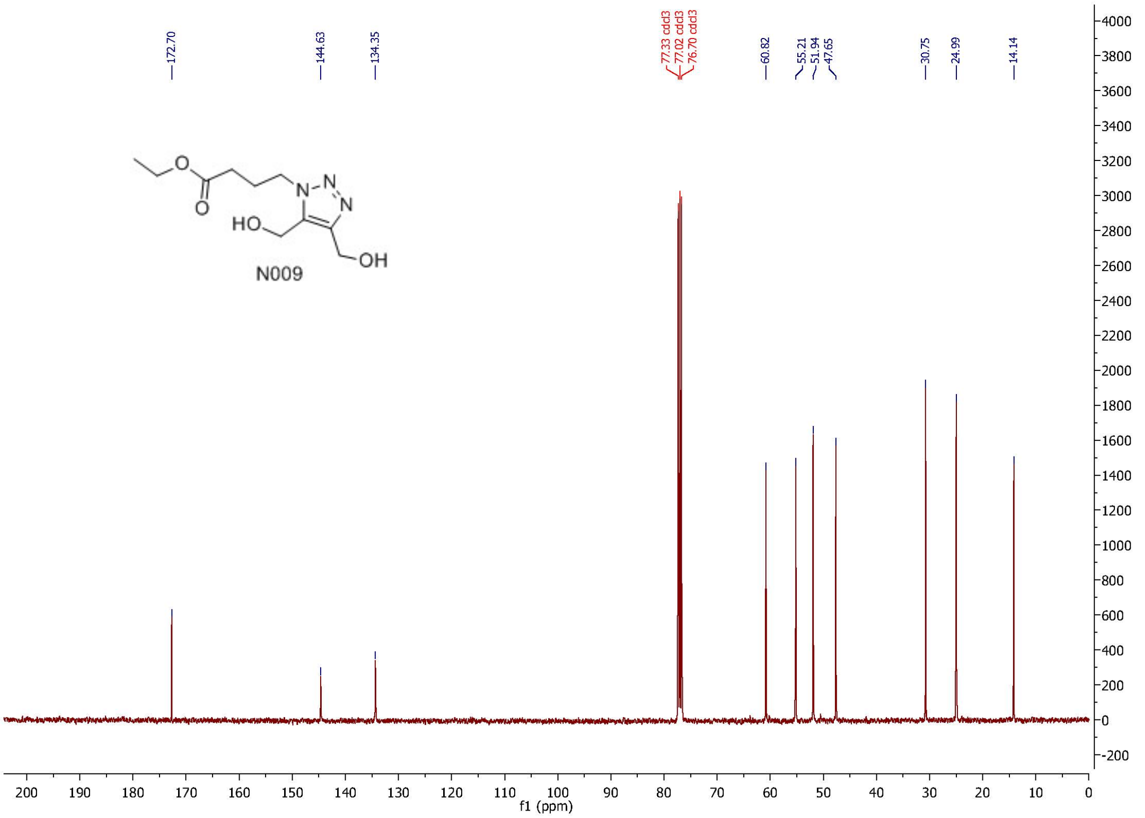

2.10 ^{1}H NMR and ^{13}C NMR Spectra of N010

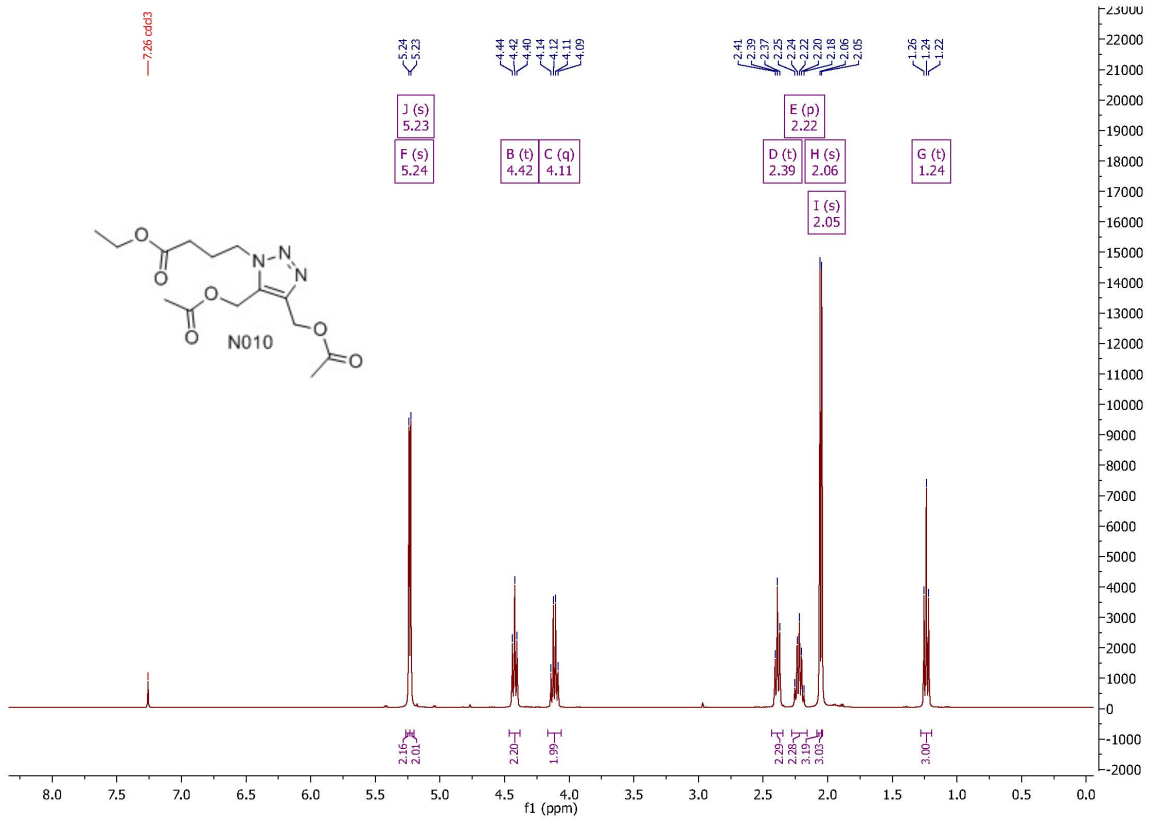

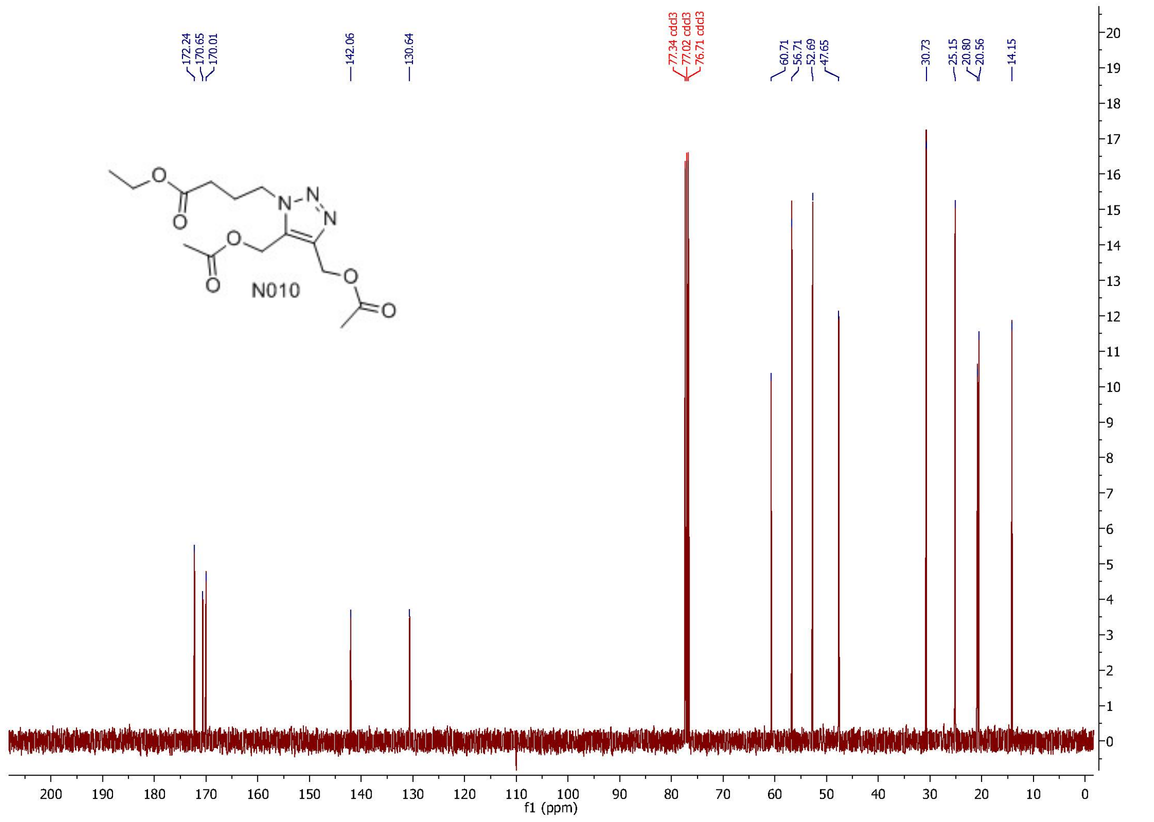

2.11 ^{1}H NMR and ^{13}C NMR Spectra of N011

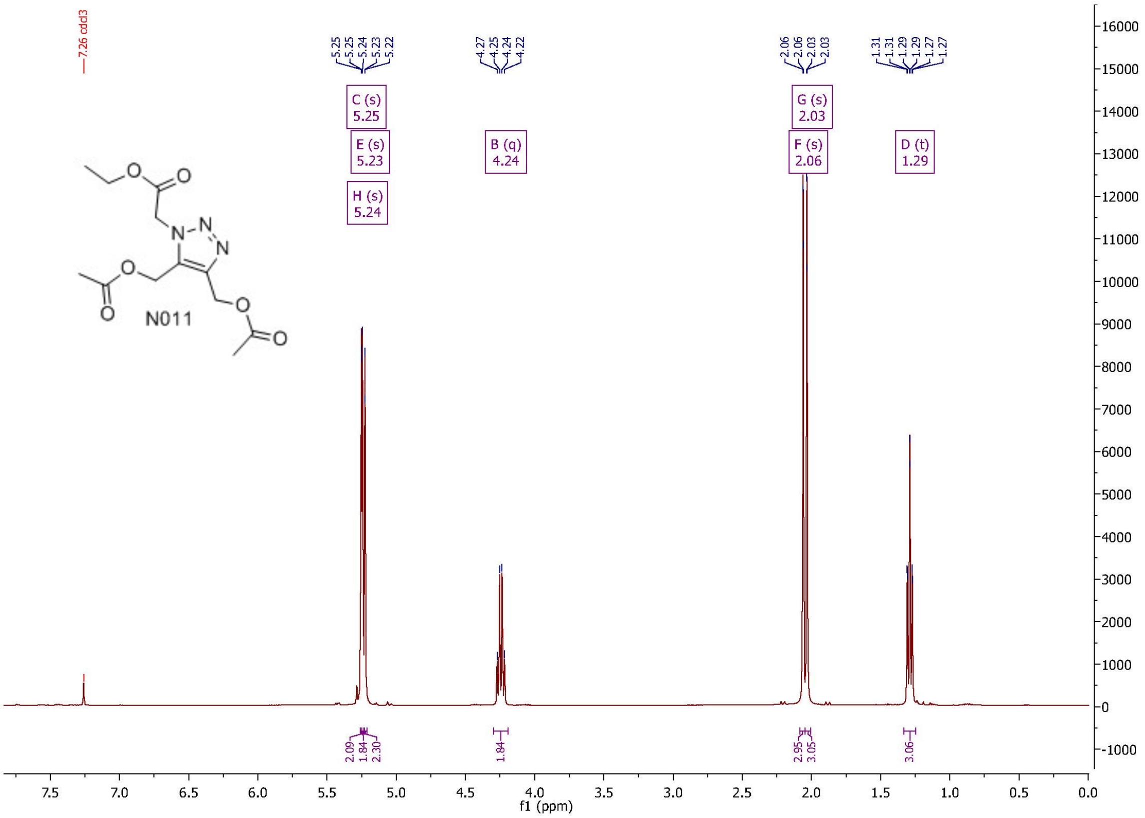

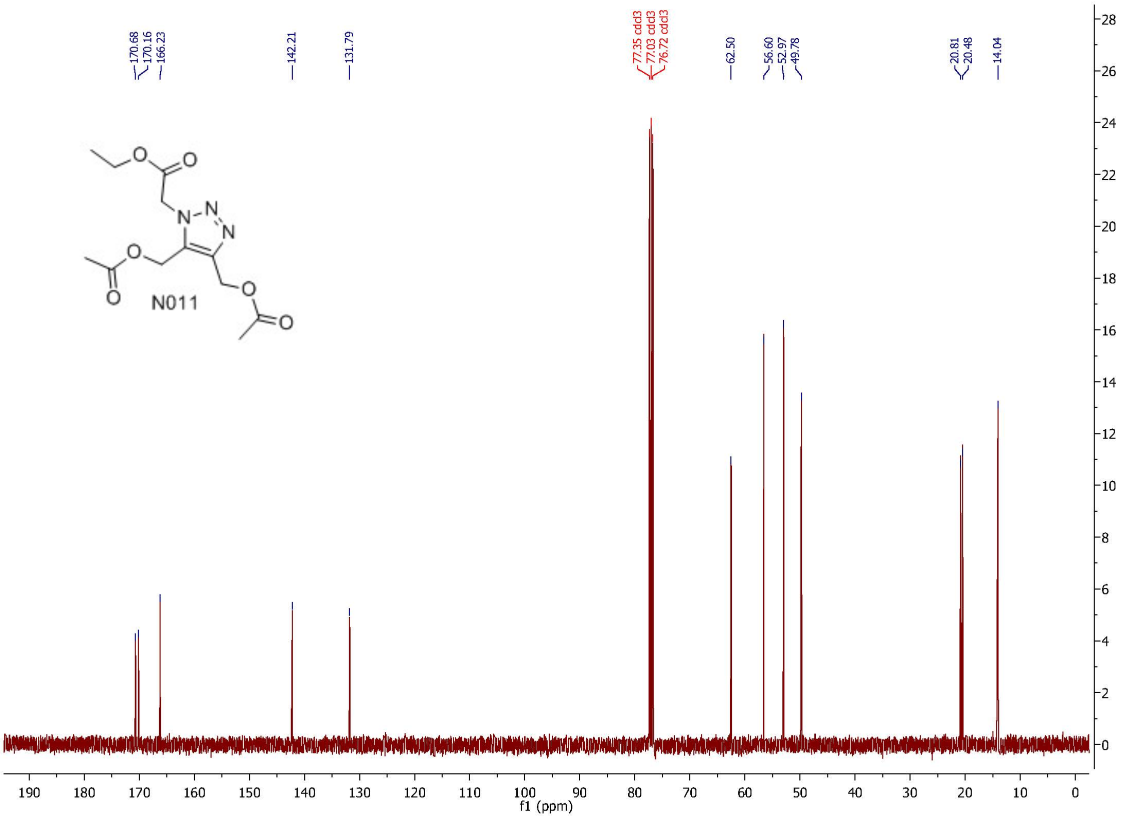

2.12 ^{1}H NMR and ^{13}C NMR Spectra of N012

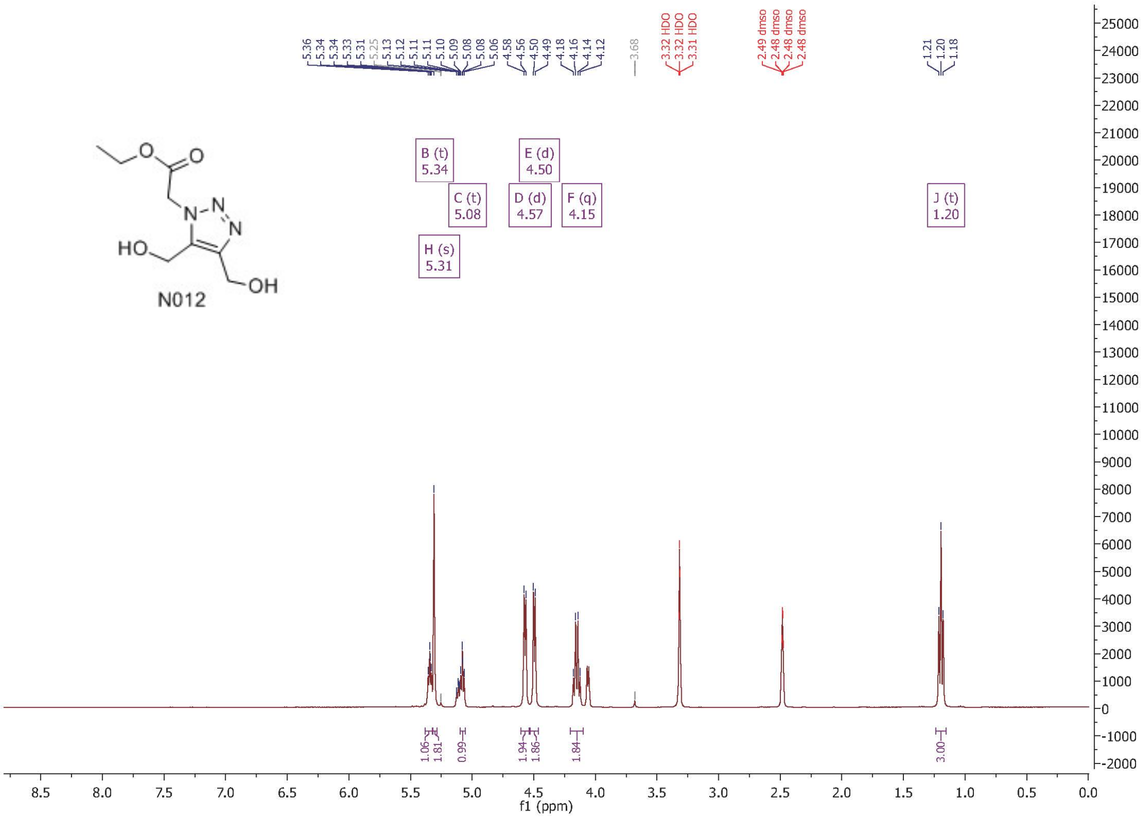

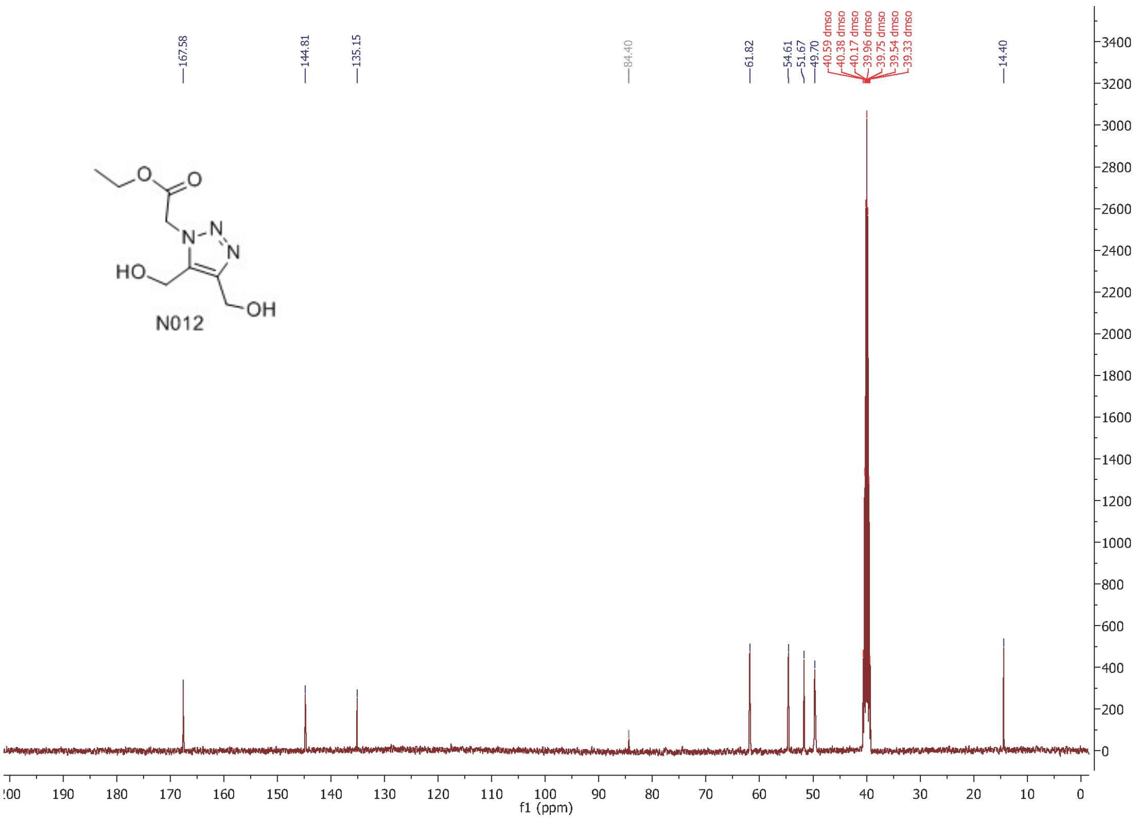

2.13 ^{1}H NMR and ^{13}C NMR Spectra of N013

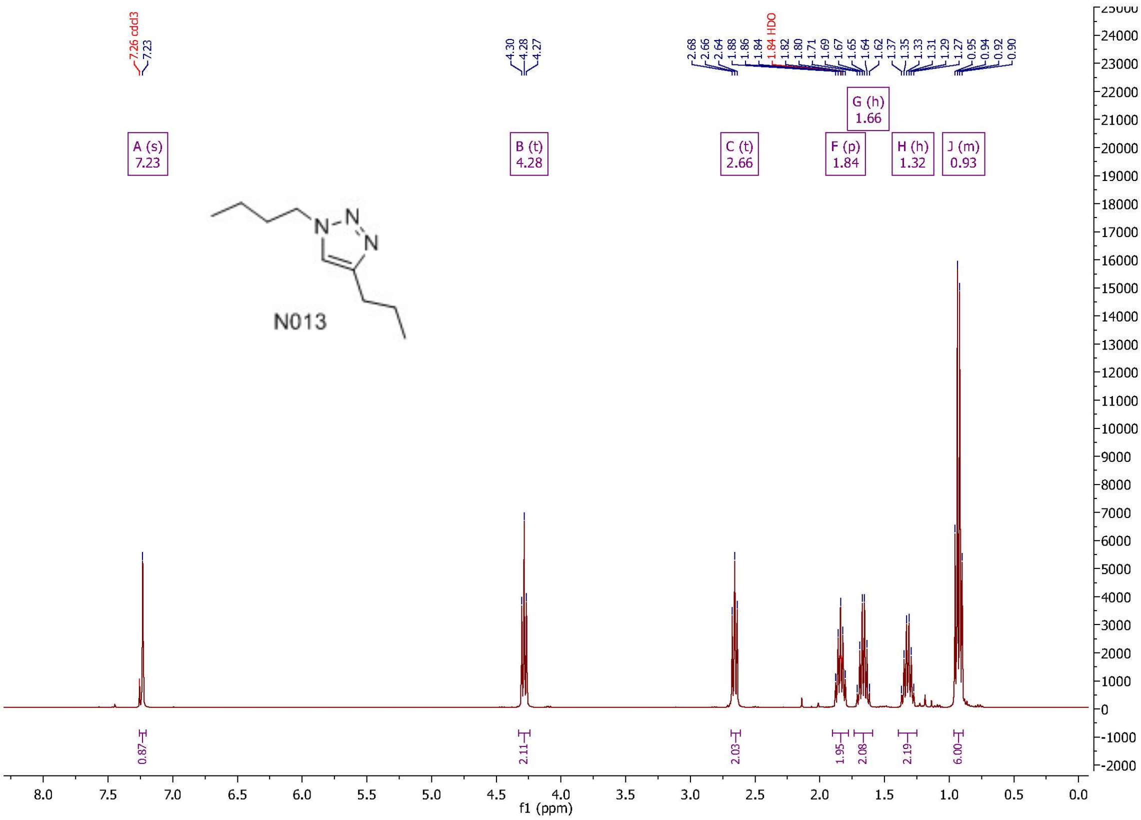

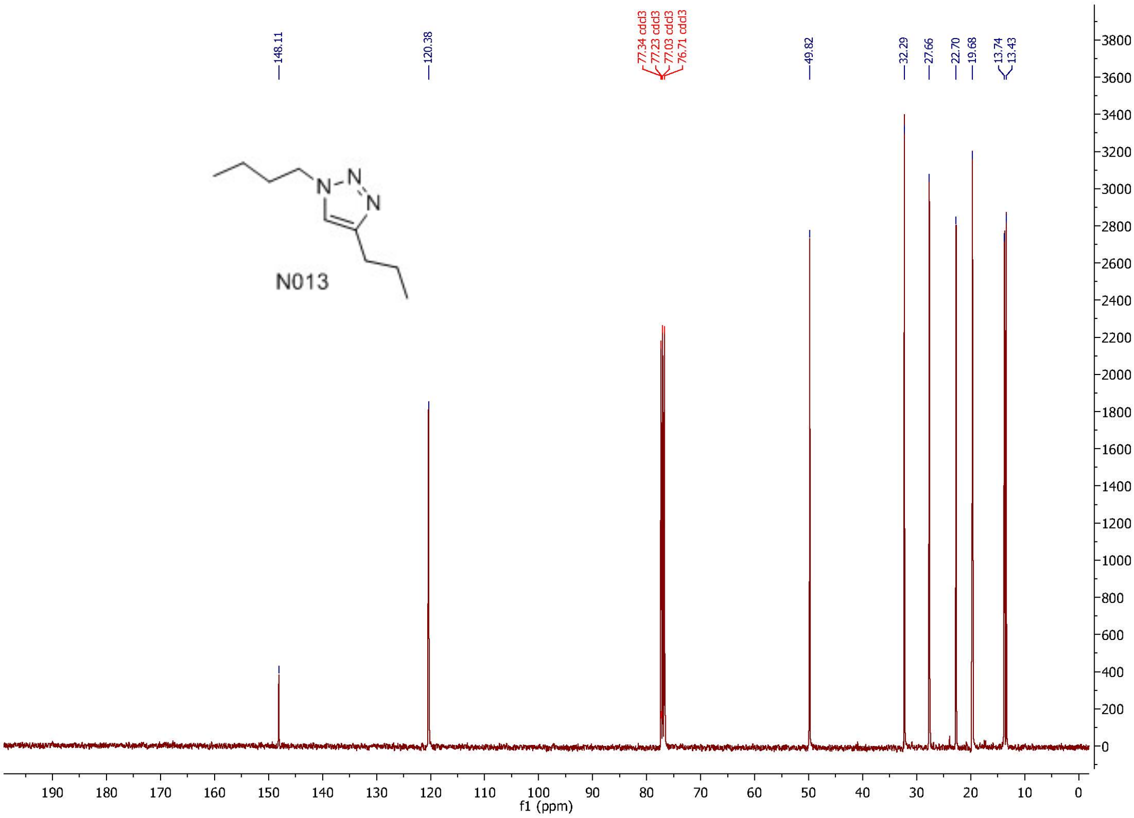

2.14 ^{1}H NMR and ^{13}C NMR Spectra of N014

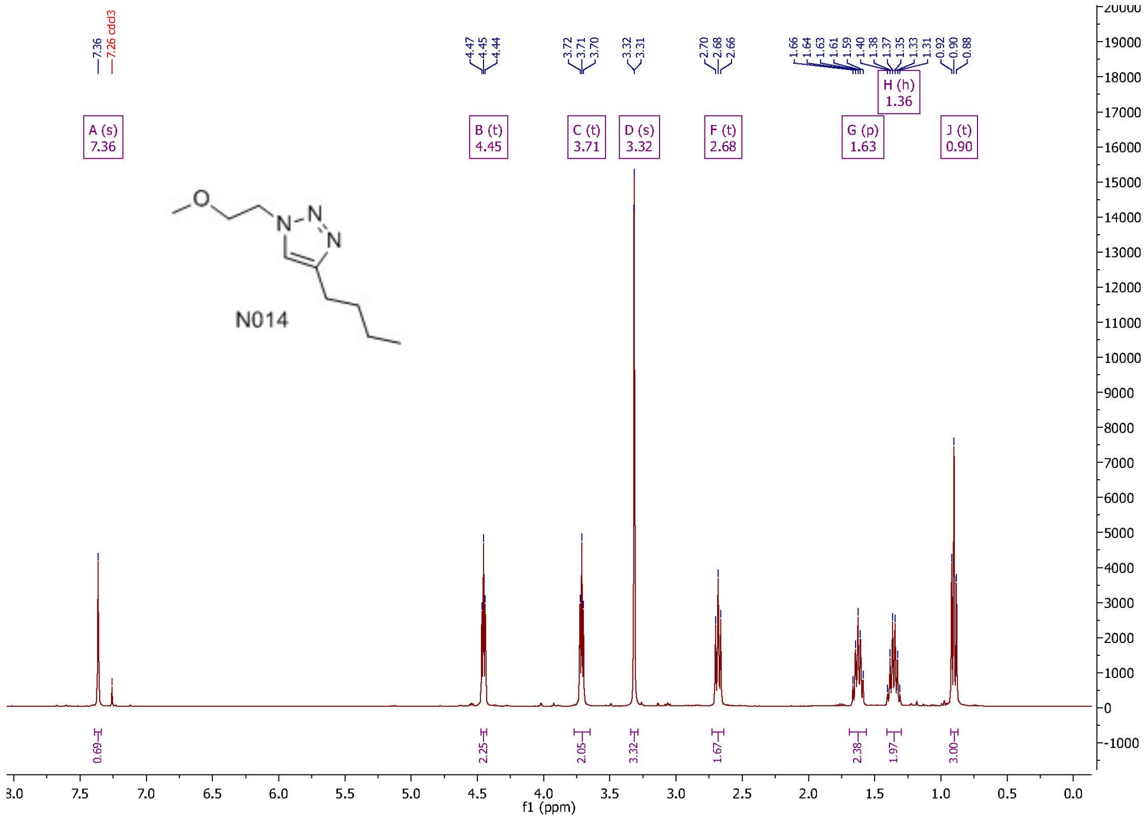

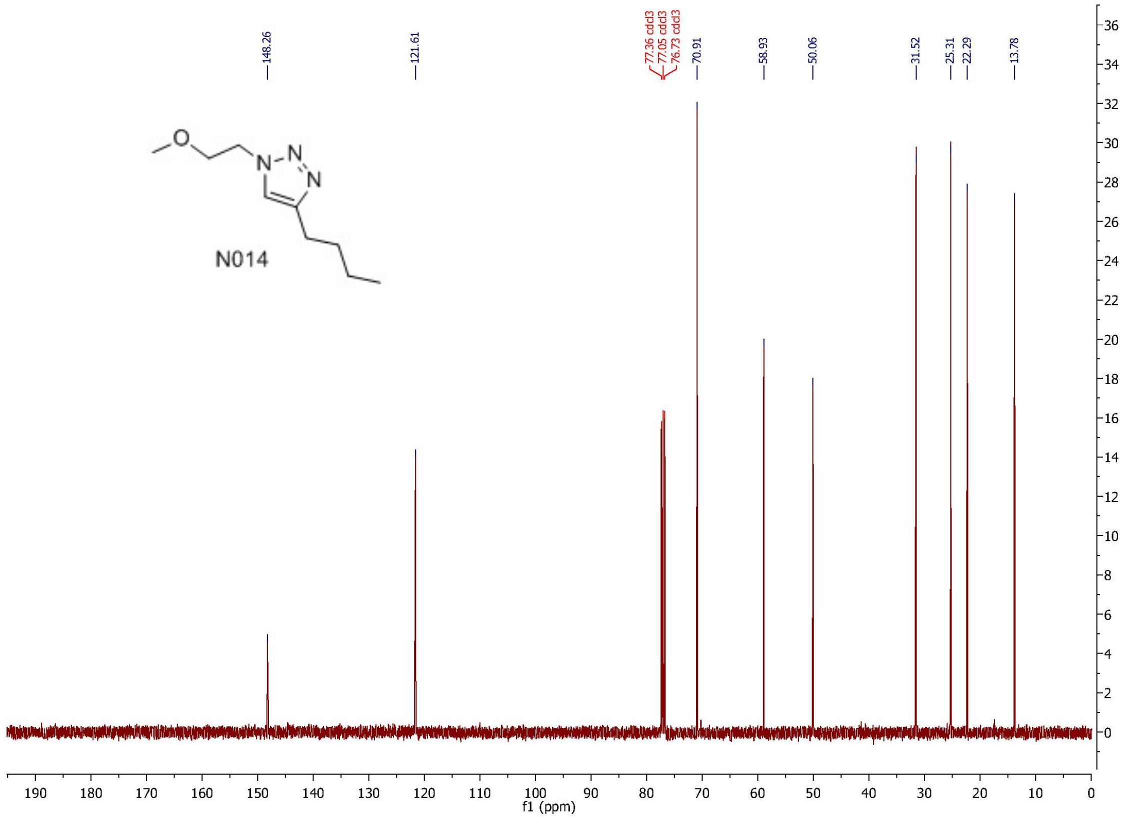

2.15 ^{1}H NMR and ^{13}C NMR Spectra of N015

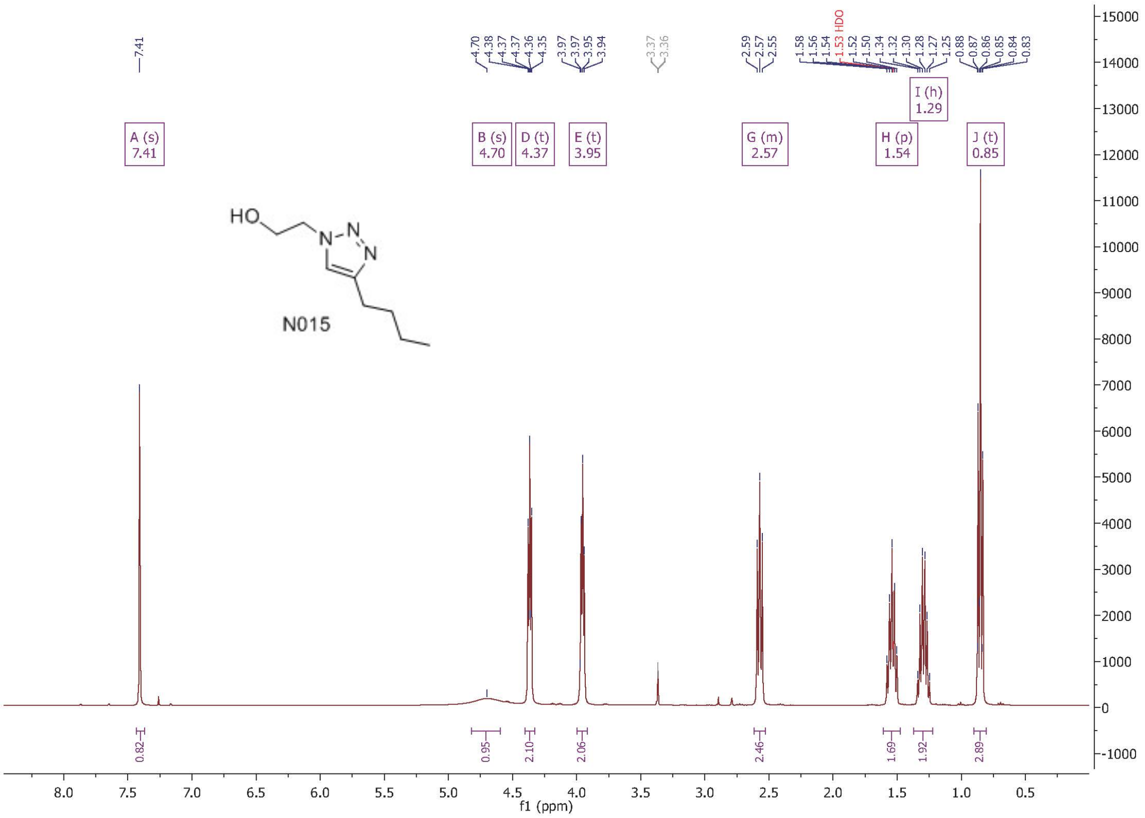

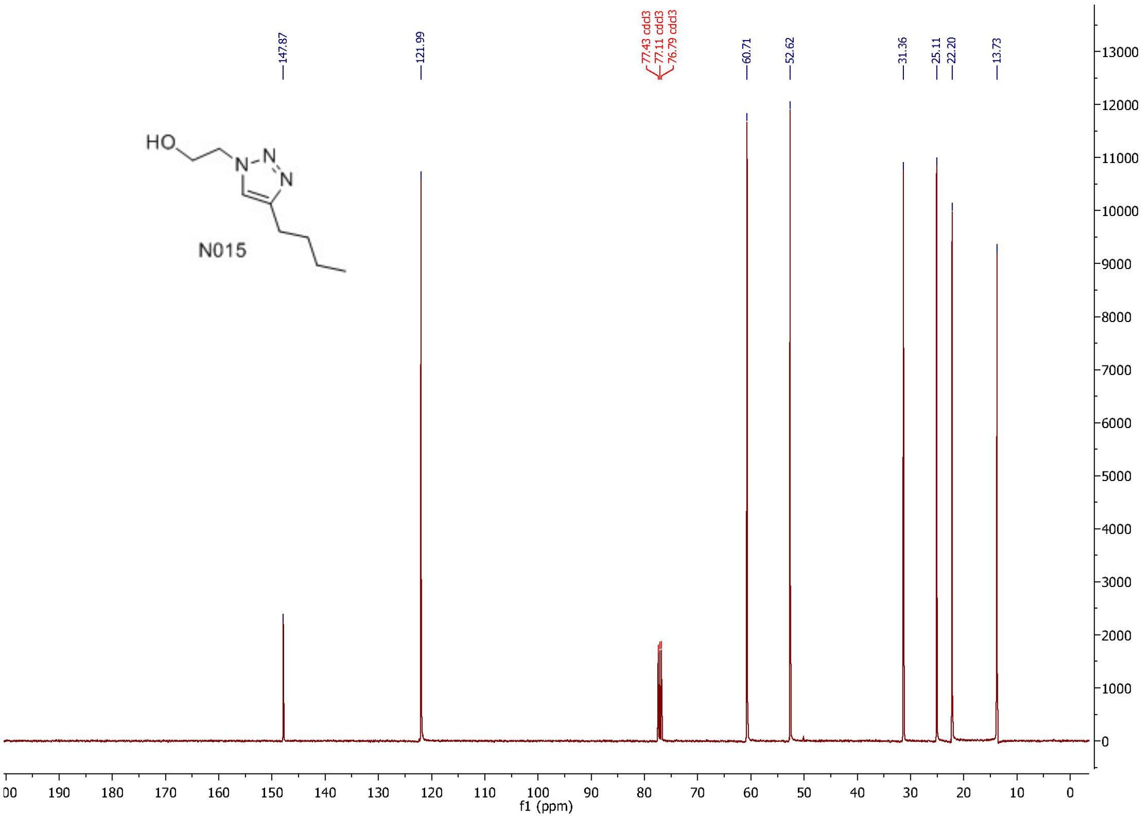

2.16 ^{1}H NMR and ^{13}C NMR Spectra of N016

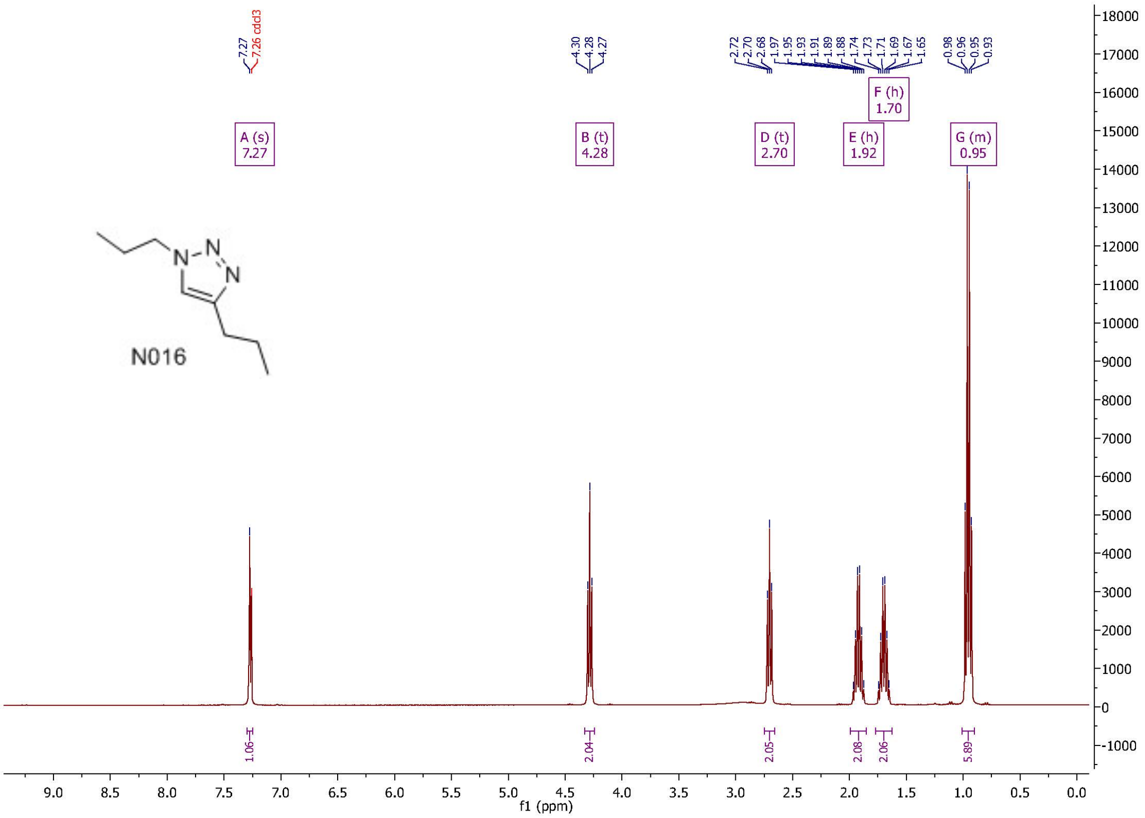

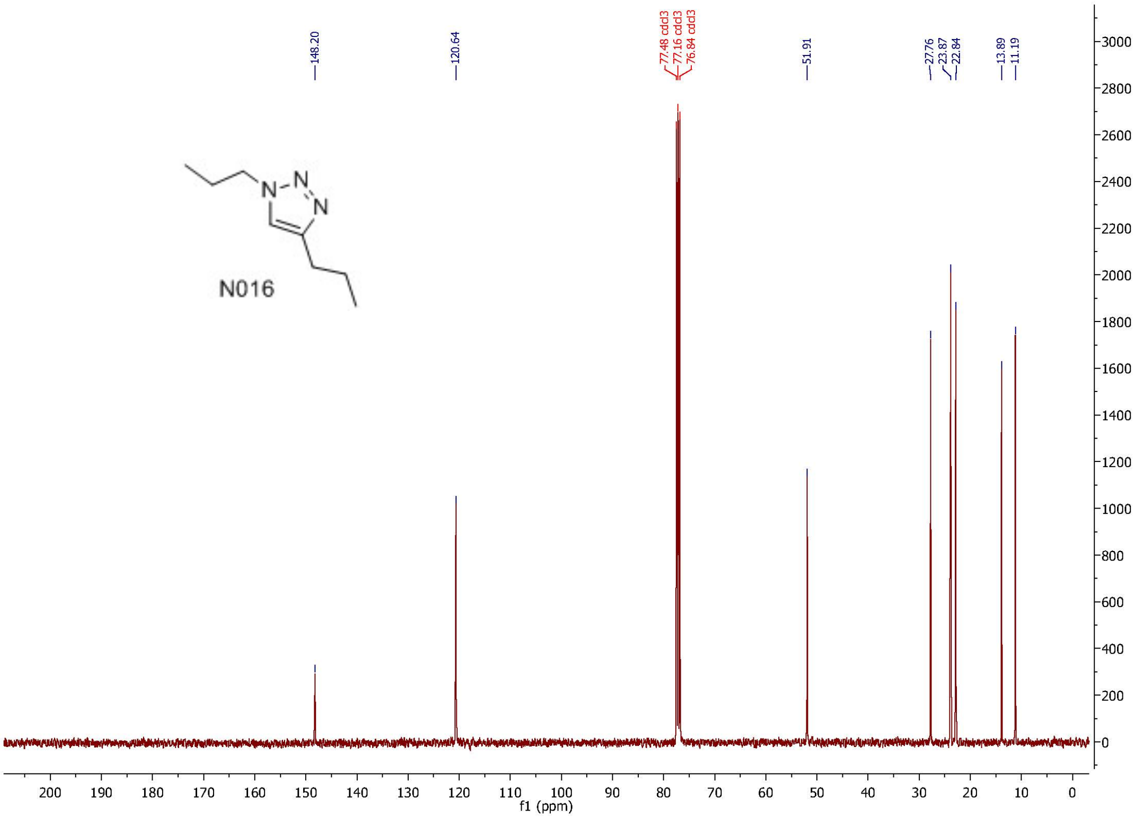

2.17 ^{1}H NMR and ^{13}C NMR Spectra of N017

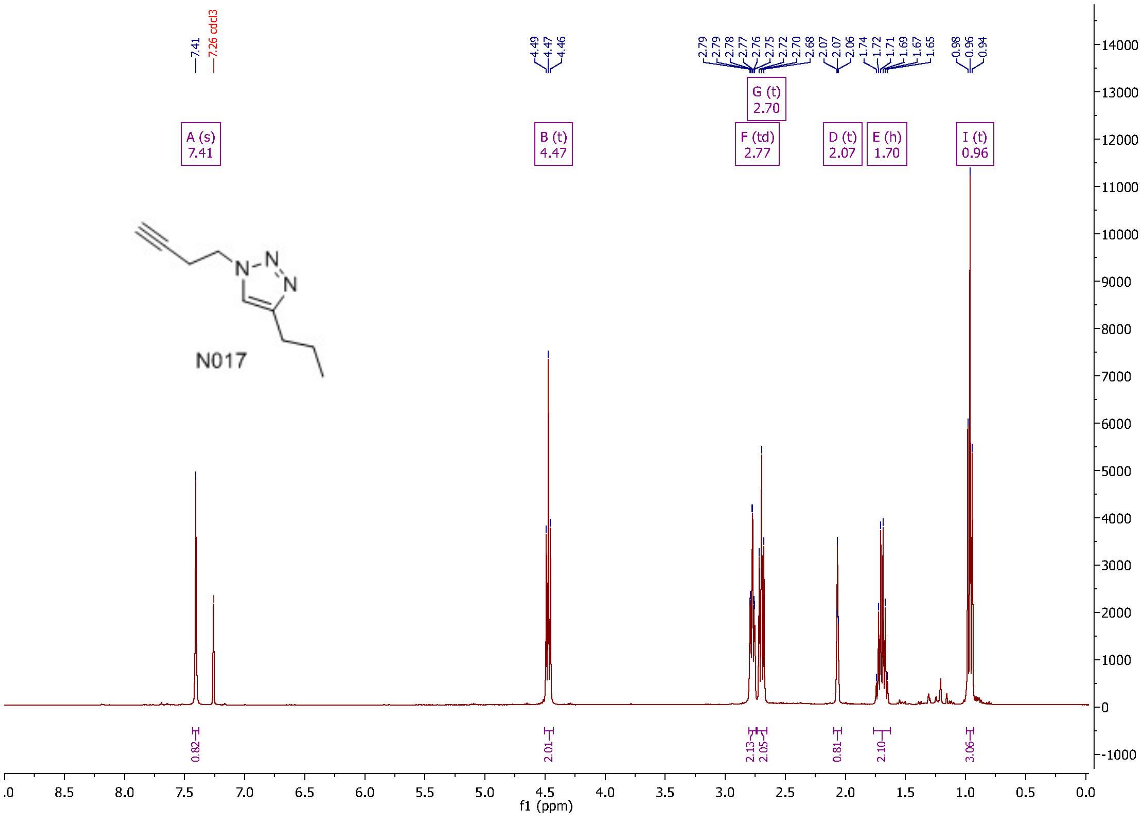

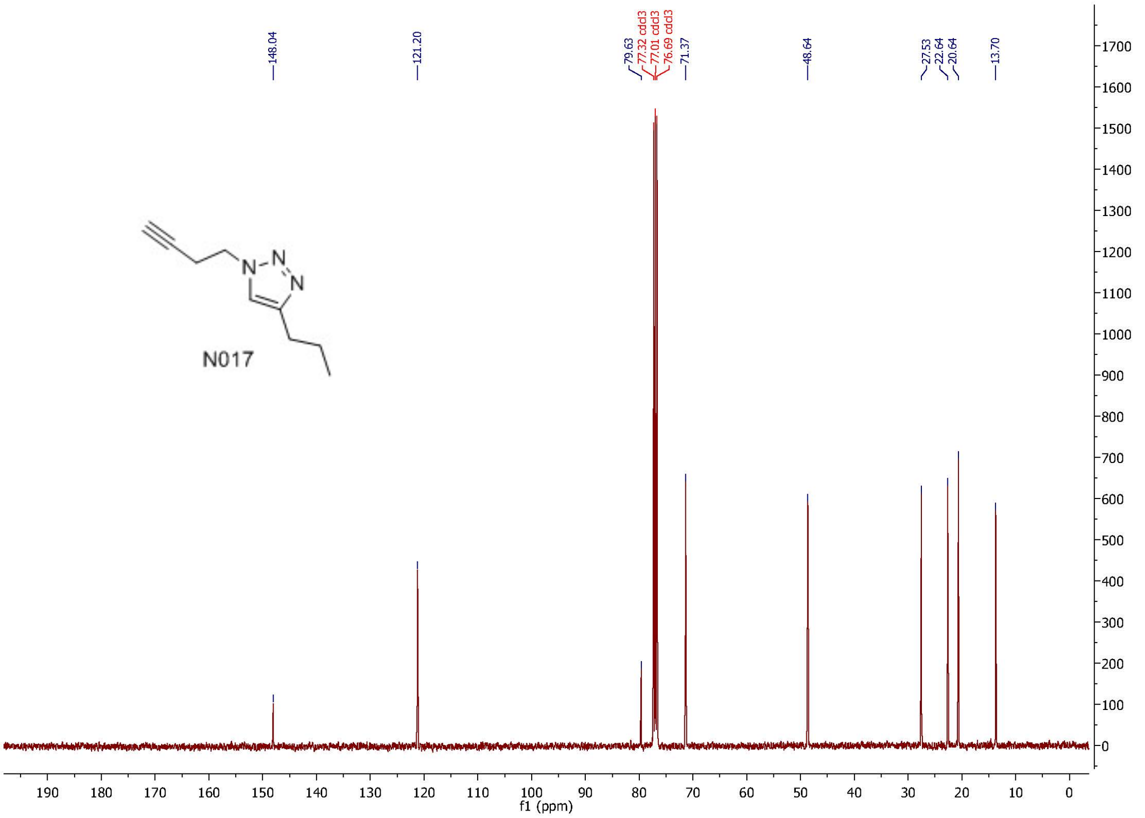

3. Soil Incubation Studies

Table S2. Properties of the soils used in this study.

Soil Property	Horsham	Dahlen
Organic C (%)	0.73	1.02
pH (1:5 water)	8.8	7.3
Electrical conductivity (dS/m)	1.2	4.5
CEC (c mol kg^{-1})	45	33
Colwell P (mg/kg)	23	37
Clay (<2 µm, %)	-	50.9
Silt (2-60 µm, %)	-	11.3
Sand (60-2000 µm, %)	-	37.8
Soil texture	Clay	Clay
NH_4^+-N (mg kg^{-1})	0.95	3.3
NO_3^- -N (mg kg^{-1})	7.2	270

Table S3. Application rates of the various nitrification inhibitor treatments in mol % of $(NH_4)_2SO_4$-N and weight % of total $(NH_4)_2SO_4$ applied. All samples were treated with the $(NH_4)_2SO_4$ at a rate of 100 mg N kg^{-1} soil.

		Application rates	
Treatment	mol % of $(NH_4)_2SO_4$-N	mg kg^{-1} soil	weight % of total $(NH_4)_2SO_4$
L-DMPP[a]	1.5	13.9 *[10.3]*	2.95 *[2.18]*
M-DMPP[a]	3.6	49.9 *[24.7]*	10.6 *[5.23]*
H-DMPP[a]	10	138.7 *[68.6]*	29.4 *[14.5]*
N001	10	139.1	29.5
N002	10	129.1	27.4
N003	10	150.5	31.9
N004	10	130.5	27.7
N005	10	170.5	36.1
N006	10	129.8	27.5
N007	10	225.3	47.8
N008	10	285.2	60.5
N009	10	173.3	36.7
N010	10	233.2	49.4
N011	10	213.2	45.2
N012	10	153.3	32.5
N013	10	119.2	25.3
N014	10	130.6	27.7
N015	10	120.6	25.6
N016	10	109.2	23.2
N017	10	116.3	24.7

[a] Values (mg kg^{-1} soil, weight % of total $(NH_4)_2SO_4$) for the DMPP treatments are given for the salt 3,4-dimethylpyrazole phosphate. Values for the active core 3,4-dimethylpyrazole (*i.e.*, DMP) are included inside square brackets.

Table S4. Soil incubation tests conducted in Horsham soil (pH 8.8). All samples were treated with the fertilizer $(NH_4)_2SO_4$ at a rate of 100 mg N kg^{-1}.[a]

Treatment	[NH_4^+-N] (mg kg^{-1} soil)[b],[c],[d]						[NO_x^--N] (mg kg^{-1} soil)[b],[c],[d]					
	Day 0	Day 3	Day 7	Day 14	Day 21	Day 28	Day 0	Day 3	Day 7	Day 14	Day 21	Day 28
Soil incubation at 25°C.												
Experiment set 1 (WFPS = 52%)												
$(NH_4)_2SO_4$-control	86.8 ± 1.5	68.9 ± 4.6	60.4 ± 3.3	24.6 ± 6.3	-	17.2 ± 14.1	6.39 ± 0.36	14.7 ± 2.3	32.7 ± 3.8	66.1 ± 9.9	-	73.3 ± 16.0
L-DMPP	81.0 ± 1.7	76.6 ± 0.22	72.1 ± 3.0	53.4 ± 3.3	-	22.4 ± 10.4	5.35 ± 0.27	16.2 ± 0.38	23.7 ± 0.49	38.2 ± 3.3*	-	76.3 ± 11.0
N001	82.2 ± 1.6	74.7 ± 1.3	61.0 ± 3.8	36.1 ± 1.9	-	13.8 ± 5.7	5.47 ± 0.33	16.3 ± 0.63	30.9 ± 1.0	61.5 ± 0.60	-	84.8 ± 7.8
N002	82.3 ± 1.8	77.6 ± 1.9	72.9 ± 0.50	57.4 ± 0.88*	-	55.1 ± 21.8**,#	5.69 ± 0.33	11.8 ± 1.0	23.9 ± 0.13	38.1 ± 1.2*	-	57.2 ± 12.6
N003	87.1 ± 0.52	78.0 ± 0.70	67.4 ± 1.1	42.8 ± 1.1	-	10.8 ± 7.2	5.91 ± 0.17	17.6 ± 0.37	31.1 ± 0.92	48.5 ± 5.4	-	84.6 ± 3.2
N004	79.7 ± 0.31	69.4 ± 3.8	70.1 ± 3.6	50.5 ± 9.5	-	0 ± 0	5.61 ± 0.05	15.8 ± 2.0	23.5 ± 3.9	43.1 ± 10.1	-	105.4 ± 0.70*,#
Experiment set 2 (WFPS = 61%)												
$(NH_4)_2SO_4$-control	91.8 ± 0.96	79.8 ± 3.5	74.4 ± 3.2	24.9 ± 12.7	24.5 ± 14.2	0 ± 0	15.7 ± 0.35	22.9 ± 3.5	38.5 ± 3.9	87.1 ± 16.4	88.3 ± 16.4	121.1 ± 0.07
M-DMPP	86.2 ± 4.8	84.0 ± 0.11	85.2 ± 0.08	72.8 ± 1.3*	54.5 ± 2.3	33.8 ± 1.0	14.3 ± 0.88	19.2 ± 0.28	24.5 ± 0.35	35.9 ± 1.4	55.9 ± 2.4	77.7 ± 1.5
N005	83.2 ± 0.78	79.7 ± 0.80	77.7 ± 1.5	61.8 ± 8.8	48.5 ± 12.0	41.8 ± 17.9	15.2 ± 0.05	18.4 ± 1.7	22.7 ± 2.6	40.8 ± 13.9	64.9 ± 13.8	69.2 ± 23.2
N006	83.8 ± 3.0	78.5 ± 0.91	71.7 ± 8.2	50.1 ± 11.9	63.2 ± 13.9	45.4 ± 7.5	15.3 ± 0.27	22.7 ± 1.1	34.0 ± 11.8	66.5 ± 15.3	55.6 ± 21.9	73.5 ± 10.3
N007	83.0 ± 1.2	78.8 ± 1.7	45.4 ± 5.0	18.7 ± 7.6##	21.6 ± 16.7	3.66 ± 1.8	14.7 ± 0.04	21.1 ± 1.9	66.1 ± 4.9	91.0 ± 9.1	97.1 ± 18.3	115.2 ± 1.7
N008	82.6 ± 1.6	77.9 ± 0.14	59.7 ± 1.1	39.5 ± 10.4	0 ± 0##	12.0 ± 9.8	14.8 ± 0.22	21.8 ± 0.40	49.6 ± 2.1	70.6 ± 11.9	117.3 ± 0.40#	101.8 ± 10.7

N009	87.5 ± 3.0	72.7 ± 3.4	42.7 ± 3.8	21.5 ± 17.6$^{\#}$	4.02 ± 3.3$^{\#}$	21.9 ± 17.9	16.6 ± 0.71	21.0 ± 2.5	65.6 ± 4.7	88.2 ± 21.6	110.6 ± 3.6	90.4 ± 23.1
N010	87.2 ± 1.9	74.2 ± 2.2	60.4 ± 4.3	24.0 ± 19.6$^{\#}$	19.3 ± 15.7	0.73 ± 0.60	16.2 ± 0.52	19.6 ± 2.5	46.1 ± 3.4	79.9 ± 24.0	85.4 ± 19.0	115.0 ± 0.48
N011	86.0 ± 1.5	74.8 ± 1.4	49.0 ± 2.5	21.8 ± 17.8$^{\#}$	3.04 ± 2.5$^{\#}$	13.2 ± 10.8	15.3 ± 0.18	21.3 ± 0.93	56.4 ± 1.9	84.3 ± 19.9	108.1 ± 2.0	97.7 ± 15.4
N012	85.8 ± 1.1	76.4 ± 0.77	56.1 ± 1.9	25.2 ± 18.3$^{\#}$	2.05 ± 0.8$^{\#}$	2.77 ± 1.2	15.2 ± 0.34	24.9 ± 0.62	54.1 ± 0.25	85.6 ± 20.3	116.6 ± 1.0	117.2 ± 0.55
Experiment set 3 (WFPS = 52%)												
$(NH_4)_2SO_4$-control	83.7 ± 1.6	72.4 ± 6.8	26.4 ± 0.66	0 ± 0	0 ± 0	0 ± 0	8.36 ± 0.02	23.0 ± 4.3	66.4 ± 10.6	103.6 ± 0.84	105.9 ± 0.37	108.6 ± 0.95
H-DMPP	81.9 ± 2.2	87.6 ± 0.79	83.6 ± 1.5***	80.8 ± 0.33***	70.8 ± 4.8***	74.6 ± 1.5***	7.39 ± 0.45	13.5 ± 0.14	15.7 ± 0.90***	20.7 ± 1.2***	21.2 ± 2.1***	29.7 ± 0.35***
N002	99.0 ± 6.5	86.9 ± 5.5	81.3 ± 2.0***	55.0 ± 1.0$^{***,\#\#\#}$	33.0 ± 4.0$^{***,\#\#\#}$	9.50 ± 3.9$^{\#\#\#}$	9.78 ± 1.4	13.5 ± 1.9	28.5 ± 0.55***	49.8 ± 1.2$^{***,\#\#\#}$	82.2 ± 5.8$^{**,\#\#\#}$	105.5 ± 2.3$^{\#\#\#}$
N013	101.8 ± 3.7$^{*,\#}$	83.7 ± 1.1	77.9 ± 3.9***	61.0 ± 1.8$^{***,\#}$	53.7 ± 1.4***	28.3 ± 1.6$^{***,\#\#\#}$	9.44 ± 0.86	14.0 ± 0.29	24.4 ± 2.4***	41.3 ± 2.2$^{***,\#\#}$	58.2 ± 2.2$^{***,\#\#\#}$	88.5 ± 1.5$^{*,\#\#\#}$
N014	101.1 ± 2.3$^{\#}$	77.1 ± 1.9	62.0 ± 4.8$^{***,\#\#}$	15.9 ± 4.8$^{\#\#\#}$	6.64 ± 5.4$^{\#\#\#}$	0 ± 0$^{\#\#\#}$	9.63 ± 0.31	16.3 ± 1.7	39.4 ± 5.6$^{***,\#\#}$	91.8 ± 6.1$^{\#\#\#}$	108.9 ± 7.5$^{\#\#\#}$	119.9 ± 0.90$^{\#\#\#}$
N015	96.7 ± 2.4	79.6 ± 1.3	50.9 ± 0.89$^{**,\#\#\#}$	10.7 ± 2.1$^{\#\#\#}$	14.5 ± 11.8$^{\#\#\#}$	0 ± 0$^{\#\#\#}$	9.50 ± 0.86	21.9 ± 0.74	51.5 ± 2.6$^{***,\#\#\#}$	98.7 ± 1.4$^{\#\#\#}$	96.8 ± 11.4$^{\#\#\#}$	115.9 ± 1.5$^{\#\#\#}$
N016	99.3 ± 2.7	90.5 ± 2.1*	74.6 ± 1.1***	55.4 ± 1.5$^{***,\#\#\#}$	42.2 ± 1.3$^{***,\#\#\#}$	28.9 ± 2.1$^{***,\#\#\#}$	8.15 ± 1.1	16.6 ± 0.35	30.3 ± 0.30***	52.6 ± 1.3$^{***,\#\#\#}$	69.3 ± 0.72$^{***,\#\#\#}$	84.8 ± 2.0$^{**,\#\#\#}$
N017	99.0 ± 4.2	85.7 ± 1.8	68.8 ± 3.1***	64.2 ± 2.3***	55.6 ± 3.2***	33.9 ± 0.59$^{***,\#\#\#}$	9.28 ± 0.59	15.2 ± 0.57	19.7 ± 1.9***	38.8 ± 1.9$^{***,\#}$	51.1 ± 4.0$^{***,\#\#\#}$	80.4 ± 0.45$^{***,\#\#\#}$
Soil incubation at 35°C.												
$(NH_4)_2SO_4$-control	100.9 ± 1.3	82.0 ± 2.0	54.6 ± 0.80	18.5 ± 3.4	15.7 ± 10.9	2.04 ± 0.85	8.05 ± 0.18	30.6 ± 1.5	46.4 ± 3.9	98.5 ± 3.6	99.8 ± 15.6	123.0 ± 2.0
H-DMPP	101.5 ± 0.64	93.4 ± 2.9	82.3 ± 2.8	68.7 ± 0.89***	54.2 ± 3.8**	36.0 ± 10.7**	11.5 ± 1.1	14.9 ± 0.30	25.3 ± 0.93	42.9 ± 1.9***	52.1 ± 10.4**	86.4 ± 14.3*
N002	91.8 ± 4.1	94.9 ± 3.6	83.7 ± 2.9*	54.8 ± 2.3**	42.4 ± 7.2	26.1 ± 20.4	9.07 ± 0.62	20.0 ± 0.45	38.5 ± 1.8	67.0 ± 1.7	83.7 ± 7.3	96.5 ± 22.7

N013	96.9 ± 4.1	93.5 ± 4.7	78.8 ± 3.3	50.4 ± 2.4*	39.4 ± 8.7	37.6 ± 12.9**	8.03 ± 0.74	26.1 ± 1.3	38.6 ± 4.0	65.5 ± 3.4	83.1 ± 9.5	86.5 ± 19.7*
N014	95.6 ± 4.3	83.0 ± 2.7	80.6 ± 3.2	57.5 ± 4.3**	15.8 ± 4.0##	19.4 ± 7.1	7.90 ± 0.61	20.3 ± 1.1	30.6 ± 3.0	67.0 ± 4.1	104.9 ± 9.8###	113.9 ± 8.2
N015	97.5 ± 4.3	82.0 ± 0.4	61.0 ± 2.6	22.4 ± 2.5[c]	6.00 ± 4.2###	0 ± 0##	9.06 ± 0.51	29.0 ± 1.7	59.5 ± 5.0	97.1 ± 2.4###	112.3 ± 6.0###	129.4 ± 2.8##
N016	96.0 ± 4.0	93.6 ± 1.4	74.6 ± 0.55	55.4 ± 0.18**	24.1 ± 2.9#	15.7 ± 2.6	10.2 ± 0.97	22.1 ± 1.3	33.2 ± 3.2	68.6 ± 0.6	103.8 ± 2.6###	119.6 ± 3.7
N017	89.3 ± 1.4	90.0 ± 2.7	81.8 ± 2.0	56.1 ± 1.5**	21.7 ± 4.5#	21.3 ± 0.76	8.1 ± 0.72	22.6 ± 1.3	40.5 ± 2.3	67.8 ± 3.2	106.9 ± 6.3###	111.8 ± 2.0

[a] Application rates are in mol% of applied fertilizer N; for N001 - N017 = 10, L-DMPP = 1.5, M-DMPP = 3.6, H-DMPP = 10. [b] Mean values (n = 3); errors are standard errors of the mean. [c] Statistical significance: $P < .05$ (*), $P < .01$ (**), $P < .001$ (***) respectively, when comparing inhibitor treatments to the control treatment with $(NH_4)_2SO_4$ alone. [d] Statistical significance: $P < .05$ (#), $P < .01$ (##), $P < .001$ (###) respectively, when comparing new inhibitor treatments to DMPP treatment.

Statistical analyses were performed on raw NH_4^+-N and NO_x^--N data in R (version 3.5.2)[1], using the statistical package *emmeans*[2]. Data were assessed for statistical significance ($P < 0.05$) via two-way analysis of variation (ANOVA)[3] assessing the impact of the two factors “Day” and “Treatment”, and pair-wise comparisons between treatments at each time point were evaluated using a TukeyHSD post-hoc adjustment.

Table S5. Soil incubation tests conducted in Dahlen soil (pH 7.3). All samples were treated with the fertilizer $(NH_4)_2SO_4$ at a rate of 100 mg N kg^{-1}.[a]

Treatment	[NH_4^+-N] (mg kg^{-1} soil)[b],[c],[d]						[NO_x^--N] (mg kg^{-1} soil)[b],[c],[d]					
	Day 0	Day 3	Day 7	Day 14	Day 21	Day 28	Day 0	Day 3	Day 7	Day 14	Day 21	Day 28
Soil incubation at 25°C.												
Experiment set 1 (WFPS = 52%)												
$(NH_4)_2SO_4$-control	176.8 ± 2.9	199.2 ± 4.4	168.5 ± 5.8	63.8 ± 8.4	67.5 ± 46.2	0 ± 0	281.0 ± 14.8	326.8 ± 10.7	290.5 ± 10.1	428.0 ± 10.3	401.6 ± 57.9	567.8 ± 18.3
M-DMPP	176.6 ± 4.9	187.7 ± 2.4	188.1 ± 3.7	189.1 ± 7.4***	186.7 ± 4.4**	197.8 ± 9.2***	247.6 ± 8.9	270.3 ± 10.0	270.1 ± 9.9	254.0 ± 13.6***	284.7 ± 7.0	293.6 ± 19.2***
N001	164.8 ± 3.0	174.0 ± 4.7	161.5 ± 10.5	166.1 ± 2.4*	143.3 ± 31.5	59.7 ± 39.5###	271.9 ± 7.0	275.8 ± 5.6	241.0 ± 29.5	298.6 ± 2.6*	251.1 ± 25.8*	480.7 ± 33.2###
N002	167.3 ± 1.2	175.7 ± 1.0	178.1 ± 12.7	184.2 ± 1.9**	186.2 ± 1.7**	174.2 ± 3.3***	268.0 ± 2.9	252.3 ± 13.3	268.2 ± 25.0	281.7 ± 4.2**	293.0 ± 3.4	328.6 ± 15.9***
N003	180.4 ± 0.75	179.6 ± 6.8	176.9 ± 3.3	170.6 ± 7.0**	88.0 ± 5.3#	72.0 ± 52.5###	266.5 ± 2.9	280.5 ± 9.5	276.0 ± 3.5	304.0 ± 6.3	398.6 ± 10.8	455.9 ± 58.1##
N004	176.7 ± 3.3	180.1 ± 5.6	179.6 ± 2.1	172.8 ± 10.9**	67.2 ± 8.1#	18.0 ± 14.7###	274.1 ± 4.0	265.8 ± 3.8	269.3 ± 2.1	289.2 ± 13.6*	403.9 ± 9.9	564.2 ± 50.7###
N005	177.8 ± 5.5	172.6 ± 8.1	198.6 ± 16.5	110.9 ± 7.7	88.5 ± 43.3#	0 ± 0###	274.2 ± 1.7	268.4 ± 8.3	306.3 ± 6.7	366.2 ± 6.6	424.9 ± 58.9#	555.0 ± 13.2###
N006	175.9 ± 5.1	188.1 ± 4.0	191.8 ± 11.1	181.0 ± 1.7**	153.3 ± 17.3	118.9 ± 30.9**	257.1 ± 3.4	276.5 ± 0.98	300.4 ± 10.3	282.0 ± 1.6**	318.3 ± 25.0	384.9 ± 25.8***
N007	185.3 ± 1.8	186.0 ± 4.4	192.5 ± 6.0	130.6 ± 26.6	41.6 ± 34.0###	64.0 ± 52.3###	250.9 ± 4.3	269.5 ± 6.7	321.9 ± 9.3	351.2 ± 31.4	443.7 ± 33.8##	455.1 ± 75.1##
N008	188.1 ± 0.68	191.6 ± 3.8	201.6 ± 5.4	91.0 ± 4.5#	25.2 ± 20.6###	0 ± 0###	256.5 ± 4.1	271.6 ± 4.5	320.2 ± 12.7	403.7 ± 6.6##	502.7 ± 21.1###	554.8 ± 3.5###
N009	209.6 ± 21.1	188.7 ± 13.8	164.9 ± 7.5	82.0 ± 6.5##	8.10 ± 6.6###	0 ± 0###	293.0 ± 23.8	254.3 ± 37.4	295.1 ± 5.0	390.0 ± 5.2#	503.1 ± 16.8###	538.6 ± 20.4###
N010	176.8 ± 0.96	195.3 ± 2.6	182.7 ± 4.5	108.3 ± 8.3	3.87 ± 3.2###	0 ± 0###	257.2 ± 4.7	270.5 ± 6.9	296.9 ± 13.1	366.9 ± 9.2	508.0 ± 8.5###	522.4 ± 9.1###
N011	201.3 ± 15.5	192.4 ± 9.2	167.1 ± 4.4	126.7 ± 17.6	13.5 ± 11.0###	0 ± 0###	280.2 ± 18.2	255.3 ± 24.4	294.8 ± 2.9	327.6 ± 8.6	366.3 ± 39.3	567.3 ± 42.4###

Experiment set 2 (WFPS = 52%)												
$(NH_4)_2SO_4$-control	96.8 ± 1.5	85.5 ± 5.3	73.3 ± 0.48	18.2 ± 2.5	0 ± 0	0 ± 0	312.3 ± 3.1	269.8 ± 43.3	345.4 ± 4.2	345.2 ± 26.0	406.7 ± 27.5	442.7 ± 3.0
L-DMPP	97.0 ± 0.10	89.2 ± 1.1	89.3 ± 3.3	79.4 ± 1.7^{***}	64.9 ± 0.54^{***}	73.3 ± 2.4^{***}	308.4 ± 1.2	302.2 ± 6.0	291.8 ± 26.9	304.6 ± 10.0	332.8 ± 5.9	352.8 ± 4.6^{*}
N012	95.8 ± 2.3	79.1 ± 1.3	74.8 ± 0.63	40.2 ± $11.6^{\#\#}$	0 ± $0^{\#\#\#}$	8.02 ± $6.6^{\#\#\#}$	312.3 ± 5.7	281.1 ± 14.6	354.7 ± 5.2	364.7 ± 13.4	432.6 ± $18.1^{\#\#}$	423.7 ± 13.7
N013	90.8 ± 0.25	93.0 ± 1.6	84.6 ± 7.3	81.5 ± 2.2^{***}	72.1 ± 2.1^{***}	81.3 ± 1.5^{***}	303.7 ± 2.3	332.0 ± 6.8	338.8 ± 5.6	289.6 ± 14.9	322.0 ± 2.7^{*}	342.9 ± 1.1^{**}
N014	95.2 ± 0.55	91.9 ± 3.4	89.9 ± 1.4	76.1 ± 1.6^{***}	54.1 ± 1.0^{***}	60.1 ± 16.3^{***}	301.6 ± 1.7	292.7 ± 20.4	324.7 ± 3.6	293.5 ± 15.4	346.6 ± 12.4	374.2 ± 13.6
N015	93.4 ± 0.38	87.8 ± 0.75	82.0 ± 2.0	43.3 ± $15.4^{\#\#}$	18.4 ± $15.0^{\#\#\#}$	0 ± $0^{\#\#\#}$	303.1 ± 0.71	310.6 ± 1.6	323.4 ± 6.1	345.8 ± 43.3	399.0 ± 24.0	450.1 ± $9.1^{\#\#}$
Experiment set 3 (WFPS = 52%)												
$(NH_4)_2SO_4$-control	88.0 ± 3.3	81.6 ± 0.88	70.8 ± 4.3	51.8 ± 0.52	42.3 ± 0.35	14.4 ± 0.61	280.8 ± 22.4	336.0 ± 5.6	328.4 ± 29.0	383.8 ± 7.7	416.8 ± 2.4	444.8 ± 2.6
H-DMPP	93.9 ± 2.7	91.9 ± 2.3	82.9 ± 1.3^{*}	77.7 ± 4.0^{***}	76.8 ± 1.0^{***}	76.5 ± 1.4^{***}	338.7 ± 12.7	356.1 ± 3.2	362.2 ± 4.6	357.3 ± 5.1	375.6 ± 3.7	392.9 ± 6.5
N002	94.9 ± 7.8	87.8 ± 1.3	74.8 ± 1.1	75.6 ± 2.0^{***}	63.7 ± $2.1^{***,\#}$	59.2 ± $0.82^{***,\#\#\#}$	317.3 ± 28.1	358.5 ± 4.1	366.9 ± 3.3	349.0 ± 18.5	372.8 ± 12.5	418.1 ± 2.4
N013	93.3 ± 0.13	83.4 ± 0.76	72.6 ± 0.79	70.0 ± 1.2^{***}	62.8 ± $0.17^{***,\#}$	54.6 ± $1.2^{***,\#\#\#}$	351.6 ± 1.2^{**}	358.3 ± 2.0	341.7 ± 22.3	343.6 ± 19.1	394.4 ± 2.9	422.1 ± 1.6
N016	89.2 ± 0.72	81.3 ± 0.77	75.2 ± 0.96	74.6 ± 2.0^{***}	65.2 ± 0.41^{***}	55.9 ± $1.1^{***,\#\#\#}$	331.2 ± 10.4	359.7 ± 3.0	364.6 ± 1.6	343.6 ± 19.1	394.8 ± 2.7	429.6 ± 3.9
N017	86.0 ± 2.7	83.4 ± 0.32	77.0 ± 0.68	75.5 ± 5.4^{***}	66.9 ± 2.0^{***}	54.6 ± $2.5^{***,\#\#\#}$	299.9 ± 22.3	351.5 ± 1.7	365.4 ± 2.3	388.3 ± 1.1	406.8 ± 3.0	431.4 ± 2.6
Soil incubation at 35°C.												
$(NH_4)_2SO_4$-control	94.4 ± 3.1	90.6 ± 3.2	77.8 ± 2.6	60.3 ± 3.0	40.7 ± 3.2	39.3 ± 8.0	325.3 ± 24.9	375.6 ± 2.9	326.1 ± 31.6	374.6 ± 32.6	408.7 ± 5.0	429.5 ± 4.3
H-DMPP	95.8 ± 2.3	93.5 ± 1.6	72.0 ± 1.0	71.3 ± 2.7	49.5 ± 5.6	37.9 ± 7.6	344.3 ± 14.7	366.2 ± 4.0	253.3 ± 51.3	390.8 ± 5.1	388.5 ± 1.4	401.9 ± 2.6
N002	96.3 ± 0.79	94.7 ± 3.1	97.6 ± $1.6^{**,\#\#\#}$	94.3 ± $2.1^{***,\#\#\#}$	67.8 ± $3.3^{***,\#\#}$	74.7 ± $1.2^{***,\#\#\#}$	351.2 ± 2.0	312.0 ± 35.1	373.7 ± $5.9^{\#\#}$	395.5 ± 2.0	297.7 ± $44.2^{**,\#}$	405.7 ± 2.7

N013	97.6 ± 1.0	98.8 ± 0.20	94.2 ± $0.74^{*,\#\#\#}$	88.7 ± $0.24^{***,\#}$	64.3 ± $2.1^{***,\#}$	64.8 ± $0.67^{***,\#\#\#}$	349.9 ± 8.6	371.6 ± 1.4	377.4 ± 3.5^{c}	403.7 ± 1.5	354.1 ± 28.8	418.0 ± 4.3
N016	101.7 ± 0.88	100.2 ± 1.1	95.6 ± $1.2^{**,\#\#\#}$	85.3 ± 4.4^{***}	71.4 ± $0.75^{***,\#\#\#}$	84.5 ± $2.4^{***,\#\#\#}$	360.5 ± 3.1	369.9 ± 2.2	374.2 ± $1.7^{\#\#}$	384.1 ± 6.5	392.5 ± 2.8	411.4 ± 1.9
N017	104.2 ± 1.7	97.6 ± 1.6	95.0 ± $0.96^{*,\#\#\#}$	85.8 ± 2.1^{***}	64.6 ± $2.3^{***,\#}$	65.1 ± $2.7^{***,\#\#\#}$	358.5 ± 4.6	368.8 ± 3.4	381.9 ± $3.9^{\#\#\#}$	378.6 ± 10.3	391.7 ± 4.0	421.8 ± 1.9

[a] Application rates are in mol% of applied fertilizer N; for N001 - N017 = 10, L-DMPP = 1.5, M-DMPP = 3.6, H-DMPP = 10. [b] Mean values (n = 3); errors are standard errors of the mean. [c] Statistical significance: *P* < .05 (*), *P* < .01 (**), *P* < .001 (***) respectively, when comparing inhibitor treatments to the control treatment with $(NH_4)_2SO_4$ alone. [d] Statistical significance: *P* < .05 ($^{\#}$), *P* < .01 ($^{\#\#}$), *P* < .001 ($^{\#\#\#}$) respectively, when comparing new inhibitor treatments to DMPP treatment.

Statistical analyses were performed on raw NH_4^+-N and NO_x^--N data in R (version 3.5.2)[1], using the statistical package *emmeans*[2]. Data were assessed for statistical significance (*P* < 0.05) via two-way analysis of variation (ANOVA)[3] assessing the impact of the two factors “Day” and “Treatment”, and pair-wise comparisons between treatments at each time point were evaluated using a TukeyHSD post-hoc adjustment.

Table S6. Ammonium loss (%) during a 28-day incubation in Horsham soil (pH 8.8). All samples were treated with the fertilizer $(NH_4)_2SO_4$ at a rate of 100 mg N kg^{-1}.[a],[b],[c]

Treatment	NH_4^+-N loss (%)				
	Day 3	Day 7	Day 14	Day 21	Day 28
Soil incubation at 25°C.					
Experiment set 1					
$(NH_4)_2SO_4$-control	20.7 ± 5.6	30.4 ± 4.2	71.6 ± 7.5	-	80.2 ± 16.4
L-DMPP	5.5 ± 2.2	11.1 ± 4.2	34.1 ± 4.7	-	72.3 ± 13.1
N001	9.2 ± 2.6	25.8 ± 5.1	56.1 ± 3.2	-	83.3 ± 7.4
N002	5.7 ± 3.2	11.4 ± 2.3	30.2 ± 2.5	-	33.0 ± 26.6
N003	10.4 ± 1.0	22.6 ± 1.4	50.8 ± 1.4	-	87.6 ± 8.3
N004	12.9 ± 4.8	12.0 ± 4.6	36.6 ± 11.9	-	100 ± 0.5
Experiment set 2					
$(NH_4)_2SO_4$-control	13.1 ± 4.0	19.0 ± 3.7	72.9 ± 13.9	73.4 ± 15.5	100 ± 1.5
M-DMPP	2.5 ± 5.5	1.1 ± 5.5	15.5 ± 5.8	36.8 ± 6.5	60.8 ± 6.6
N005	4.2 ± 1.3	6.7 ± 2.0	25.7 ± 10.6	41.7 ± 14.4	49.8 ± 21.5
N006	6.5 ± 3.7	14.4 ± 10.4	40.1 ± 14.7	24.5 ± 17.0	45.8 ± 9.7
N007	5.1 ± 2.5	45.3 ± 6.2	77.4 ± 9.4	74.0 ± 20.2	95.6 ± 3.0
N008	5.7 ± 2.0	27.7 ± 2.5	52.2 ± 12.8	100 ± 2.8	85.5 ± 12.1
N009	16.9 ± 5.2	51.2 ± 5.9	75.4 ± 20.5	95.4 ± 6.1	74.9 ± 20.9
N010	14.9 ± 3.3	30.7 ± 5.4	72.5 ± 22.6	77.9 ± 18.3	99.2 ± 3.1
N011	13.0 ± 2.4	43.1 ± 3.5	74.7 ± 20.8	96.5 ± 3.8	84.7 ± 12.7
N012	10.9 ± 1.6	34.6 ± 2.6	70.6 ± 21.4	97.6 ± 2.0	96.8 ± 2.2
Experiment set 3					
$(NH_4)_2SO_4$-control	13.5 ± 8.4	68.5 ± 2.4	100 ± 2.7	100 ± 2.7	100 ± 2.7
H-DMPP	-6.9 ± 2.8	-2.1 ± 3.2	1.4 ± 2.7	13.6 ± 6.4	8.9 ± 3.2
N002	12.2 ± 8.6	17.9 ± 6.9	44.5 ± 7.2	66.6 ± 8.9	90.4 ± 9.7
N013	17.7 ± 3.9	23.4 ± 5.4	40.0 ± 4.3	47.2 ± 4.3	72.2 ± 4.8
N014	23.8 ± 3.0	38.7 ± 5.3	84.3 ± 5.6	93.4 ± 6.2	100 ± 3.2
N015	17.6 ± 2.9	47.4 ± 2.9	88.9 ± 4.0	85.0 ± 12.7	100 ± 3.6
N016	8.9 ± 3.4	24.9 ± 3.0	44.2 ± 3.3	57.5 ± 3.4	70.9 ± 3.9
N017	13.5 ± 4.7	30.6 ± 5.5	35.1 ± 5.1	43.9 ± 5.7	65.8 ± 5.2
Soil incubation at 35°C.					
$(NH_4)_2SO_4$-control	18.8 ± 2.4	45.9 ± 1.7	81.6 ± 3.7	84.5 ± 11.0	98.0 ± 2.0
H-DMPP	7.9 ± 3.0	18.9 ± 2.8	32.3 ± 4.1	46.6 ± 5.9	64.5 ± 12.7
N002	-3.3 ± 5.9	8.8 ± 5.5	40.3 ± 5.4	53.8 ± 9.4	71.6 ± 22.9
N013	3.5 ± 6.4	18.7 ± 5.5	48.0 ± 5.3	59.3 ± 10.3	61.2 ± 14.2
N014	13.2 ± 5.4	15.7 ± 5.7	39.8 ± 6.6	83.5 ± 7.2	79.7 ± 9.4
N015	15.9 ± 4.5	37.4 ± 5.4	77.0 ± 6.2	93.8 ± 7.5	100 ± 6.3
N016	2.5 ± 4.5	22.3 ± 4.3	42.3 ± 4.6	74.9 ± 6.1	83.7 ± 6.1
N017	-0.8 ± 3.4	8.5 ± 2.8	37.2 ± 2.3	75.7 ± 5.4	76.2 ± 2.1

[a] Application rates are in mol% of applied fertilizer N; for N001 - N017 = 10, L-DMPP = 1.5, M-DMPP = 3.6, H-DMPP = 10. [b] NH_4^+-N loss values calculated from NH_4^+-N concentrations detected in samples at each timepoint. [c] Mean values (n = 3); errors are standard errors of the mean.

Table S7. NO_x^--N production rates after a 28-day incubation in Horsham soil (pH 8.8). All samples were treated with the fertilizer $(NH_4)_2SO_4$ at a rate of 100 mg N kg^{-1}.[a],[b],[c]

Treatment	NO_x^--N production rate (mg NO_x^--N/kg soil/day)
Soil incubation at 25°C.	
Experiment set 1	
$(NH_4)_2SO_4$-control	2.4 ± 0.6
L-DMPP	2.5 ± 0.4
N001	2.8 ± 0.3
N002	1.8 ± 0.4
N003	2.8 ± 0.1
N004	3.6 ± 0.02
Experiment set 2	
$(NH_4)_2SO_4$-control	3.8 ± 0.01
M-DMPP	2.3 ± 0.06
N005	1.9 ± 0.8
N006	2.1 ± 0.4
N007	3.6 ± 0.1
N008	3.1 ± 0.4
N009	2.6 ± 0.8
N010	3.5 ± 0.03
N011	2.9 ± 0.6
N012	3.6 ± 0.02
Experiment set 3	
$(NH_4)_2SO_4$-control	3.6 ± 0.03
H-DMPP	0.80 ± 0.02
N002	3.4 ± 0.10
N013	2.8 ± 0.06
N014	3.9 ± 0.03
N015	3.8 ± 0.06
N016	2.7 ± 0.08
N017	2.5 ± 0.03
Soil incubation at 35°C.	
$(NH_4)_2SO_4$-control	4.1 ± 0.07
H-DMPP	2.7 ± 0.5
N002	3.1 ± 0.8
N013	2.8 ± 0.7
N014	3.8 ± 0.3
N015	4.3 ± 0.1
N016	3.9 ± 0.1
N017	3.7 ± 0.08

[a] Application rates are in mol% of applied fertilizer N; for N001 - N017 = 10, L-DMPP = 1.5, M-DMPP = 3.6, H-DMPP = 10. [b] NO_x^--N production rates were calculated from the difference between [NO_x^--N] detected on day 28 and day 0, divided by 28 (*i.e*, duration of soil incubation experiment). [c] Mean values (n = 3); errors are standard errors of the mean.

Table S8. Ammonium loss (%) during a 28-day incubation in Dahlen soil (pH 7.3). All samples were treated with the fertilizer $(NH_4)_2SO_4$ at a rate of 100 mg N kg^{-1}.[a],[b],[c]

Treatment	NH_4^+-N loss (%)				
	Day 3	Day 7	Day 14	Day 21	Day 28
Soil incubation at 25°C.					
Experiment set 1					
$(NH_4)_2SO_4$-control	-12.7 ± 3.0	4.7 ± 3.7	63.9 ± 5.2	61.8 ± 26.2	100 ± 2.4
M-DMPP	-6.3 ± 3.1	-6.5 ± 3.5	-7.0 ± 5.0	-5.7 ± 3.7	-12.0 ± 5.9
N001	-5.6 ± 3.4	2.0 ± 6.6	-0.8 ± 2.3	13.1 ± 18.0	63.8 ± 24.1
N002	-5.0 ± 0.9	-6.4 ± 7.6	-10.1 ± 1.4	-11.3 ± 1.3	-4.1 ± 2.1
N003	0.5 ± 3.8	2.0 ± 1.9	5.5 ± 3.9	51.3 ± 3.0	60.1 ± 29.1
N004	-1.9 ± 3.7	-1.6 ± 2.2	2.2 ± 6.5	62.0 ± 5.1	89.8 ± 8.7
N005	2.9 ± 5.5	-11.7 ± 9.8	37.6 ± 5.4	50.2 ± 24.6	100 ± 4.4
N006	-7.0 ± 3.7	-9.1 ± 7	-2.9 ± 3.0	12.8 ± 10.2	32.4 ± 17.8
N007	-0.3 ± 2.6	-3.9 ± 3.4	29.5 ± 14.4	77.5 ± 18.4	65.4 ± 28.2
N008	-1.9 ± 2.1	-7.2 ± 2.9	51.6 ± 2.4	86.6 ± 11.0	100 ± 0.5
N009	10.0 ± 12.0	21.3 ± 10.9	60.9 ± 12.2	96.1 ± 14.3	100 ± 14.2
N010	-10.4 ± 1.6	-3.3 ± 2.6	38.8 ± 4.7	97.8 ± 1.9	100 ± 0.8
N011	4.4 ± 9.0	17.0 ± 8.1	37.0 ± 12.0	93.3 ± 11.9	100 ± 10.9
Experiment set 2					
$(NH_4)_2SO_4$-control	11.6 ± 5.7	24.2 ± 1.7	81.2 ± 3.2	100 ± 2.2	100 ± 2.2
L-DMPP	8.0 ± 1.1	7.9 ± 3.4	18.2 ± 1.7	33.1 ± 0.6	24.4 ± 2.5
N012	17.5 ± 2.7	22.0 ± 2.5	58.0 ± 12.4	100 ± 3.3	91.6 ± 7.5
N013	-2.5 ± 1.7	6.9 ± 8.1	10.3 ± 2.4	20.6 ± 2.4	10.5 ± 1.6
N014	3.5 ± 3.6	5.6 ± 1.5	20.1 ± 1.8	43.2 ± 1.2	36.8 ± 17.2
N015	5.9 ± 0.9	12.2 ± 2.2	53.6 ± 16.5	80.3 ± 16.1	100 ± 0.6
Experiment set 3					
$(NH_4)_2SO_4$-control	7.3 ± 3.1	19.6 ± 15.2	41.1 ± 10.0	52.0 ± 9.5	83.6 ± 13.3
H-DMPP	2.1 ± 1.6	11.7 ± 6.7	17.3 ± 12.7	18.2 ± 6.6	18.6 ± 4.9
N002	7.5 ± 5.2	21.2 ± 12.2	20.3 ± 21.6	32.9 ± 18.4	37.6 ± 11.0
N013	10.6 ± 3.7	22.2 ± 50.2	24.9 ± 59.3	32.6 ± 2.4	41.5 ± 1.2
N016	8.8 ± 3.3	15.6 ± 5.0	16.4 ± 28.7	26.9 ± 4.6	37.3 ± 4.4
N017	3.0 ± 1.3	10.4 ± 3.6	12.1 ± 3.2	22.2 ± 5.0	36.4 ± 6.8
Soil incubation at 35°C.					
$(NH_4)_2SO_4$-control	4.0 ± 4.7	17.5 ± 4.3	36.1 ± 4.7	56.9 ± 5.0	58.3 ± 9.3
H-DMPP	2.4 ± 3.0	24.8 ± 2.7	25.6 ± 3.8	48.3 ± 6.4	60.5 ± 8.4
N002	1.7 ± 3.3	-1.3 ± 1.9	2.0 ± 2.4	29.6 ± 3.6	22.4 ± 1.5
N013	-1.2 ± 1.1	3.5 ± 1.3	9.2 ± 1.1	34.2 ± 2.4	33.6 ± 1.3
N016	1.5 ± 1.4	6.0 ± 1.5	16.1 ± 4.5	29.8 ± 1.2	16.9 ± 2.6
N017	6.3 ± 2.2	8.8 ± 1.9	17.7 ± 2.6	38.0 ± 2.8	37.6 ± 3.1

[a] Application rates are in mol% of applied fertilizer N; for N001 - N017 = 10, L-DMPP = 1.5, M-DMPP = 3.6, H-DMPP = 10. [b] NH_4^+-N loss calculated from NH_4^+-N concentrations detected in samples at each timepoint. [c] Mean values (n = 3); errors are standard errors of the mean.

The negative values obtained for NH_4^+-N loss (%) for some of the inhibitors, mainly in experiment set 1, could suggest that our treatments may trigger priming effects in the soil (Kuzyakov et al., 2000), affecting the N transformation processes in this soil under the incubation conditions (Mahmood et al., 2017), resulting in a higher concentration of NH_4^+-N later in the experiment than the initial concentration on day 0 (see eqn. 1). This finding will be further explored in future work, for example using isotope tracing techniques, which was outside the scope of this study.

Table S9. NO_x^--N production rates after a 28-day incubation in Dahlen soil (pH 7.3). All samples were treated with the fertilizer $(NH_4)_2SO_4$ at a rate of 100 mg N kg^{-1}.[a],[b],[c]

Treatment	NO_x^--N production rate (mg NO_x^--N/kg soil/day)
Soil incubation at 25°C.	
Experiment set 1	
$(NH_4)_2SO_4$-control	10.2 ± 0.8
M-DMPP	1.6 ± 0.8
N001	7.5 ± 1.2
N002	2.2 ± 0.6
N003	6.8 ± 2.1
N004	10.4 ± 1.8
N005	10.0 ± 0.5
N006	4.6 ± 0.9
N007	7.3 ± 2.7
N008	10.7 ± 0.2
N009	8.8 ± 1.1
N010	9.5 ± 0.4
N011	10.3 ± 1.6
Experiment set 2	
$(NH_4)_2SO_4$-control	4.7 ± 0.2
L-DMPP	1.6 ± 0.2
N012	4.0 ± 0.5
N013	1.4 ± 0.09
N014	2.6 ± 0.5
N015	5.2 ± 0.3
Experiment set 3	
$(NH_4)_2SO_4$-control	5.9 ± 0.8
H-DMPP	1.9 ± 0.5
N002	3.6 ± 1.0
N013	2.5 ± 0.07
N016	3.5 ± 0.4
N017	4.7 ± 0.8
Soil incubation at 35°C.	
$(NH_4)_2SO_4$-control	3.7 ± 0.9
H-DMPP	2.1 ± 0.5
N002	1.9 ± 0.1
N013	2.4 ± 0.3
N016	1.8 ± 0.1
N017	2.3 ± 0.2

[a] Application rates are in mol% of applied fertilizer N; for N001 - N017 = 10, L-DMPP = 1.5, M-DMPP = 3.6, H-DMPP = 10. [b] NO_x^--N production rates were calculated from the difference between [NO_x^--N] detected on day 28 and day 0, divided by 28 (*i.e.*, duration of soil incubation experiment). [c] Mean values (n = 3); errors are standard errors of the mean.

Table S10. Nitrification inhibition (%) during a 28-day incubation in Horsham soil (pH 8.8). All samples were treated with the fertilizer $(NH_4)_2SO_4$ at a rate of 100 mg N kg^{-1}.[a],[b],[c]

Treatment	Nitrification Inhibition (NH_4^+-N) / % Day 3	Day 7	Day 14	Day 21	Day 28	(NO_x^--N) / % Day 28
Soil incubation at 25°C.						
Experiment set 1						
L-DMPP	73.4 ± 35.0	63.5 ± 21.6	52.3 ± 13.7	-	9.7 ± 26.2	-6.1 ± 29.0
N001	55.6 ± 33.2	15.1 ± 21.9	21.7 ± 11.7	-	-3.9 ± 22.4	-18.7 ± 27.0
N002	72.4 ± 36.7	62.4 ± 18.1	57.8 ± 12.7	-	58.8 ± 40.7	23.0 ± 30.9
N003	49.7 ± 30.5	25.9 ± 15.1	29.1 ± 11.1	-	-9.4 ± 23.0	-17.6 ± 24.7
N004	37.4 ± 37.0	60.5 ± 22.2	48.8 ± 20.4	-	-24.8 ± 21.0	-49.2 ± 26.7
Experiment set 2						
M-DMPP	80.6 ± 61.6	94.2 ± 50.2	78.7 ± 111.1	49.8 ± 72.8	39.2 ± 8.5	39.9 ± 1.7
N005	67.8 ± 119.1	64.8 ± 72.6	64.8 ± 54.8	43.2 ± 39.4	50.2 ± 21.6	48.7 ± 22.0
N006	50.8 ± 51.8	23.8 ± 58.4	44.9 ± 37.1	66.6 ± 47.1	54.2 ± 10.9	44.8 ± 9.8
N007	61.0 ± 66.2	-139.2 ± 60.8	-6.2 ± 23.7	-0.8 ± 34.7	4.4 ± 3.6	4.5 ± 1.7
N008	56.6 ± 73.0	-46.3 ± 46.2	28.4 ± 31.5	-36.3 ± 117.8	14.5 ± 12.2	17.5 ± 10.2
N009	-28.9 ± 51.4	-170.1 ± 71.4	-3.5 ± 34.1	-30.0 ± 49.7	25.1 ± 21.0	30.0 ± 21.9
N010	-13.8 ± 40.4	-62.2 ± 42.3	0.6 ± 36.5	-6.2 ± 32.8	0.8 ± 3.5	6.2 ± 0.75
N011	0.9 ± 35.4	-127.1 ± 81.7	-2.4 ± 34.4	-31.5 ± 77.6	15.3 ± 12.9	21.8 ± 14.6
N012	16.6 ± 40.5	-82.4 ± 70.7	3.2 ± 35.0	-33.0 ± 146.4	3.2 ± 3.0	3.3 ± 0.71
Experiment set 3						
H-DMPP	151.3 ± 114.3	103.1 ± 6.9	98.6 ± 4.6	86.4 ± 7.3	91.1 ± 4.8	77.8 ± 1.3
N002	9.9 ± 89.0	73.9 ± 11.1	55.5 ± 7.9	33.4 ± 9.3	9.6 ± 10.0	4.5 ± 2.9
N013	-30.9 ± 71.0	65.8 ± 9.0	60.0 ± 5.3	52.8 ± 5.2	27.8 ± 5.6	21.1 ± 2.0
N014	-76.0 ± 81.0	43.5 ± 8.7	15.7 ± 6.2	6.6 ± 6.8	0 ± 0	-9.9 ± 1.4
N015	-30.4 ± 68.3	30.8 ± 5.7	11.1 ± 4.8	15.0 ± 13.0	0 ± 0	-6.1 ± 2.0
N016	34.5 ± 70.3	63.7 ± 6.0	55.8 ± 4.5	42.5 ± 4.4	29.1 ± 4.8	23.6 ± 2.5
N017	0.4 ± 71.0	55.4 ± 8.9	64.9 ± 6.0	56.1 ± 6.4	34.2 ± 5.9	29.0 ± 1.2
Soil incubation at 35°C.						
H-DMPP	57.8 ± 21.5	58.8 ± 7.5	60.4 ± 7.4	44.9 ± 15.8	34.1 ± 13.1	34.9 ± 12.6
N002	117.6 ± 37.3	80.7 ± 12.9	50.6 ± 8.4	36.3 ± 17.7	26.9 ± 23.4	24.0 ± 19.8
N013	81.2 ± 37.8	59.2 ± 12.7	41.2 ± 8.2	29.8 ± 18.2	37.5 ± 14.7	31.7 ± 17.2
N014	29.8 ± 31.5	65.8 ± 13.2	51.2 ± 9.6	1.2 ± 15.5	18.6 ± 9.8	7.8 ± 7.4
N015	15.6 ± 27.6	18.4 ± 12.4	5.7 ± 8.9	-11.1 ± 15.7	-2.1 ± 6.8	-4.7 ± 3.0
N016	86.9 ± 29.1	51.5 ± 10.3	48.2 ± 7.6	11.3 ± 14.9	14.6 ± 6.6	4.8 ± 3.6
N017	104.2 ± 26.0	81.6 ± 7.7	54.4 ± 6.0	10.5 ± 14.5	22.2 ± 3.1	9.8 ± 2.5

[a] Application rates are in mol % of applied fertilizer N; for N001 - N017 = 10, L-DMPP = 1.5, M-DMPP = 3.6, H-DMPP = 10. [b] Calculated at each timepoint from NH_4^+-N loss percentages or NO_x^--N accumulation rates in NI-treated samples compared to the $(NH_4)_2SO_4$-control treatment. [c] Mean values (n = 3); errors are standard errors of the mean.

A note on the inhibition percentages calaculated for the Horsham soil incubation experiments. A discrepancy between the nitrification inhibition calculated based on NH_4^+-N or NO_x^--N was most evident in experiment set 1 in the Horsham soil out of all the reported experiments. Although the trend in inhibitory effectiveness of the treatments was largely conserved between the two measurements, the data for the NO_x^--N based inhibition were considerably lower. Experiment set 1 was conducted six months prior to the other experiments, and it is possible that any microbial activity responsible for background N-transformations had equilibrated or halted by the time subsequent experiments (set 2 and 3) were conducted in this soil.

Negative inhibition values were obtained for some treatments, which could be either due to a greater NH_4^+-N loss at that time-point or a greater accumulation of NO_x^--N by day 28 compared to the control treatment with $(NH_4)_2SO_4$. However, the reasonably good qualitative agreement between the inhibition percentages calculated based on both NH_4^+-N loss and NO_x^--N accumulation suggests that nitrification was likely the major N-transformation process occurring in both soils.

Table S11. Nitrification inhibition (%) during a 28-day incubation in Dahlen soil (pH 7.3). All samples were treated with the fertilizer $(NH_4)_2SO_4$ at a rate of 100 mg N kg^{-1}.[a],[b],[c]

Treatment	Nitrification inhibition (NH_4^+-N) / %					(NO_x^--N) / %
	Day 3	Day 7	Day 14	Day 21	Day 28	Day 28
Soil incubation at 25°C.						
Experiment set 1						
M-DMPP	-50.7 ± 35.9	239.0 ± 217.1	111.0 ± 14.4	109.3 ± 63.1	112.0 ± 6.9	84.0 ± 13.0
N001	-56.2 ± 37.9	57.9 ± 168.1	101.3 ± 12.1	78.8 ± 61.4	36.2 ± 24.2	27.2 ± 14.6
N002	-60.6 ± 28.4	237.4 ± 260.4	115.8 ± 12.5	118.3 ± 65.7	104.1 ± 4.0	78.9 ± 11.9
N003	-103.8 ± 45.1	57.8 ± 99.5	91.4 ± 12.6	17.1 ± 43.3	39.9 ± 29.2	34.0 ± 22.1
N004	-84.7 ± 42.4	134.4 ± 140.4	96.5 ± 15.1	-0.3 ± 43.2	10.2 ± 9.0	-1.2 ± 19.5
N005	-122.9 ± 57.1	350.5 ± 355.1	41.2 ± 12.2	18.8 ± 58.7	0 ± 0	2.1 ± 9.4
N006	-45.0 ± 38.9	293.3 ± 286.1	104.6 ± 12.6	79.3 ± 56.6	67.6 ± 18.1	55.4 ± 13.1
N007	-97.3 ± 38.6	183.2 ± 179.7	53.8 ± 24.3	-25.5 ± 52.9	34.6 ± 28.3	28.8 ± 27.6
N008	-85.2 ± 324.4	254.2 ± 746.2	19.2 ± 28.0	-40.1 ± 48.9	0 ± 0	-4.0 ± 8.4
N009	-178.6 ± 106.4	-355.5 ± 372.2	4.7 ± 20.7	-55.5 ± 53.7	0 ± 0	14.4 ± 13.7
N010	-17.8 ± 26.9	171.3 ± 165.9	39.3 ± 11.4	-58.2 ± 49.2	0 ± 0	7.5 ± 9.0
N011	-134.6 ± 80.9	-262.3 ± 281.1	42.0 ± 20.7	-50.9 ± 51.3	0 ± 0	-0.1 ± 18.1
Experiment set 2						
L-DMPP	30.8 ± 52.0	67.4 ± 16.1	77.6 ± 5.4	66.9 ± 2.7	75.6 ± 3.7	66.0 ± 5.4
N012	-50.7 ± 59.5	9.5 ± 12.4	28.5 ± 15.8	0 ± 0	8.4 ± 7.9	14.6 ± 11.8
N013	121.2 ± 78.1	71.6 ± 34.3	87.3 ± 6.1	79.4 ± 3.6	89.5 ± 3.3	69.9 ± 4.5
N014	70.2 ± 67.2	77.0 ± 10.7	75.2 ± 5.4	56.8 ± 2.8	63.2 ± 17.4	44.3 ± 11.1
N015	48.9 ± 54.9	49.6 ± 11.8	34.0 ± 20.7	19.7 ± 16.2	0 ± 0	-12.7 ± 7.8
Experiment set 3						
H-DMPP	70.8 ± 56.6	40.4 ± 90.2	58.1 ± 41.8	65.0 ± 25.3	77.8 ± 21.0	67.0 ± 18.7
N002	-2.8 ± 83.0	-8.1 ± 99.5	50.7 ± 59.2	36.8 ± 40.5	55.1 ± 22.4	38.6 ± 22.6
N013	-45.2 ± 68.6	-13.0 ± 267.5	39.3 ± 146.6	37.2 ± 20.1	50.4 ± 17.8	57.0 ± 15.8
N016	-19.7 ± 63.0	20.4 ± 82.9	60.2 ± 75.3	48.3 ± 22.3	55.4 ± 18.9	40.0 ± 16.3
N017	58.8 ± 52.5	46.8 ± 87.4	70.5 ± 30.7	57.3 ± 23.2	56.4 ± 19.9	19.8 ± 19.6
Soil incubation at 35°C.						
H-DMPP	40.4 ± 147.7	-41.5 ± 30.6	29.1 ± 17.1	15.0 ± 14.4	-3.7 ± 21.5	44.7 ± 30.2
N002	58.4 ± 160.2	107.6 ± 37.3	94.4 ± 19.1	48.1 ± 11.7	61.6 ± 18.8	47.7 ± 27.1
N013	129.7 ± 195.6	79.8 ± 32.0	74.6 ± 16.5	40.0 ± 10.4	42.4 ± 17.4	34.6 ± 27.3
N016	63.3 ± 144.2	66.0 ± 30.3	55.2 ± 19.3	47.7 ± 10.0	71.0 ± 20.0	51.2 ± 27.5
N017	-59.4 ± 148.6	49.6 ± 29.2	50.9 ± 16.3	33.2 ± 10.6	35.6 ± 17.7	39.3 ± 26.5

[a] Application rates are in mol % of applied fertilizer N; for N001 - N017 = 10, L-DMPP = 1.5, M-DMPP = 3.6, H-DMPP = 10. [b] Calculated at each timepoint from NH_4^+-N loss percentages or NO_x^--N accumulation rates in NI-treated samples compared to the $(NH_4)_2SO_4$-control treatment. [c] Mean values (n = 3); errors are standard errors of the mean.

Negative inhibition values: See comment below Table S11.

4. Example R Script

An example script used to perform 2-way ANOVA utilising the *emmeans* package in R (version 3.5.2)

```
my_data <- read.csv("N018_N022_Dahlen_25deg_stats.csv")
my_data$Day <- factor(my_data$Day, levels = c(0,3,7,14,21,28), labels = c("D0", "D3", "D7",
"D14", "D21", "D28"))
str(my_data)
table(my_data$Treatment, my_data$Day)
data.lm <- lm(Ammonium_N ~ Day * Treatment, data = my_data)
anova(data.lm)
emmip(data.lm, Day ~ Treatment)
data.emm <- emmeans(data.lm, ~ Day * Treatment)
pairs(data.emm, simple = list("Treatment"))
CLD(pairs(data.emm, simple = list("Treatment")))
```

5. References

[1] R Core Team R: A language and environment for statistical computing. R Foundation for Statistical Computing, Vienna, Austria (2019); http://www.R-project.org/ (accessed 19/02/2019).

[2] Lenth, R. emmeans: Estimated Marginal Means, aka Least-Squares Means. R Package version 1.3.2.; https://github.com/rvlenth/emmeans (2019).

[3] Chambers, J. M. & Hastie, T. J. Statistical models in Statistical models in S (eds. Chambers, J. M. & Hastie, T. J.) 13-44 (Wadsworth and Brooks/Cole, 1992).
